# A global synthesis of ecosystem services provided and disrupted by freshwater bivalve molluscs

**DOI:** 10.1111/brv.12878

**Published:** 2022-06-30

**Authors:** Alexandra Zieritz, Ronaldo Sousa, David C. Aldridge, Karel Douda, Eduardo Esteves, Noé Ferreira‐Rodríguez, Jon H. Mageroy, Daniele Nizzoli, Martin Osterling, Joaquim Reis, Nicoletta Riccardi, Daniel Daill, Clemens Gumpinger, Ana Sofia Vaz

**Affiliations:** ^1^ School of Geography University of Nottingham University Park, Sir Clive Granger Building NG7 2RD Nottingham UK; ^2^ CBMA – Centre of Molecular and Environmental Biology, Department of Biology University of Minho Campus Gualtar 4710‐057 Braga Portugal; ^3^ Department of Zoology University of Cambridge Downing Street Cambridge CB2 3EJ UK; ^4^ Department of Zoology and Fisheries Czech University of Life Sciences Prague Kamýcká 129 Prague Czech Republic; ^5^ Departamento de Engenharia Alimentar, Instituto Superior de Engenharia and CCMAR Centre of Marine Sciences Universidade do Algarve Estr. da Penha 8005‐139 Faro Portugal; ^6^ Departamento de Ecoloxía e Bioloxía Animal, Facultade de Bioloxía Universidade de Vigo Campus As Lagoas – Marcosende 36310 Vigo Spain; ^7^ Norwegian Institute of Nature Research, Oslo Sognsveien 68 0855 Oslo Norway; ^8^ Department of Chemistry, Life Sciences and Environmental Sustainability University of Parma Viale delle Scienze, 11/A 43124 Parma Italy; ^9^ Department of Environmental and Life Sciences – Biology Karlstad University Universitetsgatan 2 651 88 Karlstad Sweden; ^10^ Faculdade de Ciências da Universidade de Lisboa MARE – Marine and Environmental Sciences Centre Campo Grande 1749‐016 Lisbon Portugal; ^11^ CNR‐IRSA Water Research Institute Corso Tonolli, 50 28922 Verbania Pallanza (VB) Italy; ^12^ blattfisch e.U. – Consultants in Aquatic Ecology and Engineering Gabelsbergerstraße 7 4600 Wels Austria; ^13^ CIBIO, Centro de Investigação em Biodiversidade e Recursos Genéticos, InBIO Laboratório Associado, Campus de Vairão Universidade do Porto 4485‐661 Vairão Portugal; ^14^ Departamento de Biologia, Faculdade de Ciências Universidade do Porto 4099‐002 Porto Portugal; ^15^ BIOPOLIS Program in Genomics, Biodiversity and Land Planning, CIBIO, Campus de Vairão 4485‐661 Vairão Portugal

**Keywords:** biofiltration, biomonitoring, *Corbicula*, cultural services, *Dreissena*, ecosystem services, freshwater mussels, provisioning services, regulating services, Unionida

## Abstract

Identification of ecosystem services, i.e. the contributions that ecosystems make to human well‐being, has proven instrumental in galvanising public and political support for safeguarding biodiversity and its benefits to people. Here we synthesise the global evidence on ecosystem services provided and disrupted by freshwater bivalves, a heterogenous group of >1200 species, including some of the most threatened (in Unionida) and invasive (e.g. *Dreissena polymorpha*) taxa globally. Our systematic literature review resulted in a data set of 904 records from 69 countries relating to 24 classes of provisioning (*N* = 189), cultural (*N* = 491) and regulating (*N* = 224) services following the Common International Classification of Ecosystem Services (CICES). Prominent ecosystem services included (*i*) the provisioning of food, materials and medicinal products, (*ii*) knowledge acquisition (e.g. on water quality, past environments and historical societies), ornamental and other cultural contributions, and (*iii*) the filtration, sequestration, storage and/or transformation of biological and physico‐chemical water properties. About 9% of records provided evidence for the disruption rather than provision of ecosystem services. Synergies and trade‐offs of ecosystem services were observed. For instance, water filtration by freshwater bivalves can be beneficial for the cultural service ‘biomonitoring’, while negatively or positively affecting food consumption or human recreation. Our evidence base spanned a total of 91 genera and 191 species, dominated by Unionida (55% of records, 76% of species), Veneroida (21 and 9%, respectively; mainly *Corbicula* spp.) and Myoida (20 and 4%, respectively; mainly *Dreissena* spp.). About one third of records, predominantly from Europe and the Americas, related to species that were non‐native to the country of study. The majority of records originated from Asia (35%), with available evidence for 23 CICES classes, as well as Europe (29%) and North America (23%), where research was largely focused on ‘biomonitoring’. Whilst the earliest record (from 1949) originated from North America, since 2000, annual output of records has increased rapidly in Asia and Europe. Future research should focus on filling gaps in knowledge in lesser‐studied regions, including Africa and South America, and should look to provide a quantitative valuation of the socio‐economic costs and benefits of ecosystem services shaped by freshwater bivalves.

## I. INTRODUCTION

The concept of Ecosystem Services (ESs), i.e. ‘the contributions that ecosystems make to human well‐being’ (Haines‐Young & Potschin, 2018, p. 9), has proven instrumental in galvanising public and political support for biodiversity conservation (MEA, 2005). Based on the latest *Common International Classification of Ecosystem Services* (CICES; Haines‐Young & Potschin, 2018), ESs include the provisioning of material and energy needs (provisioning ESs), non‐material characteristics of ecosystems that affect physical and mental states of people (cultural ESs), and regulation and maintenance of the environment for humans (regulating ESs). Although the importance of ESs extends beyond their economic value, estimates indicate an ESs worth of €171,521 million in the European Union for 2012 (Vysna *et al*., 2021), at least US$ 250 billion/year for pollination services globally (IPBES, 2019), and an expected worldwide loss of US$ 9.87 trillion by 2050 due to environmental change (Roxburgh *et al*., 2020). Even though much progress has been made (e.g. Green & Elmberg, 2014; Rodrigues *et al*., 2020; Brock, Cini & Sumner, 2021), understanding how different animal groups contribute to ESs is far from complete, particularly with respect to freshwater invertebrate taxa (Collier, Probert & Jeffries, 2016; IPBES, 2019).

Freshwater bivalves (FBs) are among the most abundant groups of invertebrates in freshwater ecosystems globally and can make up more than 90% of the benthic (i.e. bottom‐dwelling) biomass (Okland, 1963; Sousa *et al*., 2008). FBs comprise >1200 species spread across taxonomically and biologically distinct groups, with most species (72%) belonging to the strictly freshwater order Unionida (Lopes‐Lima *et al*., 2018). Due to their (semi)infaunal, suspension‐feeding life habit and complex life cycle, involving a parasitic larval stage, Unionida are particularly sensitive to anthropogenic habitat degradation and represent one of the most threatened taxonomic groups. Forty species are already presumed extinct (Lopes‐Lima *et al*., 2018; IUCN, 2021), including eight species recently declared extinct by the U.S. Fish and Wildlife Service (The Center for Biological Diversity, 2021). By contrast, several notoriously invasive species are known within the Unionida (e.g. *Sinanodonta woodiana*) and more commonly, non‐unionid FB orders, including the Ponto‐Caspian myoid zebra and quagga mussels (*Dreissena polymorpha*, *Dreissena bugensis*), the veneroid Asian clam (*Corbicula fluminea*) and the mytiloid golden mussel (*Limnoperna fortunei*), native to East and Southeast Asia (Sousa *et al*., 2014). These species often displace or replace native FB species in invaded regions, which has resulted in severe population losses of Unionida in, for example, the Laurentian Great Lakes and large stretches of European mainland river basins (Strayer, 1999; Sousa *et al*., 2014). Owing to their different life histories and ecology, invasive FBs often attain much greater biomass than native FBs and can cause severe ecological and economic damage (Strayer, 1999; Sousa *et al*., 2014; Haubrock *et al*., 2022).

Research and conservation of FBs is commonly justified by the important ecosystem functions and services that they provide (Lopes‐Lima *et al*., 2018). However, the available scientific evidence for FB–ES associations has never been comprehensively reviewed and systematised. Adopting the ES designations of the *Millennium Ecosystem Assessment* (2005), Vaughn (2018) identified three provisioning (i.e. food for humans, food for other species, products from shells), two cultural (i.e. cultural and existence value), one regulating (i.e. biofiltration) and four supporting ESs (i.e. nutrient cycling and storage, habitat/habitat modification, environmental monitoring, food webs) provided by FBs (see also Vaughn & Hoellein, 2018). Whilst these works represent an important first step towards assessing the ESs of FBs, they are not comprehensive, largely restricted to a single continent (North America) and taxonomic group (Unionida), and strongly focused on supporting services, which are not regarded as ESs *per se* by CICES (Haines‐Young & Potschin, 2018). In addition, the available evidence on FBs disrupting rather than providing ESs has never been reviewed to date, although attempts have been made to quantify both ES provisions and disruptions, particularly of non‐native FBs (Limburg *et al*., 2010).

A comprehensive, systematic review of the global scientific evidence is needed not only to provide a more complete understanding of the ESs provided and disrupted by FBs, but also to understand temporal and geographic trends across specific ESs and taxa, and identify gaps in our current knowledge and areas of importance for future research. Considering the ongoing and predicted future spread of non‐native FBs in many regions of the world (Gallardo *et al*., 2018; Petsch *et al*., 2021), a better understanding of the differences and similarities in ESs provided and disrupted by native and non‐native species is therefore of particular urgency.

This study provides a systematic review of the available evidence for ESs associated with FBs. Its specific objectives were to (*i*) synthesise ESs that are associated with FBs; (*ii*) quantify the available evidence across temporal and geographic scales, taxonomic groups, native *versus* non‐native species, and types of ESs; and, on that basis; (*iii*) derive novel insights into the importance of FBs to humans; and (*iv*) identify current shortcomings in our knowledge and recommend future directions for research.

## II. LITERATURE SEARCH AND REVIEW

### (1) Data collection

Data on published evidence of associations between FBs and ESs were derived from a scientific literature search conducted in *ISI Web of Science* (ISI WOS; http://webofknowledge.com/) and *Scopus* (https://www.scopus.com/) search engines in June 2020 and updated in March 2021, using an exhaustive compilation of search terms (Appendix S1). Search terms referring to FBs included common terms/names [e.g. ‘freshwater’ AND (‘mussel’ OR ‘clam’)], and scientific names of all known bivalve genera as well as species within predominantly marine genera that complete their life cycles in fresh water as provided by Graf & Cummings (2021). Search terms referring to ESs included general terms (e.g. ‘environment* function*’ OR ‘ecosystem service’) as well as terms referring to specific ESs that are potentially associated with FBs, including provisioning (e.g. ‘food’ OR ‘material’), regulating (e.g. ‘water quality’ OR ‘biological control’) and cultural (e.g. ‘ornamental’ OR ‘recreation’) ESs (Haines‐Young & Potschin, 2018). The search string started with the most general terms on FBs and ESs, followed by step‐by‐step addition of new, more specific terms. If the new search resulted in a higher number of outputs, the new term was retained, otherwise it was removed. Studies written in English, German, French, Italian, Romanian, Portuguese, Spanish, Czech and Slovakian, and published up to and including 31 December 2020 were considered.

Published studies retrieved *via* ISI WOS and *Scopus* searches were combined, and duplicates removed. Relevant records (i.e. pieces of evidence for an association between FB(s) and a specific ES) were identified by applying the following inclusion and exclusion criteria. In general, only records that reported primary evidence for an association between FBs (i.e. species/genera that complete their life cycles in fresh water) and ESs were retained. Therefore, records based on secondary evidence (e.g. in literature reviews or meta‐analyses) were excluded to avoid double‐counting. Definition of ESs followed CICES (Haines‐Young & Potschin, 2018) and thus, evidence for ecological functions of FBs without explicit implications on human well‐being were excluded.

To allow reproducibility, ESs reported in the literature were classified following CICES (Haines‐Young & Potschin, 2018) employing a three‐level hierarchical system (Table 1). In addition, we extracted data on the geographic location (i.e. country/region and continent; Appendix S2) and taxonomic name(s) (i.e. order, genus and species) of FBs from each record where available. Based on that information, the FB taxon/taxa of each record was/were categorised as native or non‐native to the country of study using information on native species distributions from Graf & Cummings (2021) and CABI (2021). Finally, information was extracted from each record on whether the FB(s) was/were reported by the authors as a provider and/or disruptor, i.e. promoting and/or diminishing the quality or quantity of the respective ES.

**Table 1:** Hierarchical classification and description of ecosystem services (following Haines‐Young & Potschin (2018)) found to be associated with freshwater bivalves in the published literature.

Section	Group	Class (CICES code)	Description	% of records
Provisioning	Energy	Energy production (1.1.4.3)	Bivalves used in the provision of energy for human use	<1
	Materials	Material production (1.1.4.2; 1.1.6.2)	Bivalves or their parts used to support the production of other materials (excluding ornaments)	2
		Tools (1.1.4.2; 1.1.6.2)	Bivalves or their parts used as tools (excluding ornaments)	2
	Medicinal	Biophysical products (1.1.4.2; 1.1.6.2)	Use of biophysical products of bivalves (excluding genetic material) for medicinal or therapeutic purposes	2
		Genetic/protein information (1.2.2.3)	Use of genetic or protein information from bivalves for medicinal or therapeutic purposes	3
	Nutrition	Captured food (1.1.6.1)	Bivalves captured in the wild as food for direct human consumption	6
		Cultivated food (1.1.4.1)	Bivalves cultivated as food for direct human consumption	2
		Food production (1.1.4.1; 1.1.6.1)	Bivalves (captured or cultivated) influencing the production of other edible organisms supporting human diets	4
Cultural	Aesthetic experiences	Ornamental (3.1.2.4)	Bivalves or their parts providing ornamental benefits to humans	3
	Attitudes and other interactions	Spiritual, symbolic and religious (3.2.1.1; 3.2.1.2)	Bivalves or their parts providing symbolic, spiritual or religious meaning to society	1
		Tradition (3.1.2.3)	Characteristics of bivalves that are resonant in terms of cultural heritage and traditions of human communities	1
	Knowledge	Archaeology (3.1.2.1)	Bivalves enabling acquisition of knowledge about past human societies and communities	4
		Biomimicry (3.1.2.1)	Use of bivalve morphology, physiology and/or behaviour in the design and production of other materials	<1
		Biomonitoring (3.1.2.1)	Bivalves enabling acquisition of knowledge about water quality for human benefit	34
		Paleoenvironment (3.1.2.1)	Bivalves enabling acquisition of knowledge about past environments	9
		Social education (3.1.2.1)	Bivalves contributing to social education and training	<1
	Physical interactions	Recreation (3.1.1.1; 3.1.1.2)	Bivalves affecting human physical interactions with nature, including leisure and recreational activities	1
Regulating	Regulation of organisms	Algae (2.2.3.1)	Bivalves affecting prevalence and concentration levels of algae, including blue‐green algae (cyanobacteria), that affect human health and amenity value of waterbodies	7
		Bacteria (2.2.3.1)	Bivalves affecting prevalence and concentration levels of bacteria that affect human health and security	2
		Diseases (2.2.3.2)	Bivalves affecting prevalence and concentration levels of human diseases (e.g. gastroenteritis) due to interactions with pathogenic organisms (e.g. viruses, protozoan parasites) that affect human health and security	1
	Mediation of human inputs	Filtration and sequestration (2.1.1.2)	Bivalves filtering, sequestering, accumulating or storing harmful wastes of human origin	8
		Transformation (2.1.1.1)	Bivalves transforming or decomposing harmful wastes of human origin	1
	Physico‐chemical regulation	Storage and excretion (2.2.5.1; 2.2.5.2)	Bivalves contributing to removal or addition of organic or inorganic substances (e.g. sediments and nutrients) with implications for recreational activities or human health	4
		Water clearance (2.1.2.1; 2.1.2.3)	Bivalves changing the physical properties of water quality with implications for recreational activities and/or human health	3

### (2) Data visualisation

The number of records per year of publication was plotted as smoothing curves (averages for 2‐year time periods) between 1949 (the first case study reported in our data set) and 31 December 2020. Sankey diagrams were generated to illustrate the relative quantity of and linkage among records on different ES categories based on different continents of study, status (native *versus* non‐native), effect (provider *versus* disrupter) and/or taxonomic order of study species using the R‐package ‘d3Network’ (Gandrud, 2015).

## III. GENERAL DESCRIPTION OF THE DATA SET

**Fig. 1:**
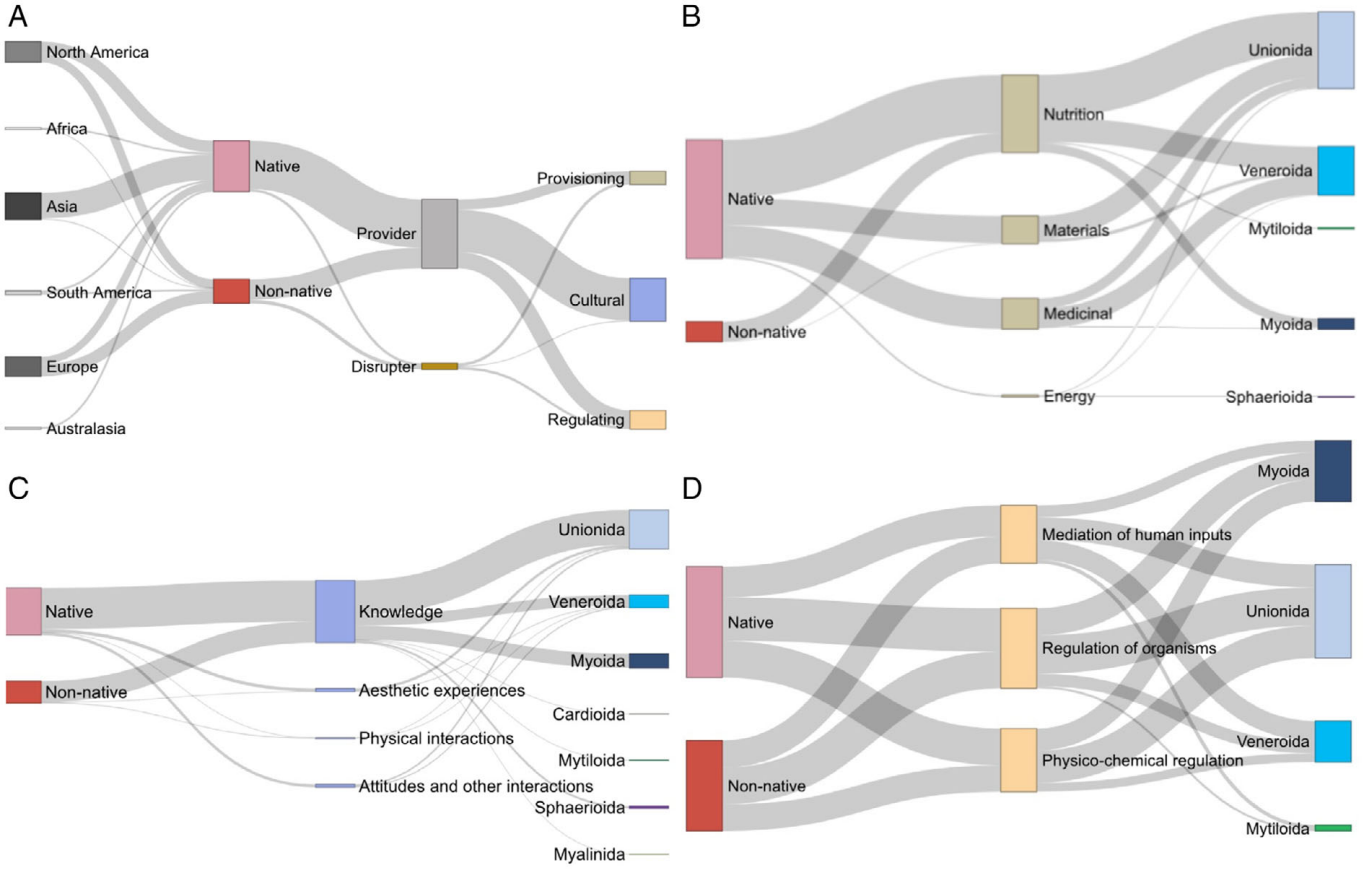
Linkages among the relative quantity of published records providing primary evidence for an association between freshwater bivalves and specific sections and groups of ecosystem services (sensu Haines‐Young & Potschin, 2018) based on different continents of study and/or status (native *versus* non‐native), effect (provider *versus* disrupter) and/or taxonomic order of study species. (A) Linkages among continent of study, status and effect of species, and ecosystem service section across all 904 published records. (B–D) Linkages among status and taxonomic order of species, and ecosystem service‐group of (B) provisioning (*N* = 189), (C) cultural (*N* = 491) and (D) regulating (*N* = 224) services.

A total of 6745 published studies were retrieved using our search terms. After the application of exclusion and inclusion criteria, the final data set comprised 684 studies (see Appendix S3 for a full list of studies) and 904 records (i.e. pieces of evidence for association between FBs and specific ES classes as defined in Table 1). In total, we identified evidence for 24 CICES classes of provisioning, cultural and regulating ESs that are associated with FBs (Table 1). A total of 146 studies provided evidence for more than one ES class, with up to five records being extracted from a single publication. Cultural ESs were the most represented (*N* = 491), followed by regulating (*N* = 224) and provisioning services (*N* = 189) (Fig. 1A; Table 1). Evidence was strongly skewed towards FBs as providers (91%) rather than disrupters (9%), with evidence for disruption being largely restricted to provisioning (~5%) and regulating ESs (~4%; Fig. 1A).

### (1) Taxonomic trends

Evidence came from a total of 91 genera and 191 species across seven bivalve orders, dominated by Unionida (76% of species), followed by Sphaerioida and Veneroida (each 9%), Myoida (4%), Cardioida, Myalinida and Mytiloida (each <1%). Whilst available evidence was also largely based on Unionida (55% of records), a considerable proportion of records related to Veneroida (21%) and Myoida (20%), respectively. At the genus and species levels, records were strongly skewed towards non‐Unionida. Thus, 20% of records referred to each of the myoid genus *Dreissena* and the veneroid genus *Corbicula*, respectively, followed by the unionid genera *Unio* (11%) and *Sinohyriopsis* (4%). The most represented species in the data set were *D. polymorpha* and *C. fluminea* (18% of records each), followed by the unionid *Sinohyriopsis cumingii* (4%). Whilst almost two thirds (62%) of records referred to species that were native rather than non‐native (38%) to the respective country of study, 60% of records on disrupting ESs were based on non‐native species (Fig. 1A). Records pertaining to non‐native species were largely dominated by the genera *Dreissena* (62%) and *Corbicula* (28%), and additionally included *Sinanodonta* (5%, Unionida), *Limnoperna* (4%, Mytiloida), *Amblema* (<1%, Unionida) and *Mytilopsis* (<1%, Myoida). The vast majority of records on *Dreissena* and *Limnoperna* originated from countries where these genera are not native (i.e. 95 and 88% of records, respectively), whilst this was true only for about half of the records on *Sinanodonta* (51%) and *Corbicula* (42%).

### (2) Geographical trends

**Fig. 2:**
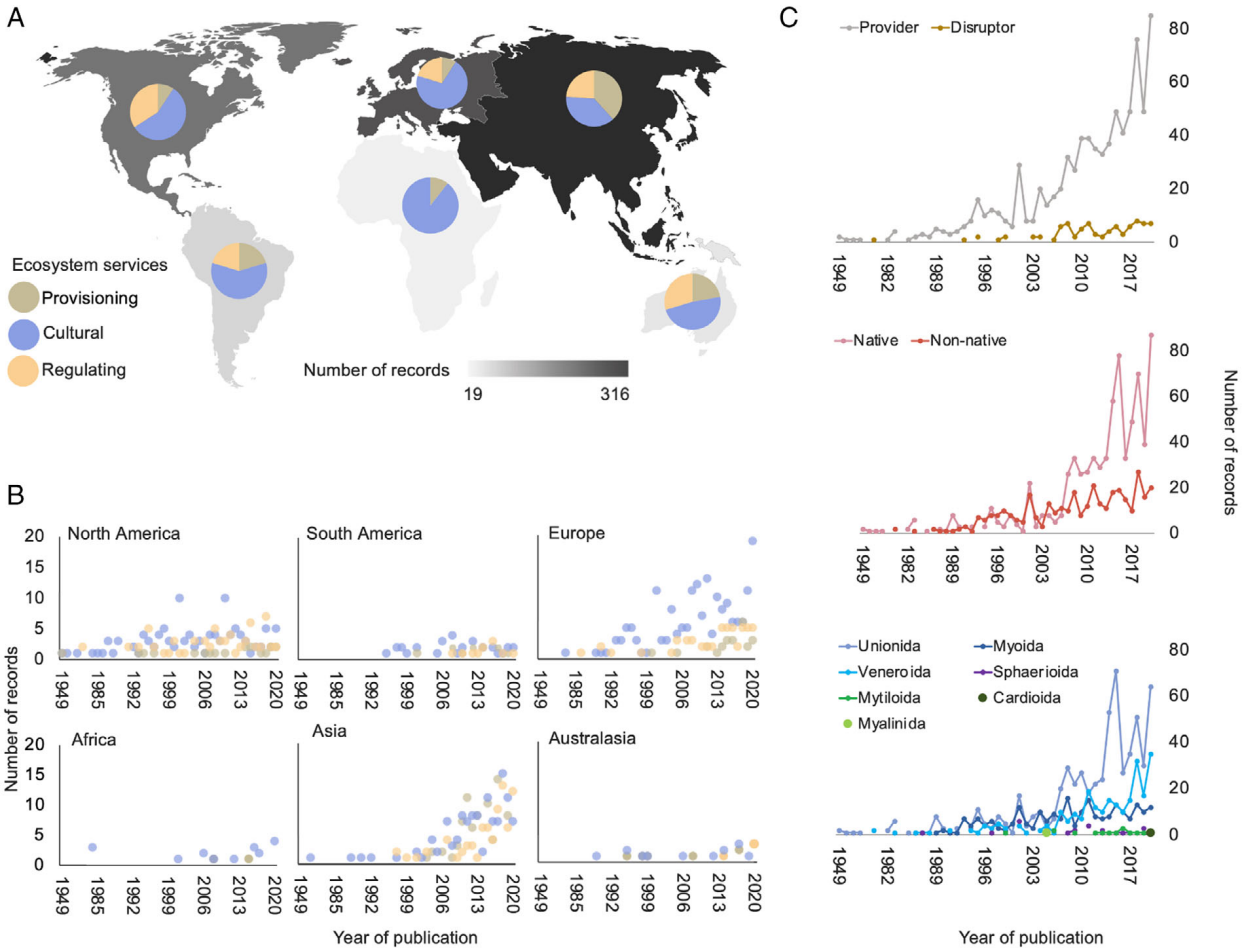
Geographic and temporal patterns of published records providing primary evidence of an association between freshwater bivalves and specific sections of ecosystem services (sensu Haines‐Young & Potschin, 2018). (A) Heatmap of total number of records per continent with pie charts showing the relative proportions of the three ecosystem service sections. (B,C) Number of records per year of publication (B) per continent grouped by ecosystem service sections, and (C) grouped based on whether freshwater bivalves acted as ecosystem service providers or disruptors (top panel), bivalve status (native or non‐native; middle panel), and taxonomic order (bottom panel). Values from 1949 to 1985 are condensed along the *x*‐axes relative to those from 1985 to 2020 for ease of visualisation.

Evidence was collected from a total of 69 countries, with 18% of records originating from the USA, followed by China (13%), France and Canada (each 5%). On the continent level, most records originated from Asia (35%), Europe (29%) and North America (23%), whilst records from Africa (2%), Australasia (3%) and South America (6%) were scarce (with 2% of records not providing this information; Figs 1A and 2A). Research has been strongly focused towards cultural ESs in Africa, Europe, North America, South America and Australasia (48–89% of overall records), whilst in Asia, provisioning, regulating and cultural ESs contributed to records in almost equal amounts (Fig. 2A). Asia also provided the widest range of evidence, with available evidence for 23 of the 24 CICES classes (i.e. all except ‘Social education’; Table 1) compared with lower coverage from Europe (19), North America (16), South America (14), Australasia (9) and Africa (7). Records from Asia, Africa and Australasia were almost exclusively focused on native species (>95%), whilst a considerable proportion of records from Europe, North America and South America related to non‐native species (<52% natives) (Fig. 1A).

### (3) Temporal trends

Annual publication of studies reporting FB–ES associations generally increased through the years, with the earliest found paper published in 1949 (Fig. 2B,C). The first evidence for non‐native species was published in 1980 (Fig. 2C). The rate of acquisition of evidence for non‐native species increased markedly in the early 1990s and has been increasing relatively steadily since (Fig. 2C). The rate of record publication on native species and Unionida increased considerably in the mid 2000s (Fig. 2C). Since the turn of the century, annual record output remained relatively stable for the Myoida (dominated by usually non‐native *Dreissena* spp.) and Sphaerioida, but increased notably for Veneroida (largely dominated by *Corbicula* spp.; Fig. 2C).

Whilst the first record from North America dates from 1949, first records from the other continents are more recent, spanning from 1977 (Asia) to 1982 (Europe), 1983 (Africa), 1989 (Australasia) and 1995 (South America) (Fig. 2B). Annual publication of ES records from North America has been relatively constant for the past 20+ years, while records from Europe have increased over the same period, particularly with regard to cultural ESs (Fig. 2B). Rate of publication for Asia has been increasing rapidly for the past ten years across all three ES sections (Fig. 2B).

## IV. PROVISIONING ECOSYSTEM SERVICES

Based on our database, FBs are associated with four groups of provisioning ESs (PESs), i.e. Nutrition (56% of PES records), Medicinal (24%), Materials (19%) and Energy (1%) (Table 1, Fig. 1B). Records on PESs are disproportionately associated with native rather than non‐native species, and better represented by Veneroida (*Corbicula*) and Unionida rather than Myoida (*Dreissena*), Mytiloida and Sphaerioida (compare Fig. 1B with Fig. 1A,C,D). With respect to PES groups, native species were disproportionately associated with Materials and Medicinal PESs, whilst non‐native species were disproportionately associated with Nutrition (Fig. 1B) and disruption of PESs (Fig. 1A). Trends were also apparent across taxa, e.g. Veneroida (*Corbicula*) was disproportionately associated with nutritional and medicinal PESs, Unionida with Nutrition and Materials, and Myoida (*Dreissena*) with Nutrition (Fig. 1B).

### (1) Nutrition

Nutritional PESs fulfilled by FBs include the direct use of FBs captured in the wild (30%) or cultivated (8%) as a food source for humans, and the association of FBs with the production of other edible organisms supporting human diets (18%) (Table 1).

#### (a) Captured and cultivated food

**Fig. 3:**
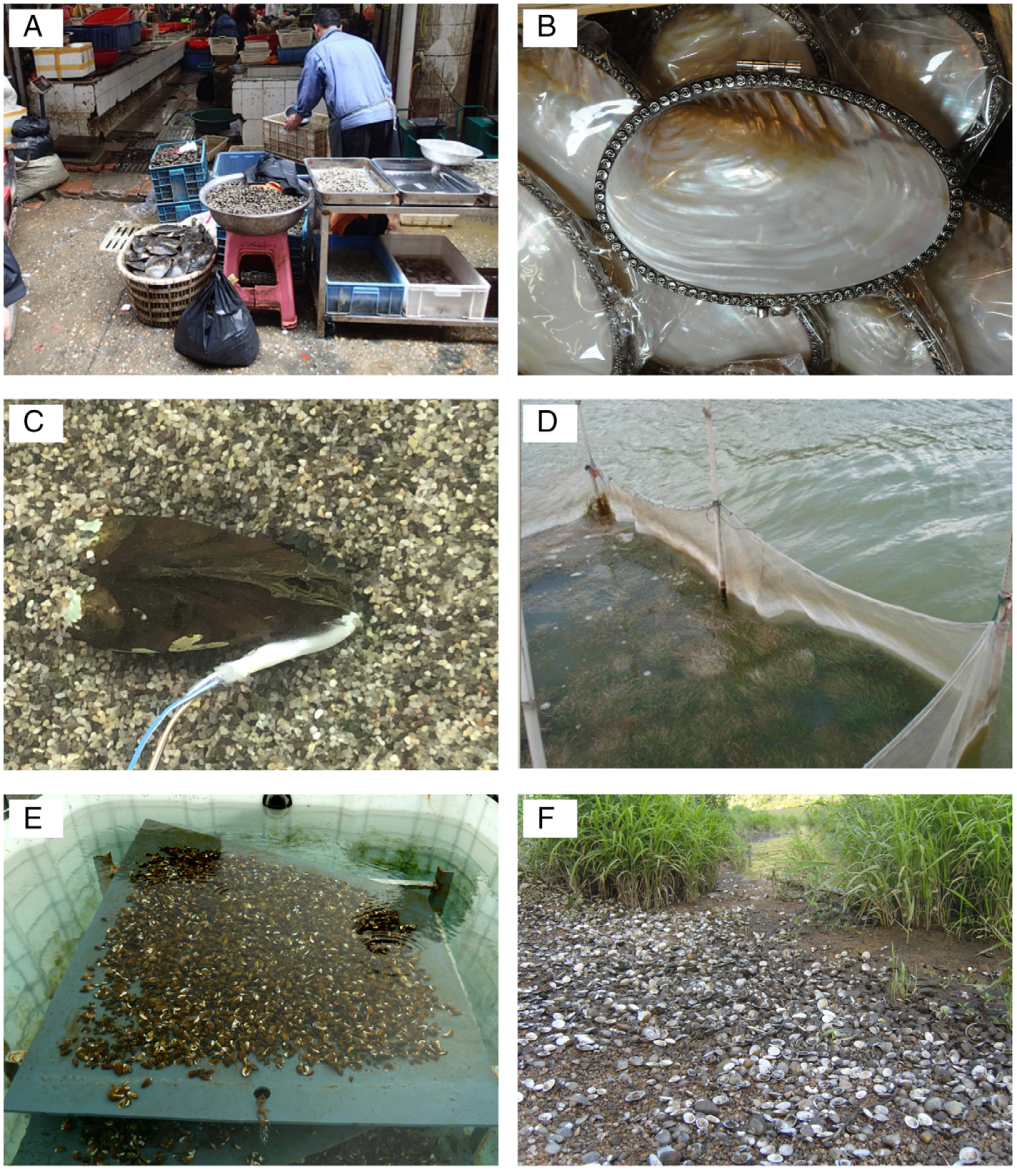
Examples of freshwater bivalves providing (A–E) and disrupting (F) ecosystem services. (A) Freshwater bivalves from Poyang Lake, China, being sold as a food source (credit: K. Douda). (B) Ornamental purse made of the nacreous layer of *Cristaria* sp. for sale at Chatuchak Market, Bangkok, Thailand (credit: U. Kovitvadhi & S. Kovitvadhi). (C) *Unio elongatulus* equipped with valvometer used for biomonitoring of water quality (credit: N. Riccardi); (D) Effect of *Sinadondonta woodiana* on water clarity in Lake Dianchi, China. Mussels were placed into the enclosure and their filtration improved water clarity to the extent that bottom‐rooting macrophytes could establish from the seed bank on the lake bed (credit D.C. Aldridge). (E) Non‐native *Dreissena polymorpha* attached on acrylic glass panels, which were inserted in a pilot‐plant in Milan, Italy, resulting in the removal of pharmaceuticals, drugs of abuse and heavy metals from civil wastewaters (credit: A. Binelli). (F) Disruption of the recreational value of the beach of the Minho River, Portugal, by dense aggregations of non‐native *Corbicula fluminea* shells after a massive die‐off event (credit: R. Sousa).

Captured and, in some cases, cultivated veneroid and unionid species provide an important food source for – often vulnerable – communities across large parts of Asia, including Cambodia (Ngor *et al*., 2018), China (Zeng *et al*., 2019; Fig. 3A), India (Sonowal & Kardong, 2020), Indonesia (Lukman *et al*., 2019), Japan (Horikoshi, 2020) and Malaysia (Zieritz *et al*., 2018*a*; Rak *et al*., 2020*a*), as well as, for example, Western Africa (Akélé *et al*., 2015). On other continents, records on FBs as a source of food are often historical, referring to, for example, ancient/indigenous communities in North America (Theler & Hill, 2019), Australia (Garvey, 2017) and Europe (Nicodemus, 2011). However, due to high bioaccumulation rates in FBs, consumption of FBs from polluted sites can also have adverse effects on human health due to, for example, elevated persistent organic pollutant levels (Takabe *et al*., 2012), and heavy metal (Ghosh *et al*., 2020*b*) and radionuclide poisoning (Martin *et al*., 1998) (Fig. 1A).

#### (b) Food production

The effect of FBs on other edible organisms or other food can be beneficial as well as disruptive for human well‐being. Examples for beneficial ES effects include the use of FBs, including non‐native species, as chicken feeds (McLaughlan, Rose & Aldridge, 2014; Bayerle *et al*., 2017), as diffusers of antibiotic resistance in trout aquaculture (Sicuro *et al*., 2020), and – in the form of shell powder – as antimicrobial agents in soybean curd preservation (Yang *et al*., 2019). Disruptive effects in this respect mostly refer to non‐native FBs, including the observed decrease of commercially harvested fish following introduction of *Dreissena* spp. to habitats in North America (Fera, Rennie & Dunlop, 2017; Hansen *et al*., 2020) and reduced productivity of shrimp farms in Colombia due to the invasive myoid *Mytilopsis trautwineana* (Aldridge *et al*., 2008).

### (2) Materials

Records refer to the use of FBs or their parts in the production of other materials (11%) or tools (7%) without explicit or exclusive ornamental value (Table 1).

#### (a) Material production

Notable examples include studies investigating the potential application of FB shells or their derivatives as filling material in construction (Li *et al*., 2013*b*; Chakraborty *et al*., 2020), as adsorbents of dyestuffs (Figueiredo, Loureiro & Boaventura, 2005) or purifiers of peat water (i.e. *via* hydroxyapatite synthesised from *Corbicula moltkiana* shells; Alif, Aprillia & Arief, 2018). Evidence for actual use of FBs in material production is relatively scarce, with the use of nacre in button production arguably being the most significant, particularly with respect to historical communities (Haag, 2012; Strack, 2015; Sakalauskaite *et al*., 2019) but also including contemporary societies, such as fishermen in the Tocantins River, Brazil (Beasley, 2001).

#### (b) Tools

Evidence for the use of FBs as tools is completely restricted to historical communities, pertaining to the use of shells and pearls as, for example, knives, scrapers (e.g. to skin animal hides, scaling of fish, scraping plant material for string/fishnet production) and tools for shaping pottery vessels during the Pleistocene and pre‐historic times across all continents (Leechman, 1949; Jackson & Jackson, 2008; Romanus *et al*., 2008; Debruyne, 2010; Weston, Szabó & Stern, 2017; Mărgărit, Mirea & Radu, 2018).

### (3) Medicinal

Records pertaining to the use of FBs for medicinal purposes refer to genetic/protein information (15%) and biophysical products (10%) (Table 1), and originate almost exclusively (89%) from Asia. A large number of studies in this category are based on *C. fluminea* from Eastern Asia (China, Taiwan, Japan), where this species has been used in Traditional Chinese Medicine and has been shown to exhibit anti‐inflammatory (Yeh *et al*., 2017), neuroprotective (Hsieh *et al*., 2017), hypotensive (i.e. reducing blood pressure; Tsai *et al*., 2006), hepato‐protective and antioxidant effects (Bai *et al*., 2020), and the ability to reduce cholesterol levels (Chijimatsu *et al*., 2011) and improve wound healing (Peng *et al*., 2017). Some notable examples of medicinal ESs of other FB species include therapeutic effects of *Pisidium coreanum* and *Lamellidens marginalis* on bone diseases (osteoporosis and arthritis, respectively) (Chakraborty *et al*., 2010; Choi *et al*., 2019), and properties of *S. cumingii* concerned with wound repair (Dai *et al*., 2010) and immunoenhancement (Qiao *et al*., 2016). An example for the medicinal use of a biophysical FB product is the use of shells or their derivatives in dental implants (Wang *et al*., 2006; Zhu *et al*., 2011).

### (4) Energy

Finally, two studies from Asia provided evidence for the potential use of FBs in energy provision, including the suitability of *S. woodiana* shells as an economic catalyst for biodiesel (Chinese tallow oil) production (Hu, Wang & Han, 2011).

## V. CULTURAL ECOSYSTEM SERVICES

The vast majority of records for cultural ESs (CESs) are concerned with the acquisition of knowledge from FBs (using FBs as instruments rather than objects of research; 88%), whilst evidence for their relevance to aesthetics (6%), people's attitudes and other interactions (5%), and physical interactions (1%) is comparatively scarce (Fig. 1C, Table 1). Representation of non‐native species was largely confined to knowledge acquisition, whilst evidence for FBs providing benefits in terms of attitudes, aesthetics and other interactions was almost exclusively based on native species (Fig. 1C).

### (1) Knowledge

Within this group of studies, most records were associated with the use of FBs for the acquisition of knowledge about current water quality (i.e. CICES class ‘biomonitoring’; 63%), but also on palaeoenvironments (16%), past human societies and communities (‘archaeology’; 8%), optimisation of production of other materials (‘biomimicry’; 1%) and social education (<1%) (Table 1).

#### (a) Biomonitoring

Due to their high filtration capacity and consequently, bioaccumulation rates, coupled with often high population densities that facilitate the collection of adequate replicate samples (Vaughn, 2018), FBs are popular organisms in biomonitoring studies that assess spatial and temporal patterns of water quality, including the location of pollution sources and quantification of the effects of remedial (e.g. establishment of a water treatment plant) or destructive actions (e.g. pesticide spill). Non‐native species, in particular, *D. polymorpha*, *D. bugensis* and *C. fluminea* in Europe and North America, are commonly used in this context due to their ability to survive in poor water quality habitats (Sousa *et al*., 2014).

Many of these records are based on the quantification of concentrations in mussel tissues and/or shells of, for example, heavy metals (Johns, 2001; Lukashev, 2008; Reis *et al*., 2014; Labuschagne *et al*., 2020), organic/chemical compounds (e.g. insecticides, pesticides, organotins, persistent organic compounds, organic halogens, polycyclic aromatic hydrocarbons, hormones) (Hayer, Wagner & Pihan, 1996; Regoli *et al*., 2001; Richman & Somers, 2010; Khazri *et al*., 2016; Bai & Acharya, 2019; Lécrivain *et al*., 2020), microplastics (Su *et al*., 2018) and radionuclides (Bollhöfer, 2012). In other studies, authors provide information about water quality by quantifying biomarkers that are proxies for physiological stress, including glutathione S‐transferase, glutathione reductase activity or DNA damage (de Lafontaine *et al*., 2000; Binelli *et al*., 2006; Contardo‐Jara & Wiegand, 2008; Michel *et al*., 2013; Klimova *et al*., 2017; Bonnail *et al*., 2018; Bonnail, Macías & Osta, 2019*a*). Behavioural and physiological indicators, such as valve movement and heart rate recovery time, have become increasingly popular tools for early detection and real‐time monitoring of water quality (Fig. 3C; Mouabad *et al*., 2001; Tran *et al*., 2007; Chen *et al*., 2010; Jou *et al*., 2016; Zarykhta *et al*., 2019), and are commercially available (de Zwart, Kramer & Jenner, 1995). FBs have also been suggested as early warning systems for the presence of (neuro)toxins [e.g. those produced by phytoplankton (Wood *et al*., 2006; Lepoutre *et al*., 2020*b*)], and human pathogens and diseases, such as microsporidia (Lucy *et al*., 2008), *Escherichia coli* (Selegean *et al*., 2001), and other bacteria (Graczyk *et al*., 2001) and protozoa (Géba *et al*., 2020*a*). Other, more scarcely applied indicators used in FB biomonitoring of water quality include simple presence/absence of (indicator) species (Chazanah *et al*., 2020), morphological condition indices, including growth rates (Cataldo *et al*., 2001*a*; Thitiphuree *et al*., 2013), and Na:Cl ratios in shells as indicator of road salt pollution (O'Neil & Gillikin, 2014). In some cases, long‐term monitoring programs utilising one or more of the indicators described above (e.g. concentrations in tissue/shells, valve movement) have been put in place using caged FBs, e.g. in pulp and paper mill recipient watercourses of Finland (1984–1998 using *Anodonta anatina*; Herve, Paasivirta & Heinonen, 2001), in Lake Maggiore, Italy (1996–2008 using *D. polymorpha*; Riva *et al*., 2010), and in the Niagara River, North America (1983–2009 using *Elliptio complanata*; Richman *et al*., 2011).

#### (b) Palaeoenvironment

Due to their calcareous shells, FBs can also be used as archives of past environmental conditions (Vaughn, 2018). In their simplest form, these palaeoenvironmental reconstructions are based on data on the presence or relative abundances of FB species, which are used to infer the prevalent climatic, hydrological or other environmental conditions at the time and site. Many of these records refer to relatively recent, i.e. Holocene (0.01–0 Mya) and Pleistocene (2.6–0.01 Mya), deposits, including those from Turkey (Kuzucuoğlu *et al*., 1997), Lithuania and Belarus (Sanko, Gaigalas & Yelovicheva, 2011), the Arabian Peninsula (Matter *et al*., 2015) and China (Noda *et al*., 2007). Other records refer to older strata, starting from the Pliocene [5–2.6 Mya; e.g. Omo‐Turkana Basin, Kenya (Van Bocxlaer, 2020)], to the Miocene [23–5 Mya; e.g. Austria (Harzhauser & Tempfer, 2004) and the Andes (Cadena & Casado‐Ferrer, 2019)], and as far back as the Oligocene (34–23 Mya), Eocene (56–34 Mya), Cretaceous (145–66 Mya) and Jurassic (201–145 Mya) of e.g. North America (Pierce & Constenius, 2001; Good, 2004; Montgomery & Barnes, 2012) and the British Isles (Andrews & Walton, 1990). In a few cases, authors have used intraspecific variation in morphological characteristics, such as the shape of the shell (Eagar, 1948) or relative growth rate (Black *et al*., 2010), to reconstruct palaeoenvironments. However, considering the incomplete understanding of how shell shape, size and sculpture is related to specific environmental conditions and functions in and across contemporary species (Zieritz & Aldridge, 2009; Levine, Hansen & Gerald, 2014), the value of fossilised specimens in this respect is currently restricted.

A rather widespread approach is the use of stable isotope ratios (mostly of oxygen and/or carbon) in (sub)fossil FB shells to reconstruct palaeoenvironmental (mostly palaeoclimatic) conditions. These usually refer to the Holocene, e.g. from Egypt (Hassan *et al*., 2012), Hungary (Schöll‐Barna *et al*., 2012), Italy (Baroni *et al*., 2001), Argentina (Pérez *et al*., 2020) and the USA (Tevesz *et al*., 1997; Yu *et al*., 2008), and less frequently, to earlier epochs (e.g. Eocene USA; Buskirk *et al*., 2016). The regularity of annual shell formation of FBs in non‐tropical regions, leading to the formation of visible annual shell rings, further provides the opportunity to evaluate changes in e.g. isotope composition at high temporal (i.e. annual or even intra‐annual seasonal) resolution. Such a ‘sclerochronological’ approach has been applied on (sub)fossil shells, e.g. from Holocene Syria (Çakirlar & Şeşen, 2013), Holocene Turkey (Lewis *et al*., 2017) and Miocene Amazon (Kaandorp, Wesselingh & Vonhof, 2006), but also on contemporary specimens, such as *Margaritifera margaritifera*, which can attain lifespans of >200 years (Schöne *et al*., 2020). In a combined sclerochronological analysis of archaeological, recent and contemporary FB shells (*Amblema plicata* and *Quadrula quadrula*), Fritts *et al*. (2017) constructed an environmental archive of the Illinois River, USA, spanning the past 1000 years.

Evidence of the use of FBs in palaeoenvironmental reconstructions *via* other indicators includes the reconstruction of past metal concentrations (Binkowski *et al*., 2019), and the reconstruction of palaeobasins through phylogeographical analyses (Hewitt *et al*., 2018).

#### (c) Archaeology

Evidence for FBs providing information on historical human societies and communities is based on the presence, abundance or isotopic signature of species/specimens. Several studies provide evidence for the historic use of FBs as food, tools, ornaments and/or objects of spiritual, symbolic or religious meaning [e.g. *Nodularia douglasiae* from Neolithic China (Li *et al*., 2013*a*); *Unio* sp. in Eneolithic Romania (Mărgărit, 2020)]. In other cases, information on other aspects of past human societies are provided through FBs. For example, the isotopic signature (carbon and oxygen) of ornamental disc beads made from *Unio* shells from several Neolithic burials in Poland revealed that these originated from both riverine and lacustrine environments, which suggests changes in the shell source over time and thereby provided evidence for the existence of a regional exchange network (Apolinarska & Kurzawska, 2020). In Burleigh (1983), an Egyptian tomb was dated to ~5000 ya using radiocarbon data from an *Etheria elliptica* shell found in the tomb. Finally, a number of studies utilise palaeoenvironmental and palaeoclimatic information reconstructed through stable isotope data from FBs to infer information on ancient human ecologies. An example is the study by Çakirlar & Şeşen (2013), who used sclerochronological isotope data from archaeological *Unio* sp. shells to show that continuous perennial flow of the Jaghjagh River, Syria, throughout the transition from the third to the second millennium BC was responsible for the continuous occupation of the Tell Mozan.

#### (d) Biomimicry

Records providing evidence for the use of FBs in biomimicry (see Table 1 for a description of the term) were scarce, restricted to Asia and focused on improving understanding of the mechanical behaviour of nacre for the design of nacre‐inspired synthetic materials (Jiao *et al*., 2019; Liu *et al*., 2020).

#### (e) Social education

Evidence for the value of FBs in social education in our database is confined to only two records, including a collaborative FB translocation initiative between the Matauranga Maori tribe (New Zealand) and western scientists, which aimed not only to renature this freshwater ecosystem but also to reconnect this indigenous tribe with their land and water (Michel *et al*., 2019).

### (2) Attitudes and other interactions

Records relate to a symbolic, spiritual or religious meaning of FBs (3%) and their importance for the cultural heritage or traditions of human communities (2%).

#### (a) Spiritual, symbolic and religious

The majority of these records relate to FB shells and pearls, mostly from the order Unionida, as burial objects used by ancient human communities in e.g. Egypt (Burleigh, 1983), Mongolia (Kiryushin *et al*., 2011), Russia (Korolev *et al*., 2018) and Argentina (Fabra, Gordillo & Piovano, 2012). More recently, from the 18th to the early 20th centuries, harvested freshwater pearls, particularly from *M. margaritifera*, have been used as decoration on various objects of religious significance (Strack, 2015).

#### (b) Tradition

Evidence on the relevance of FBs to cultural heritage and traditions in our database are restricted mainly to their use in traditional foods and particularly that of *Corbicula* spp. in traditional Asian cuisine, including soups [e.g. in China and Japan (Ke *et al*., 2011; Horikoshi, 2020)] or as smoked *etok salai* in Malaysia (Rak *et al*., 2020*a*). FBs are also considered traditional (‘first’) foods of indigenous tribes in North America, Australia and New Zealand (Brim Box *et al*., 2006; Noble *et al*., 2016).

### (3) Aesthetic experiences

Evidence for FBs providing aesthetic experiences to humans is restricted to the ornamental use of living FBs, their shells or pearls (Fig. 3B). Many of these records refer to archaeological FBs (Burleigh, 1983; Apolinarska & Kurzawska, 2020; Mărgărit, 2020). In North America and Europe, intense ‘pearl fishing’ over the past century has led to a steep decline and even extirpations of unionid populations but is now largely banned (Bauer, 1988; Anthony & Downing, 2001; Strack, 2015). Freshwater pearl culture for ornamental purposes is an important economy especially in China and other parts of Asia (Janakiram, 2003; Fiske & Shepherd, 2007), with considerable ongoing research efforts aimed at optimising the culture of high‐quality pearls and nacre (Wang *et al*., 2020). Finally, live FBs are commonly used in the ornamental pet trade (Erdoğan & Erdoğan, 2015; Ng *et al*., 2016).

### (4) Physical interactions

Evidence for FBs affecting human physical interactions is restricted to leisure and recreational activities. Most of these records refer to the effects of non‐native species, showing both their roles as providers (e.g. *D. polymorpha* increasing the recreational value of an English lake by improving water clarity; Mansfield *et al*., 2014) as well as disrupters of these ESs (e.g*. C. fluminea* shells after massive die‐off events reducing the recreational value of beaches; Fig. 3F; Ilarri *et al*., 2011).

## VI. REGULATING ECOSYSTEM SERVICES

Evidence for associations between FBs and regulating ESs (RESs) concerns the regulation of organisms, i.e. algae, bacteria and/or diseases (39% of RES records), mediation of human inputs, such as sequestration or decomposition of harmful wastes of human origin (34%), and physico‐chemical regulation, including water clearance (27%). RES records were disproportionately commonly associated with non‐native species (compare Fig. 1D with Fig. 1A–C), particularly *D. polymorpha* and *D. bugensis* in North America and Europe, and *L. fortunei* in South America. Evidence for disruption of RESs was predominantly based on non‐native rather than native species (81 *versus* 19% of records). Different taxonomic orders contributed evidence to each of the three CICES groups unequally, with Myoida and Unionida contributing particularly to biological and physico‐chemical regulation, and Veneroida and Unionida to the mediation of human inputs (Fig. 1D).

### (1) Regulation of organisms

Records relate mostly to FBs affecting the prevalence and concentration levels of algae in the water (28%; Fig. 3D) and, to a lesser extent, those of bacteria (8%) and diseases (4%), thereby affecting human health, security and/or amenity value of the respective waterbodies. Evidence for an effect on humans of regulation of organisms by FBs is available from all continents apart from Africa, and is based on both native and non‐native species.

#### (a) Algae

Evidence is exclusively based on the effects of (selective) biofiltration by FBs of phytoplankton, which is often accompanied by an effect on water clarity (Fig. 3D). Many of these studies are based on laboratory experiments that investigate the ability and rate at which FBs can ingest (and, in some studies digest) and thus, remove (certain compartments of) phytoplankton from the water column, including harmful derivatives, such as hepatotoxic microcystins and nodularins (Pham *et al*., 2016; Buelow & Waltham, 2020; Silva *et al*., 2020). A number of studies provide evidence for these mechanisms *in situ*, often through mesocosm experiments. Notable examples on the provision of this ES by native species include the alleviation of the negative effects of cyanobacterial blooms on submerged macrophyte growth by the unionid *S. cumingii* in Lake Taihu, China (He *et al*., 2014), and effective bioaccumulation of microcystins and/or nodularins by unionid species in Japanese and Latvian lakes (Park, Yokoyama & Okino, 2001; Barda *et al*., 2015). Much of the respective literature from Europe and North America is focused on non‐native, invasive *Dreissena* species, which have caused reductions and, in some cases, extirpation of local, native unionid populations (Strayer, 1999; Sousa *et al*., 2014). The prevailing evidence indicates that this shift in FB species composition results in a reduction of overall phytoplankton biomass, which is, however often accompanied by an increase in nuisance algae, such as the green alga *Cladophora glomerata* (e.g. Lake Ontario, Canada; Ozersky *et al*., 2009), and/or harmful cyanobacteria, such as *Microcystis* spp. (e.g. Laurentian Great Lakes; Vanderploeg *et al*., 2001). The negative knock‐on effects on human health have been illustrated by Jones (2019), who showed that a steep drop in microcystin levels caused by a sudden, massive die‐off of *D. polymorpha* in Gull Lake, Michigan, USA, led to improved infant health (measured as e.g. instances of low birth weight, length of gestation). That said, the effects of dreissenid invasions appear to depend on the prevailing conditions, including phosphorus concentrations, at the site (Raikow *et al*., 2004). For example, data from Lake Michigan, USA, indicate that invasion by *D. polymorpha* has led not only to an increase of cyanobacteria but also to increased overall phytoplankton density (De Stasio *et al*., 2008). On the other hand, *D. bugensis* has been shown to control phytoplankton, including cyanobacteria, in urban ponds in the Netherlands (Waajen *et al*., 2016).

#### (b) Bacteria

Scientific interest in the potential use of FBs as a bioremediation tool for bacterial contamination of water bodies has gained traction only relatively recently. One of the first studies providing strong evidence for FBs significantly reducing concentrations of bacteria of human importance is that by Bianchi *et al*. (2014), showing that the unionid *Diplodon chilensis* can reduce bacterial loads in sewage water from a Patagonian lake. *In situ* experiments by Ismail *et al*. (2015, 2016) revealed the ability of the native unionid *Anodonta californiensis* and non‐native veneroid *C. fluminea* to reduce concentrations of *Escherichia coli* in human‐impacted lakes and rivers in California, USA. Laboratory experiments in Italy provided similar results for the non‐native *D. polymorpha*, which almost completely removed *E. coli* from the water (Mezzanotte *et al*., 2016). Also in Italy, mesocosm experiments showed that integration of the non‐native unionid *S. woodiana* can reduce the environmental impacts of inland trout farming in terms of reducing total bacterial concentrations by up to 72% (Sicuro *et al*., 2020).

#### (c) Diseases

Evidence for FBs as potential bioremediation tools for human pathogenic viruses and bacteria and their vectors originated mainly from Asia and Europe. Laboratory experiments in Italy showed that non‐native *D. polymorpha* can significantly reduce concentrations of rota‐ and polioviruses in water (Mezzanotte *et al*., 2016). In Japan, the native, pearl‐producing unionid *Sinohyriopsis schlegelii* was found effectively to deplete oocysts of gastroenteritis‐inducing *Cryptosporidium parvum* in the final settling pond of a sewage plant (Izumi *et al*., 2012). A study from Indonesia showed that FB shell powder can be used as an environmentally friendly alternative to organophosphate pesticides in controlling larvae of *Anopheles* and *Aedes aegypti* mosquitos, which are vectors of malaria and dengue fever (Sorontou & Agussalim., 2016).

### (2) Mediation of human inputs

In the vast majority of records on FBs acting as remediators of harmful organic or inorganic substances of human origin, evidence relates to filtration and/or sequestration of these substances (32%), whilst evidence for (subsequent) transformation or decomposition into less harmful substances is rare (2%).

#### (a) Filtration and sequestration

Some of the earliest records are focused on the remediation of heavy metal contamination of water through FB biofiltration. This topic has received particular attention in India, usually focusing on Cadmium (Cd) and using the native unionid *L. marginalis*, which was shown to significantly alleviate Cd‐contamination in waters receiving industrial effluents (Jana & Das, 1997; Das & Jana, 2003). As FBs are also readily eaten in this region and thus can pose a health hazard, the findings of Cd‐biosorptive properties of FB shells (Ismail, Aris & Latif, 2014*a*; Hossain, Bhattacharyya & Aditya, 2015) and applied by Ghosh *et al*. (2020*b*) are particularly promising.

In Europe, North America and South America, research on the potential application of FBs as bioremediation tools for anthropogenic pollution of fresh waters is largely focused on non‐native species. In the USA, a flow‐through mussel filter designed by Diggins *et al*. (2002) using *D. polymorpha* and *D. bugensis* was found to clear up to 96% of suspended particles (to which many pollutants readily adsorb) from effluents before discharge. A similar design with ~40,000 *D. polymorpha* individuals was piloted in the largest wastewater treatment plant of Milan, Italy, resulting in the efficient removal of various heavy metals as well as pharmaceuticals and drugs of abuse (Fig. 3E; Binelli *et al*., 2014; Magni *et al*., 2015). In Portugal, *C. fluminea* was shown to be able to assist in the remediation of acid mine drainage, as well as olive oil mill and winery wastewaters (Rosa *et al*., 2014; Pipolo *et al*., 2017; Ferreira *et al*., 2018; Domingues *et al*., 2020). One of the few such records on native unionid species from outside Asia is that of *D. chilensis* alleviating environmental effects of a fish aquaculture in Chile by removing nutrients and organic matter from the water (Parada *et al*., 2008).

#### (b) Transformation

Evidence for the ability of FBs to transform substances of human origin is scarce and comes mainly from *L. fortunei*. In its native China, laboratory experiments showed that both living specimens and, to a lesser extent, shells, can reduce the concentrations of the plant growth regulator forchlorfenuron in a process involving bacteria‐associated nitrification and denitrification reactions (Zhang, Cui & Huang, 2015). In laboratory experiments in Argentina, where *L. fortunei* is invasive, herbicide (including glyphosate) and pesticide concentrations in the water decreased by 40% under live *L. fortunei* presence and by 25% in empty shell treatments, again involving transformation due to mineralisation by microbial communities (Di Fiori *et al*., 2012).

### (3) Physico‐chemical regulation

Evidence is available from all continents except South America for FBs decreasing or increasing concentrations of organic or inorganic substances (of non‐human origin) (17%) as well as affecting the physical properties of water quality (11%) with implications for human health and/or recreation.

#### (a) Storage and excretion

Particularly in China, there has been growing interest in using native unionid FBs in the restoration of freshwater ecosystems. L. Wang *et al*. (2017) showed that when combined with stocking of planktivorous fish and replanting submerged macrophytes, *S. cumingii* can effectively remove nutrients from eutrophicated waterbodies (as well as supress phytoplankton growth). Similarly, *Lamprotula leai* in combination with the annelid *Tubifex tubifex* has been demonstrated to be effective in promoting plant growth and nutrient absorption within ‘Constructed Wetlands’, an ecological engineering solution for removing and transforming pollutants from wastewater (Kang *et al*., 2018). In North America and Europe, evidence comes almost exclusively from non‐native species and predominantly *D. polymorpha* and *D. bugensis*. Research has focussed mainly on their effects on nutrient cycling (especially of phosphorus and nitrogen), which are complex and not uniform across ecosystems, rendering these invaders both providers and disruptors of this RES. In North America, a considerable body of research has been conducted on this topic in the Laurentian Great Lakes. In Lake Huron, it was shown that sequestration of phosphorus by *D. polymorpha* and *D. bugensis* led to reduced primary productivity with knock‐on effects on secondary producers and fish (Cha *et al*., 2011). In Lake Erie, modelling showed that the invasion of these FBs decreased ecosystem resistance to eutrophication, necessitating increased phosphorus management to preserve lake ESs (Roy *et al*., 2010). In Lake Simcoe, dreissenid FBs have been shown to remineralise and thus increase bioavailability of phosphorus and nitrogen, whilst their shells represent a long‐term sink for phosphorus, nitrogen and calcium (Ozersky, Evans & Ginn, 2015). On the other hand, decline in primary production and increase in water clarity caused by a dreissenid invasion has resulted in the Great Lakes becoming a significant CO_2_ net emitter (>7.7 Tg‐C annually) (Lin & Guo, 2016). Despite their disruptive effects on RESs, cultivation of *D. polymorpha* has been suggested as a tool for managing nutrient‐enriched reservoirs in the UK (McLaughlan & Aldridge, 2013) and has successfully reduced nutrient exports from the Szczecin/Oder Lagoon to the open Baltic by up to 3500 t N and 420 t P per year (Schernewski, Stybel & Neumann, 2012; Friedland *et al*., 2019).

#### (b) Water clearance

Evidence for FBs improving clarity and other physical properties of water quality with implications for humans (e.g. in terms of health or recreational activities) is mostly associated with the regulation of organisms, e.g. phytoplankton, sequestration and/or transformation of substances of human origin, and/or removal of naturally occurring substances (Holland, 1993; Schernewski *et al*., 2012; Waajen *et al*., 2016; L. Wang *et al*., 2017). Many of these studies concern non‐native dreissenids in North America and Europe, and whilst their effects on increased water clarity are generally regarded as an ES provision, they have also been shown to disrupt ESs provided by the physical properties of water, e.g. by causing unpleasant odour and taste due to increased cyanobacterial growth in the St. Lawrence River, Canada (Watson & Ridal, 2004).

## VII. DISCUSSION

### (1) Temporal and geographic trends

Our systematic review of ESs provided and disrupted by FBs has identified a number of patterns over time and space. Our data set suggests that occasional scientific interest on FB–ES associations from the late 1940s to the 1980s was followed by an exponential increase in attention. This trend coincides with increased global interest in ecology and biodiversity conservation since the 1980s (Stork & Astrin, 2014), with particular interest focussed on non‐native species since the 1990s (Vaz *et al*., 2017). The rapidly increasing rate of annual record publication on FB–ESs in the 2000s may be explained by the popularisation of the ES concept, especially following publication of the *Millennium Ecosystem Assessment* (MEA, 2005) and subsequent initiatives, such as The Economics of Ecosystems and Biodiversity, the Ecosystem Services Partnership and the Intergovernmental Science‐Policy Platform on Biodiversity and Ecosystem Services.

Despite the general increase in publications over time, the rate and amount of evidence published has not been homogenous across geographical areas. Asia stands out in our data set as the current epicentre of FB–ES research in terms of the quantity of available evidence (35% of records originate from Asia), breadth of research (23 of 24 CICES‐classes) and increase in annual evidence output in recent years. Four CICES classes were based exclusively on evidence from Asia, i.e. the use of genetic or protein information for medicinal purposes, use in the provision of energy, use in the design and production of other materials, and relevance to cultural heritage and traditions (although evidence for latter class also exists at least for North America and Australasia; Noble *et al*., 2016). Other CICES classes and groups were strongly dominated by evidence from Asia, and particularly East Asia, including the use of FBs or their products for food and material production, and the restoration of freshwater ecosystems. If anything, this dominance of Asia is likely under‐represented in our data set due to our exclusion of publications written in Asian languages. The prevalence of records on FB–ES associations from Asia may reflect the continent's size (29% of land area and 60% of global population; UN, 2021), social and biogeographical heterogeneity, and diverse FB fauna, particularly in the Indotropics (Lopes‐Lima *et al*., 2018; Zieritz *et al*., 2018*b*). However, research into other aspects of FB biology and ecology in Asia is considerably lagging behind that in North America and Europe, despite numerous conservation issues (Lopes‐Lima *et al*., 2018; Zieritz *et al*., 2018*b*).

Long‐term protection of global FB diversity and functionality will require a good understanding of both their ecology and their ESs. For example, in China, where FB products (‘commodities’), such as ornamental pearls or medicines, are particularly popular, knowledge gaps on species diversity and conservation status should be urgently addressed to avoid unsustainable exploitation of rare and/or threatened species. By contrast, in North America and Europe, ES research has so far strongly focused on the use of FBs in biomonitoring, with other potential ESs having been largely ignored. In North America particularly, significant resources have also been dedicated to generate a better understanding of the functional roles of FBs within their ecosystems (Vaughn & Hakenkamp, 2001; Vaughn, 2018), but the majority of these studies lack a clear relevance to humans. FB conservation in these regions would benefit from more explicitly recognising the contributions that FBs make to human well‐being. That said, there is evidence for this acquired knowledge on FB–ESs already pushing conservation initiatives in these regions, including the Mussels for Clean Water Initiative, which aims to restore native FB populations in the Delaware and other northwestern US river basins ‘to promote cleaner water and healthier aquatic ecosystems’ (The Partnership for the Delaware Estuary, 2017). Finally, in the southern hemisphere, all aspects of FB research, including ecology, conservation and ESs, are notoriously understudied.

Some avenues of ES research that are popular in one region may not be attractive in other regions due to differences in socio‐economic conditions and/or characteristics of the FB fauna, but efforts should be made to identify the complete range of (potential) ESs of FBs globally. For example, whilst in the western world, FBs are rarely eaten any more, their potential as a bioremediation tool for disturbed ecosystems appears to be severely underexplored. In Africa, research into the use of FBs as a source of food and biomonitoring tool may be of particular relevance (Akélé *et al*., 2015; Labuschagne *et al*., 2020). Future research would benefit greatly from international collaborations to facilitate exchange of knowledge, know‐how and technologies. Ultimately, this should be accompanied by quantification and economic valuation of ESs over space and time (Strayer, 2017), including data from unpublished and grey literature, as well as cost–benefit analyses under alternative management and environmental change scenarios.

### (2) Synergies and trade‐offs among FB ecosystem services

From our database, 146 studies provided evidence for multiple FB–ES associations, indicating that the provision/disruption of one ES coincided with the provision/disruption of one or more other ESs (synergistic and trade‐off effects; Turkelboom *et al*., 2016). Common examples for ES synergies in our database referred to knowledge acquisition (e.g. biomonitoring), regulation of organisms (e.g. algae), mediation of human inputs (e.g. heavy metals) and/or physico‐chemical regulation (e.g. through water clearance). All of these ESs, which provide benefits to human health and improved amenity value of waterbodies, derive from the considerable filtration and bioaccumulation capacity of FBs (Vaughn, 2018). The same traits are also responsible for FBs providing nutritious food (Ke *et al*., 2011; Horikoshi, 2020), and supporting the production of (shell)fish for food and the ornamental pet trade (Erdoğan & Erdoğan, 2015; Sicuro *et al*., 2020).

The effect of a particular FB on one or more ESs is often context specific, which can result in trade‐offs among the provision and disruption of specific ESs. For example, the remediation of polluted water by FB filtration may be offset by the adverse effects on human health when these FBs are consumed. Based on our data set, non‐native FBs appear to act more commonly as disrupters of ESs than native FBs, e.g. by reducing the recreational value of waterbodies by causing cyanobacterial blooms (Vanderploeg *et al*., 2001; Jones, 2019). Furthermore, invasive FBs are known to cause a range of other ecological and economic impacts, including the complete alteration of ecosystem structure and functioning, and the fouling of industrial intakes and boats (Strayer, 1999; Sousa, Gutiérrez & Aldridge, 2009; Sousa *et al*., 2014), with massive economic costs (63.8 billion US$ between 1980 and 2020 according to Haubrock *et al*., 2022). On the other hand, there is evidence for invasive FBs providing ESs, most notably through biomonitoring and nutrient removal in eutrophicated ecosystems (Richman & Somers, 2010; McLaughlan & Aldridge, 2013). Future research should aim to provide a more complete understanding and quantification of how the provisioning of one ES may reduce the provisioning of another or result in additional ecological and/or economic costs, particularly with regard to native *versus* non‐native species.

## VIII. CONCLUSIONS

(1) Our systematic review of 684 published studies identified 904 records providing evidence for an association between FBs and 24 specific ES classes. Records originated predominantly from Asia, Europe and North America, with poor representation of countries from the southern hemisphere. About one in ten records referred to a disruption rather than provision of ESs.

(2) Temporal trends in the publication of FB–ES associations reflected increased interest in ecology and conservation since the 1980s, increased attention on non‐native species since the 1990s and popularisation of the ES concept since the 2000s.

(3) Evidence on provisioning ESs, and particularly the provisioning of food, materials and medicines by *Corbicula* spp. and unionids, was prominent in Asian countries, yet this is a region where species diversity, distribution and ecology remain understudied.

(4) In North America and Europe, evidence was primarily focused on cultural ESs, and predominantly the use of native and non‐native FBs for biomonitoring. Other cultural CICES groups referred to the ornamental use of FBs, their religious, spiritual or traditional meaning, and their effects on physical interactions.

(5) Regulating ESs comprised the regulation of organisms, including (harmful) algae and bacteria, the regulation of substances and physico‐chemical water properties, and the mediation of harmful substances of human origin. Records were commonly based on non‐native species (mainly *Dreissena* spp.).

(6) Multiple FB–ES associations within single studies were often associated with the biofiltration and bioaccumulation capacity of FBs, linking various provisioning (e.g. source of micronutrients), cultural (e.g. biomonitoring) and regulating (e.g. of algae) ESs. Trade‐offs among ESs provided and disrupted by FBs were particularly commonly observed for non‐native FBs.

(7) As the global community of FB researchers grows, we recommend that future efforts are directed towards the continued documentation of FB–ESs, particularly in less well‐studied geographic regions, such as Africa and South America. Attention should be given to ESs and FBs beyond those commonly reported. Progress should be made in the economic quantification of FB–ESs, so that trade‐offs can be properly enumerated and informed decisions can be made about effective ecosystem management, conservation and restoration programs.

## Supporting information


**Appendix S1.** List of key words used in the search string performed in ISI *Web of Science* and *Scopus*.
**Appendix S2.** Classification of papers by continents according to the country(ies)/regions where the case studies took place.
**Appendix S3.** Final list of publications considered in the literature review.
