# Supplementary material for: A global synthesis of ecosystem services provided and disrupted by freshwater bivalve molluscs
Source: Biol Rev Camb Philos Soc. 2022 Jun 30;97(5):1967–98. doi: 10.1111/brv.12878 (PMC9545824; doi:10.1111/brv.12878)
Supplement: Supplementary file 1 — Appendix S1. List of key words used in the search string performed in ISI Web of Science and Scopus. Appendix S2. Classification of papers by continents according to the country(ies)/regions where the case studies took place. Appendix S3. Final list of publications considered in the literature review. [file BRV-97-1967-s001.docx]

**Appendix S1.** List of key words used in the search string performed in *ISI Web of Science* and *Scopus*.

A first search was done based on the most general key words. The search string was derived through an iterative procedure, by (1) reviewing a short list of key publications, and including pertinent key words for the search, (2) checking the records retrieved by the search, and including or excluding pre-existing and new key words, and (3) re-conducting the search with the new set of key words. New terms were added step-by-step in a participatory process among team members. The pertinence of each key word for the topic of this research was carefully discussed and analysed by the team before the start of the search. The search was performed in the field “topic” of *ISI Web of Science* and “TITLE-ABS-KEY” in *Scopus*.

| Freshwater mussels | General | "freshwater" AND (mussel*" OR "clam*" OR "bivalve*") |
| --- | --- | --- |
|  | Genera and species names | "Acostaea" OR "Actinodontophora" OR "Actinonaias" OR "Aculamprotula" OR "Acuticosta" OR "Adacna" OR "Afropisidium" OR "Alasmidonta" OR "Alathyria" OR "Amblema" OR "Americunio" OR "Amnigenia" OR "Amuranodonta" OR "Anemina" OR "Anodonta" OR "Anodontites" OR "Anodontoides" OR "Antediplodon" OR "Anthraconaia" OR "Anthracosia" OR "Anticorbula" OR "Anunio" OR "Archiparreysia" OR "Arcidens" OR "Arcidopsis" OR "Arguniella" OR "Arotonaias" OR "Aspatharia" OR "Asturianaia" OR "Baicalinaia" OR "Balwantia" OR "Bartlettia" OR "Barynaias" OR "Beringiana" OR "Bineurus" OR "Blanfordinaia" OR "Brazzaea" OR "Brussiella" OR "Buldowskia" OR "Byssanodonta" OR "Callonaia" OR "Cambarunio" OR "Carbonicola" OR "Castalia" OR "Castaliella" OR "Caudiculatus" OR "Chambardia" OR "Chamberlainia" OR "Chelidonopsis" OR "Coelatura" OR "Comptio" OR "Congeria" OR "Contradens" OR "Corbicula" OR "Costanaia" OR "Cratonaia" OR "Cristaria" OR "Ctenodesma" OR "Cucumerunio" OR "Cumberlandia" OR "Cuneopsis" OR "Cyanocyclas" OR "Cyclonaias" OR "Cyotrigonioides" OR "Cyprogenia" OR "Cyrtonaias" OR "Delphinonaias" OR "Dentaspatharia" OR "Desertella" OR "Diaurora" OR "Diplodon" OR "Diplodontites" OR "Discomya" OR "Disconaias" OR "Discunio" OR "Dreissena" OR "Dreissenomya" OR "Dromus" OR "Ebersininaia" OR "Echyridella" OR "Ellipsaria" OR "Elliptio" OR "Elliptoideus" OR "Elongaria" OR "Ensidens" OR "Eocuneopsis" OR "Eolamprotula" OR "Eonaias" OR "Eonippononaia" OR "Epioblasma" OR "Etheria" OR "Euglesa" OR "Eupera" OR "Eurynia" OR "Ferganoconcha" OR "Fossula" OR "Friersonia" OR "Fusconaia" OR "Gautieraia" OR "Germainaia" OR "Gibbosula" OR "Glebula" OR "Gonidea" OR "Grafunio" OR "Grandidieria" OR "Haasica" OR "Haasodonta" OR "Hadrodon" OR "Hamiota" OR "Harmandia" OR "Hemistena" OR "Hoffetrigonia" OR "Hyridella" OR "Hyriopsis" OR "Iheringella" OR "Indobaphia" OR "Indochinella" OR "Indonaia" OR "Inversidens" OR "Inversiunio" OR "Itatia" OR "Koreanaia" OR "Koreosolenaia" OR "Lamellidens" OR "Lamproscapha" OR "Lamprotula" OR "Lampsilis" OR "Lanceolaria" OR "Lasmigona" OR "Leaunio" OR "Leguminaia" OR "Leila" OR "Lemiox" OR "Leoparreysia" OR "Lepidodesma" OR "Leptanodonta" OR "Leptodea" OR "Ligumia" OR "Lortiella" OR "Margaritifera" OR "Martensnaias" OR "Martinsoniconcha" OR "Medionidus" OR "Megalonaias" OR "Megalovirgus" OR "Mengyinaia" OR "Mesohyridella" OR "Microcondylaea" OR "Microdonta" OR "Microdontia" OR "Micronaias" OR "Middendorffinaia" OR "Modellnaia" OR "Moncetia" OR "Monginella" OR "Monocondylaea" OR "Monodontina" OR "Mujanaia" OR "Mutela" OR "Mycetopoda" OR "Mycetopodella" OR "Naiadites" OR "Nakamuranaia" OR "Neamnigenia" OR "Nephritica" OR "Nephronaias" OR "Nippononaia" OR "Nitia" OR "Nkondonaia" OR "Nodularia" OR "Novaculina" OR "Nyassunio" OR "Nyeinchanconcha" OR "Obliquaria" OR "Obovalis" OR "Obovaria" OR "Obwerukunio" OR "Odhneripisidium" OR "Ortmanniana" OR "Pachynaias" OR "Paetulunio" OR "Palindonaia" OR "Paranodonta" OR "Parreysia" OR "Parvasolenaia" OR "Parvaspina" OR "Pegias" OR "Peregrinoconcha" OR "Physunio" OR "Pilsbryoconcha" OR "Pisidium" OR "Plectomerus" OR "Pledgia" OR "Pleiodon" OR "Plesielliptio" OR "Plethobasus" OR "Pletholophus" OR "Pleurobema" OR "Pleuronaia" OR "Plicatounio" OR "Pliconaias" OR "Popenaias" OR "Potamilus" OR "Potomida" OR "Pressidens" OR "Prisodon" OR "Prisodontopsis" OR "Proarcidopsis" OR "Prodreissensia" OR "Prohyria" OR "Prohyriopsis" OR "Pronodularia" OR "Proparreysia" OR "Propotomida" OR "Protamblema" OR "Protelliptio" OR "Protopleurobema" OR "Protunio" OR "Pseudanodonta" OR "Pseudobaphia" OR "Pseudobovaria" OR "Pseudocardinia" OR "Pseudodiplodon" OR "Pseudodon" OR "Pseudodontoideus" OR "Pseudodontopsis" OR "Pseudohyria" OR "Pseudomulleria" OR "Pseudospatha" OR "Pseudunio" OR "Psilounio" OR "Psoronaias" OR "Ptychobranchus" OR "Ptychorhynchoideus" OR "Ptychorhynchus" OR "Pyganodon" OR "Qiyangia" OR "Quadrula" OR "Radiatula" OR "Rectidens" OR "Reginaia" OR "Reticulatus" OR "Rhabdotophorus" OR "Rheodreissena" OR "Rhombuniopsis" OR "Rugunio" OR "Sagittunio" OR "Saharella" OR "Scabiellus" OR "Scabies" OR "Scaphula" OR "Schepmania" OR "Schistodesmus" OR "Sibirinaia" OR "Silesunio" OR "Simpsonaias" OR "Simpsonella" OR "Sinanodonta" OR "Sinohyriopsis" OR "Sinomytilus" OR "Sinonaia" OR "Sinucongeria" OR "Sinzowinaia" OR "Solenaia" OR "Sphaerium" OR "Sphenonaias" OR "Strophitus" OR "Sulcatapex" OR "Sulcatula" OR "Sulcopotomida" OR "Sundadontina" OR "Tamesnella" OR "Tamsiella" OR "Tanysiphon" OR "Tchulymiconcha" OR "Teruella" OR "Thaiconcha" OR "Theliderma" OR "Tihkia" OR "Toxolasma" OR "Trapezidens" OR "Trapezoideus" OR "Trigonioides" OR "Triplodon" OR "Tritogonia" OR "Truncilla" OR "Unio" OR "Uniomerus" OR "Unionelloides" OR "Unionetta" OR "Utterbackia" OR "Utterbackiana" OR "Velesunio" OR "Venustaconcha" OR "Vetulonaia" OR "Villosa" OR "Virgus" OR "Westralunio" OR "Yaukthwa" OR "Arcuatula arcuatula" OR "Iphigenia laevigata" OR "*Limnoperna fortunei*" OR "Mytilopsis leucophaeata" OR "Mytilopsis sallei" OR "Mytilopsis trautwineana" OR "Monodacna colorata" OR "Pharella waltoni" OR "Profischeria centralis") |
| Ecosystem services | General | "ecosystem service*" OR "environment* service*" OR "ecologic* service*" OR "ecosystem function*" OR "environment* function*" OR "ecologic* function*" OR "Value*" OR "Valuation" OR "Valuing" OR "Price*" OR "Pricing" |
|  | Provisioning services | "Food" OR "Nutrition" OR "medicine*" OR "remedy" OR "pharmacy" OR "Nacre*" OR "Pearl hunting" OR "Button*" OR "Aquaculture" OR "fishing" OR "bait" OR "fiddle*" OR "violin*" OR "knife*" OR "bead*" |
|  | Regulating services | "*remediation" OR "waste mediation" OR "water filtration" OR "bioaccumulation" OR "Chemical deposition" OR "Biomonitor*" OR "Environmental monitoring" OR "Biosentinel*" OR "Valvometry" OR "bacteria* removal" OR "pest control" OR "disease* control" OR "disease* removal" OR "Water pollution removal" OR "water quality" OR "water purification" OR "Sediment control" OR "Sediment regulation" OR "Sediment propert*" OR "Sediment quality" OR "ecosystem engineer*" OR "Sediment maintenance" OR "Sediment stabilization" OR "Sediment stabilisation" OR "Sediment rate" OR "Sedimentation" OR "Bioirrigation" OR "Bioturbation" OR "Biodeposit*" OR "Resuspension" OR "pseudofaeces" OR "Genetic diversity" OR "genetic variety" OR "gene pool" OR "gene flow" OR "Genetic differentiation" OR "Genetic bottleneck" OR "carbon sequestration" OR "carbon storage" OR "climate regulation" OR "carbon uptake" OR "carbon removal" OR "CO2 sequestration" OR "CO2 storage" OR "CO2 regulation" OR "CO2 removal" OR "*nitrification" OR "N*removal" OR "P*removal" OR "Nutrient retention" OR "N*retention" OR "P*retention" OR "C*retention" OR "Nutrient cycling" OR "Nutrient recycling" OR "Nutrient storage" OR "Nutrient reduction" OR "Nutrient translocation" OR "Nutrient capacitor*" OR "Nutrient sink*" OR "Nutrient retention" OR "Phosphorus cycling" OR "Calcification" OR "Sclerochronology" OR "*filtration" OR "Filter*feeders" OR "Clearance rate*" |
|  | Cultural services | "art" OR "ornament" OR “recreation*" OR "*tourism" OR "aesthetic*" OR "inspiration*" OR "traditional knowledge" OR "local knowledge" OR "education" OR "training" OR "cultural heritage" OR "sacred" OR "religious" OR "symbol*" OR "entertainment" OR "shell collection" OR "Amenity value" OR "Pet trade" OR "Aquarium" OR "palaeontology" OR "archaeology" |

**Appendix S2.** Classification of papers by continents according to the country(ies)/regions where the case studies took place.

| **Africa** |
| --- |
| Benin |
| Egypt |
| Kenya |
| Morocco |
| Niger |
| South Africa |
| Tanzania |
| Tunisia |
| **Asia** |
| Bangladesh |
| Cambodia |
| China |
| Eastern Black Sea |
| India |
| Indonesia |
| Iran |
| Iraq |
| Japan |
| Malaysia |
| Mongolia |
| Oman |
| Pakistan |
| Philippines |
| Russia |
| Saudi Arabia |
| South Korea |
| Syria |
| Taiwan |
| Thailand |
| Turkey |
| Vietnam |
| **Australasia** |
| Australia |
| New Zealand |
| **Europe** |
| Austria |
| Belarus |
| Belgium |
| Bulgaria |
| Croatia |
| Denmark |
| Finland |
| France |
| Greece |
| Germany |
| Hungary |
| Ireland |
| Italy |
| Latvia |
| Lithuania |
| Luxembourg |
| Macedonia |
| Poland |
| Portugal |
| Romania |
| Serbia |
| Portugal |
| Sweden |
| Switzerland |
| The Netherlands |
| UK |
| Ukraine |
| **North America** |
| Canada |
| USA |
| **Pacific Ocean** |
| Tahiti |
| **South America** |
| Argentina |
| Brazil |
| Chile |
| Colombia |
| Equador |
| Peru |

**Appendix S3.** Final list of publications considered in the literature review.

References identified with an asterisk (*) are used only in the supporting information.

1. *Abaychi, J. K. & Mustafa, Y. Z. (1988). The asiatic clam, *Corbicula fluminea*: An indicator of trace metal pollution in the Shatt al-Arab River, Iraq. *Environmental Pollution* **54**, 109–122.
2. *Adams, S. M. & Shorey, C. D. (1998). Energy dispersive spectroscopy of granular concretions in the mantle of the freshwater mussel *Hyridella depressa* from Lake Burragorang as a technique to monitor metals in aquatic systems. *Aquatic Toxicology* **44**, 93­102.
3. *Aguirre-Martínez, G. V., DelValls, A. T. & Laura Martín-Díaz, M. (2015). Yes, caffeine, ibuprofen, carbamazepine, novobiocin and tamoxifen have an effect on *Corbicula fluminea* (Müller, 1774). *Ecotoxicology and Environmental Safety* **120**, 142–154.
4. *Ahmed, M. K., Bhowmik, A. C., Rahman, S. & Haque, M. R. (2010). Heavy metal concentration in water, sediments, freshwater mussels and fishes of the River Shitalakhya, Bangladesh. *Asian Journal of Water Environment and Pollution* **7**, 77–90.
5. *Akarte, S. R. & Bahadure, R. B. (2015). Evaluation of minerals as manure value of faecal matter of chicks fed with molluscan supplementary diet International. *Journal of Pharma and Bio Sciences* **6**, B1342–B1346.
6. Akélé, G., Agadjihouèdé, H., Mensah, G. & Lalèyè, P. (2015). Population dynamics of freshwater oyster *Etheria elliptica* (Bivalvia: Etheriidae) in the Pendjari River (Benin-Western Africa). *Knowledge and Management of Aquatic Ecosystems* **416**, 06.
7. *Aksoy, A., Das, Y. K., Yavuz, O., Guvenc, D., Atmaca, E. & Agaoglu, S. (2011). Organochlorine pesticide and polychlorinated biphenyls levels in fish and mussel in Van Region, Turkey. *Bulletin of Environmental Contamination and Toxicology* **87**, 65–69.
8. *Aksu, O., Yabanli, M., Can, E., Kutluyer, F., Kehayias, G., Can, S.S., Kocabaş, M. & Demir, V. (2012). Comparison of heavy metals bioaccumulation by *Dreissena polymorpha* (Pallas, 1771) and *Unio elongatulus eucirrus* (Bourguignat, 1860) from Keban Dam Lake, Turkey. *Fresenius Environmental Bulletin* **21**, 1942–1947.
9. *Al-Aasm, I. S., Clarke, J. D., Fryer, B. J. (1998). Stable isotopes and heavy metal distribution in *Dreissena polymorpha* (Zebra Mussels) from western basin of Lake Erie, Canada. *Environmental Geology* **33**, 122–129.
10. *Al-Masri, M. S., Byrakdar, M. E., Mamish, S. & Al-Haleem, M. A. (2004). Determination of natural radioactivity in Euphrates river. *Journal of Radioanalytical and Nuclear Chemistry* **261**, 349–355.
11. *Al-Mudaffar, N., Fawzi, I. N. O. & Al-Edanee, T. (1990). Hydrocarbons in surface sediments and bivalves from Shatt Al-Arab and its rivers, Southern Iraq. *Oil and Chemical Pollution* **7**, 17–28.
12. *Al-Taher, Q. M., Akbar, M. M. & Al-Qarooni, I. H. (2020). Estimation of heavy metals in water, sediments and bioaccumulation in two species of Mollusca: Clam *Pseudodontopsis euphraticus* and snail *Bellamya bengalensis* in Euphrates River in Al-Nasiriyah City, south of Iraq. *Plant Archives* **20**, 1454–1460.
13. *Alcaraz, C., Caiola, N. & Ibáñez, C. (2011). Bioaccumulation of pollutants in the zebra mussel from hazardous industrial waste and evaluation of spatial distribution using GAMs. *Science of the Total Environment* **409**, 898–904.
14. *Aldridge, D. C. & Horne, D. C. (1998). Fossil glochidia (Bivalvia, Unionidae): Identification and value in palaeoenvironmental reconstructions. *Journal of Micropalaeontolology* **17**, 179–182.
15. Aldridge, D. C., Salazar, M., Serna, A. & Cock, J. (2008). Density-dependent effects of a new invasive false mussel, *Mytilopsis trautwineana* (Tryon 1866), on shrimp, *Litopenaeus vannamei* (Boone 1931), aquaculture in Colombia. *Aquaculture* **281**, 34–42.
16. *Algan, O., Çaǧatay, N., Tchepalyga, A., Ongan, D., Eastoe, C. & Gökaşan, E. (2001). Stratigraphy of the sediment infill in Bosphorus Strait: Water exchange between the Black and Mediterranean Seas during the last glacial holocene. *Geo-Marine Letters* **20**, 209–218.
17. Alif, M. F., Aprillia, W. & Arief, S. (2018). Peat water purification by hydroxyapatite (HAp) synthesized from waste pensi (*Corbicula moltkiana*) shells. *IOP Conference Series: Materials Science and Engineering* **299**, 01(2002).
18. *Allen, H. J., Dickson, K. L., Martin, H., Thuesen, K. A. & Waller, W. T. (2002). Monitoring watersheds: Biomonitors and other measures. *Journal of Urban Technology* **9**, 1–19.
19. *Amraoui, I., Khalloufi, N. & Touaylia, S. (2018). Effects to perfluorooctane sulfonate (PFOS) on the mollusk *Unio ravoisieri* under laboratory exposure. *Chemistry and Ecology* **34**, 324–339.
20. *Andrès, S., Baudrimont, M., Lapaquellerie, Y., Ribeyre, F., Maillet, N., Latouche, C. & Boudou, A. (1999). Field transplantation of the freshwater bivalve *Corbicula fluminea* along a polymetallic contamination gradient (River Lot, France): I. Geochemical characteristics of the sampling sites and cadmium and zinc bioaccumulation kinetics. *Environmental Toxicology and Chemistry* **18**, 2462–2471.
21. Andrews, J. E. & Walton, W. (1990). Depositional environments within Middle Jurassic oyster-dominated lagoons: an integrated litho-, bio- and palynofacies study of the Duntulm Formation (Great Estuarine Group, Inner Hebrides). *Transactions of the Royal Society of Edinburgh: Earth Sciences* **81**, 1–22.
22. *Antunes, F., Hinzmann, M., Lopes-Lima, M., Machado, J. & da Costa, P.M. (2010). Association between environmental microbiota and indigenous bacteria found in hemolymph, extrapallial fluid and mucus of *Anodonta cygnea* (Linnaeus, 1758). *Microbial Ecology* **60**, 304–309.
23. *Anzano, J., Lasheras, R.-J., Bonilla, B., Bonilla, A., Lanaja, J., Peribañez, M. A., Gracia-Salinas, M.-J., Anwar, J. & Shafique, U. (2011). Determination of trace metals by voltamperometry in zebra mussel (*Dreissena polymorpha*) employed as environmental bio-indicator. *Green Chemistry Letters and Reviews* **4**, 261–267.
24. *Apolinarska, K. (2009). delta O-18 and delta C-13 isotope investigation of the Late Glacial and early Holocene biogenic carbonates from the Lake Lednica sediments, western Poland. *Acta Geologica Polonica* **59**, 111–121.
25. *Apolinarska, K. (2013). Stable isotope compositions of recent *Dreissena polymorpha* (Pallas) shells: Paleoenvironmental implications. *Journal of Paleolimnology* **50**, 353–364.
26. *Apolinarska, K. & Hammarlund, D. (2009). Multi-component stable isotope records from Late Weichselian and early Holocene lake sediments at Imiołki, Poland: Palaeoclimatic and methodological implications. *Journal of Quaternary Science* **24**, 948–959.
27. Apolinarska, K. & Kurzawska, A. (2020). Can stable isotopes of carbon and oxygen be used to determine the origin of freshwater shells used in Neolithic ornaments from Central Europe? *Archaeological and Anthropological Sciences* **12**, 15.
28. *Areekijseree, M., Engkagul, A., Kovitvadhi, U., Thongpan, A., Mingmuang, M., Pakkong, P. & Rungruangsak-Torrissen K. (2004). Temperature and pH characteristics of amylase and proteinase of adult freshwater pearl mussel, *Hyriopsis* (*Hyriopsis*) *bialatus* Simpson 1900. *Aquaculture* **234**, 575–587.
29. *Arfiati, D., Putra, C. D. G., Tullah, A. H., Permanasari, S. W. A. & Puspitasari, A. W. (2019). The dynamics of total organic matter (tom) on sangkuriang catfish (*Clarias gariepinus*) farming at upt ptpbp2kp and the effectiveness of freshwater bivalve (*Anodonta woodiana*) in reducing the total organic matter with varying density. *IOP Conference Series: Earth and Environmental Science* **236**, 012022.
30. *Arini, A., Baudrimont, M., Feurtet-Mazel, A., Coynel, A., Blanc, G., Coste, M. & Delmas F. (2011). Comparison of periphytic biofilm and filter-feeding bivalve metal bioaccumulation (Cd and Zn) to monitor hydrosystem restoration after industrial remediation: A year of biomonitoring. *Journal of Environmental Monitoring* **13**, 3386–3398.
31. *Arini, A., Daffe, G., Gonzalez, P., Feurtet-Mazel, A. & Baudrimont M. (2014). What are the outcomes of an industrial remediation on a metal-impacted hydrosystem? A 2-year field biomonitoring of the filter-feeding bivalve *Corbicula fluminea*. *Chemosphere* **108**, 214–224.
32. *Arribere, M. A., Campbell, L. M., Rizzo, A. P., Arcagni, M., Revenga, J. & Guevara, S.R. (2010). Trace elements in plankton, benthic organisms, and forage fish of Lake Moreno, Northern Patagonia, Argentina. *Water Air and Soil Pollution* **212**, 167–182.
33. *Arumugam, A., Li, J., Krishnamurthy. P., Jia. Z. X., Leng, Z., Ramasamy, N. & Du, D. (2020). Investigation of toxic elements in *Carassius gibelio* and *Sinanodonta woodiana* and its health risk to humans. *Environmental Science and Pollution Research* **27**, 19955–19969.
34. *Asokan, R. & Hameed, P. S. (1992). Distribution of natural radionuclide40K in biotic and abiotic components of the Cauvery river system, Tiruchirapalli, India. *Journal of Biosciences* **17**, 491–497.
35. *Atkinson, C. L., Christian, A. D., Spooner, D. E. & Vaughn, C. C. (2014). Long-lived organisms provide an integrative footprint of agricultural land use. *Ecological Applications* **24**, 375–384.
36. *Babar, A. G., Jayawant, M. S. & Pawar, S. P. (2017). Nutritional profile of the freshwater edible Bivalve *Lamellidens corrianus* (Lea 1834) and its relation to water quality in the Bhatsa River, India. *Asian Fisheries Science* **30**, 52–69.
37. *Bacchetta, R. & Mantecca, P. (2009) DDT polluted meltwater affects reproduction in the mussel *Dreissena polymorpha*. *Chemosphere* **76**, 1380–1385.
38. *Bahtiar, Anadi, L., Nurgayah, W. & dan Emiyarti (2018). Population dynamics of Pokea Clam *Batissa violacea* var. *celebensis* von Martens 1897 at Lasolo Estuary of Southeast Sulawesi. *Jurnal Ilmudan Teknologi Kelautan Tropis* **10**, 301–315.
39. Bai, J., Chen, Y., Ning, Z., Liu, S., Xu, C. & Yan, J.-K. (2020). Proteoglycan isolated from *Corbicula fluminea* exerts hepato-protective effects against alcohol-induced liver injury in mice. *International Journal of Biological Macromolecules* **142**, 1–10.
40. Bai, X. & Acharya, K. (2019). Uptake of endocrine-disrupting chemicals by quagga mussels (*Dreissena bugensis*) in an urban-impacted aquatic ecosystem. *Environmental Science and Pollution Research* **26**, 250–258.
41. *Bai, Z., Li, Q., Han, X. & Li, J. (2017). Estimates of genetic parameters and genotype by environment interactions for shell nacre color and growth traits in the purple freshwater pearl mussel *Hyriopsis cumingii*. *Aquaculture International* **25**, 2079–2090.
42. *Baldwin, A. K., Spanjer, A. R., Rosen, M. R. & Thom, T. (2020). Microplastics in Lake Mead National Recreation Area, USA: Occurrence and biological uptake. *PLoS ONE* **15**, e0228896.
43. *Baqar, M., Sadef, Y., Ahmad, S. R., Mahmood, A., Li, J. & Zhang, G. (2018). Organochlorine contaminants in freshwater mussels; occurrence, bioaccumulation pattern, spatio-temporal distribution and human health risk assessment from the tributaries of River Ravi, Pakistan. *Human and Ecological Risk Assessment* **24**, 1268–1290.
44. *Bar-Yosef Mayer, D. E., Leng, M. J., Aldridge, D. C., Arrowsmith, C., Gümüş, B.A. & Sloane, H. J. (2012). Modern and early-middle Holocene shells of the freshwater mollusc *Unio*, from Çatalhöyük in the Konya Basin, Turkey: Preliminary palaeoclimatic implications from molluscan isotope data. *Journal of Archaeological Science* **39**, 76–83.
45. Barda, I., Kankaanpää, H., Purina, I., Balode, M., Sjövall, O. & Meriluoto, J. (2015). Bioaccumulation of hepatotoxins–A considerable risk in the Latvian environment. *Environmental Pollution* **196**, 313–320.
46. Baroni, C., Bruschi, G., Veronese, L. & Zanchetta, G. (2001). Younger Dryas to Early Holocene palaeoenvironmental evolution of the Lake Terlago (Southern Alps, Italy). *Supplementi di Geografia Fisica e Dinamica Quaternaria* **24**, 13–24.
47. *Basack, S. B., Oneto, M. L., Fuchs, J. S., Wood, E. J. & Kesten, E. M. (1998). Esterases of *Corbicula fluminea* as biomarkers of exposure to organophosphorus pesticides. *Bulletin of Environmental Contamination and Toxicology* **61**, 569–576.
48. Bayerle, D. F., Nunes, R. V., Junior, A. C. G., Wachholz, L., Scherer, C., da Silva, I. M., de Oliveira-Bruxel, T. M. & de Vargas Junior, J. G. (2017). Golden mussel (*Limnoperna* *fortunei*) in feed for broiler chicks using tannin as a sequestrant of toxic metals. *Semina: Ciências Agrárias* **38**, 843–854.
49. *Bayerle, D. F., Nunes, R. V., Wachholz, L., De Oliveira Bruxel, T. M., De Vargas, J. G., Jr., Sangalli, G., Giron, T. V. & Schone, R. A. (2019). Use of golden mussel and wattle tannin in the supply of cut chickens. *Semina:Ciencias Agrarias* **40**, 1951–1964.
50. Beasley, C. R. (2001). The impact of exploitation on freshwater mussels (Bivalvia: Hyriidae) in the Tocantins River, Brazil. *Studies on Neotropical Fauna and Environment* **36**, 159–165.
51. *Beaver, J. R., Crisman, T. L. & Brock, R. J. (1991). Grazing effects of an exotic bivalve (*Corbicula fluminea*) on hypereutrophic lake water. *Lake and Reservoir Management* **7**, 45–51.
52. *Becker-van Slooten, K. & Tarradellas, J. (1995). Organotins in Swiss lakes after their ban: Assessment of water, sediment, and *Dreissena polymorpha* contamination over a four-year period. *Archives of* *Environmental Contamination and Toxicology* **29**, 384–392.
53. *Belanger, S. E., Cherry, D. S. & Cairns, Jr. J. (1986). Seasonal, behavioral and growth changes of juvenile *Corbicula fluminea* exposed to chrysotile asbestos. *Water Research* **20**, 1243–1250.
54. *Belanger, S. E., Cherry, D. S., Cairns, Jr. J. & McGuire, M. J. (1987). Using Asiatic clams as a biomonitor for chrysotile asbestos in public water supplies. *Journal American Water Works Association* **79**, 69–74.
55. *Benito, M., Mosteo, R., Rubio, E., LaPlante, D., Ormad, M.P. & Goñi, P. (2017). Bioaccumulation of inorganic elements in *Dreissena polymorpha* from the Ebro River, Spain: could Zebra Mussels be used as a bioindicator of the impact of human activities? *River Research and Applications* **33**, 718–728.
56. *Bennet-Chambers, M., Davies, P. & Knott, B. (1999). Cadmium in aquatic ecosystems in Western Australia: A legacy of nutrient-deficient soils. *Journal of Environmental Management* **57**, 283–295.
57. *Berny, Ph., Lachaux, O., Buronfosse, T., Mazallon, M. & Gillet, C. (2002). Zebra mussels (*Dreissena polymorpha*) as indicators of freshwater contamination with lindane. *Environmental Research* **90**, 142–151.
58. *Bertin, L. (2015). Exploitation of mother of pearl in the Middle Ages, Clos d'Ugnac archaeological site (Pennautier, Aude, France): Malacological study, consumption, exploitation and utilization of the nacre. *Quaternary International* **375**, 145–152.
59. *Bervoets, L., Voets, J., Chu, S., Covaci, A., Schepens, P. & Blust, R. (2004). Comparison of accumulation of micropollutants between indigenous and transplanted zebra mussels (*Dreissena polymorpha*). *Environmental Toxicology and Chemistry* **23**, 1973–1983.
60. *Bervoets, L., Voets, J., Covaci, A., Chu, S., Qadah, D., Smolders, R., Schepens, P. & Blust, R. (2005*a*). Use of transplanted zebra mussels (*Dreissena polymorpha*) to assess the bioavailability of microcontaminants in flemish surface waters. *Environmental Science and Technology* **39**, 1492–1505.
61. *Bervoets, L., Voets, J., Smolders, R. & Blust, R. (2005*b*). Metal accumulation and condition of transplanted zebra mussel (*Dreissena polymorpha*) in metal polluted rivers. *Aquatic Ecosystem Health and Management* 8, 451–460.
62. *Bettinetti, R., Quadroni, S., Galassi, S., Bacchetta, R., Bonardi, L. & Vailati, G. (2008). Is meltwater from Alpine glaciers a secondary DDT source for lakes? *Chemosphere* **73**, 1027–1031.
63. *Bhakta, J. N. & Munekage, Y. (2008). Role of ecosystem components in Cd removal process of aquatic ecosystem. *Ecological Engineering* **32** 274 280
64. *Bhalchandra, W. & Rahane, B. 2015 Biomonitoring of heavy metals (Cadmium, zinc, copper, and lead) from Gangapur reservoir using three freshwater bivalve species. *Pollution Research* **34**, 125–132.
65. *Bian, X., Liu, H., Gan, J., Li, R. & Yang, J. (2009) HCH and DDT residues in bivalves *Anodonta woodiana* from the Taihu Lake, China. *Archives of Environmental Contamination and Toxicology* **56**, 67–76.
66. Bianchi, V. A., Castro, J. M., Rocchetta, I., Bieczynski, F. & Luquet, C. M. (2014). Health status and bioremediation capacity of wild freshwater mussels (*Diplodon chilensis*) exposed to sewage water pollution in a glacial Patagonian lake. *Fish & Shellfish Immunology* **37**, 268–277.
67. *Bighiu, M. A., Norman Haldén, A., Goedkoop, W. & Ottoson, J. (2019). Assessing microbial contamination and antibiotic resistant bacteria using zebra mussels (*Dreissena polymorpha*). *Science of the Total Environment* **650**, 2141–2149.
68. *Bigot, A, Vasseur, P & Rodius, F (2010). SOD and CAT cDNA cloning, and expression pattern of detoxification genes in the freshwater bivalve *Unio tumidus* transplanted into the Moselle river. *Ecotoxicology* **19**, 369–376.
69. *Bilos, C., Colombo, J. C. & Presa, M. J. R. (1998). Trace metals in suspended particles, sediments and Asiatic clams (*Corbicula fluminea*) of the Rio de la Plata estuary, Argentina. *Environmental Pollution* **99**, 1–11.
70. *Binelli, A., Bacchetta, R., Vailati, G., Galassi, S. & Provini, A. (2001*a*). DDT contamination in Lake Maggiore (N. Italy) and effects on zebra mussel spawning. *Chemosphere* **45**, 409–415.
71. *Binelli, A., Guzzella, L. & Roscioli, C. (2008). Levels and congener profiles of polybrominated diphenyl ethers (PBDEs) in Zebra mussels (*D. polymorpha*) from Lake Maggiore (Italy). *Environmental Pollution* **153**, 610–617.
72. *Binelli, A., Magni, S., Della Torre, C. & Parolini, M. (2015). Toxicity decrease in urban wastewaters treated by a new biofiltration process. *Science of the Total Environment* **537**, 235–242.
73. Binelli, A., Magni, S., Soave, C., Marazzi, F., Zuccato, E., Castiglioni, S., Parolini, M. & Mezzanotte, V. (2014). The biofiltration process by the bivalve *D. polymorpha* for the removal of some pharmaceuticals and drugs of abuse from civil wastewaters. *Ecological Engineering* **71**, 710–721.
74. *Binelli, A. & Provini, A. (2003). DDT is still a problem in developed countries: The heavy pollution of Lake Maggiore. *Chemosphere* **52**, 717–723.
75. *Binelli, A. & Ricciardi, F. & Provini, A. (2004). Present status of POP contamination in Lake Maggiore (Italy). *Chemosphere* **57**, 27–34.
76. Binelli, A., Ricciardi, F., Riva, C. & Provini, A. (2006). Integrated use of biomarkers and bioaccumulation data in Zebra mussel (*Dreissena polymorpha*) for site-specific quality assessment. *Biomarkers* **11**, 428–448.
77. *Binelli, A., Galassi, S. & Provini, A. (2001*b*). Factors affecting the use of *Dreissena polymorpha* as a bioindicator: The PCB pollution in Lake Como (N. Italy). *Water Air and Soil Pollution* **125**, 19–32.
78. Binkowski, Ł. J., Błaszczyk, M., Przystupińska, A., Ożgo, M. & Massanyi, P. (2019). Metal concentrations in archaeological and contemporary mussel shells (Unionidae): Reconstruction of past environmental conditions and the present state. *Chemosphere* **228**, 756–761.
79. Black, B. A., Dunham, J. B., Blundon, B. W., Raggon, M. F. & Zima, D. (2010). Spatial variability in growth-increment chronologies of long-lived freshwater mussels: implications for climate impacts and reconstructions. *Ecoscience* **17**, 240–250.
80. *Blackwell, B. D., Driscoll, C. T., Spada, M. E., Todorova, S. G. & Montesdeoca, M. R. (2013). Evaluation of zebra mussels (*Dreissena polymorpha*) as biomonitors of mercury contamination in aquatic ecosystems. *Environmental Toxicology and Chemistry* **32**, 638–643.
81. *Błazejowski, B., Racki, G., Gieszcz, P., Małkowski, K., Kin, A. & Krzywiecka, K. (2013). Comparative oxygen and carbon isotopic records of miocene and recent lacustrine unionid bivalves from Poland. *Geological Quarterly* **57**, 113–122.
82. *Bodis, E., Toth, B. & Sousa, R. (2014). Massive mortality of invasive bivalves as a potential resource subsidy for the adjacent terrestrial food web. *Hydrobiologia* **735**, 253–262.
83. *Boegman, L., Loewen, M. R., Culver, D. A., Hamblin, P. F. & Charlton, M. N. (2008). Spatial-dynamic modeling of algal biomass in lake erie: Relative impacts of dreissenid mussels and nutrient loads. *Journal of Environmental Engineering* **134**, 456–468.
84. Bollhöfer, A. (2012). Stable lead isotope ratios and metals in freshwater mussels from a uranium mining environment in Australia’s wet-dry tropics. *Applied Geochemistry* **27**, 171–185.
85. *Bolognesi, C., Buschini, A., Branchi, E., Carboni, P., Furlini, M., Martino, A., Monteverde, M., Poli, P. & Rossi, C. (2004). Comet and micronucleus assays in zebra mussel cells for genotoxicity assessment of surface drinking water treated with three different disinfectants. *Science of the Total Environment* **333**, 127–136.
86. *Boltovskoy, D., Correa, N., Cataldo, D., Stripeikis, J. & Tudino, M. (1997). Environmental stress on *Corbicula fluminea* (Bivalvia) in the Parana River delta (Argentina): Complex pollution-related disruption of population structures. *Archiv fur Hydrobiologie* **138**, 483–507.
87. *Bonnail, E., Buruaem, L. M., Araujo, G. S., Abessa, D. M. S. & & DelValls, T. Á. (2016). Multiple biomarker responses in *Corbicula fluminea* exposed to copper in laboratory toxicity tests. *Archives of Environmental Contamination and Toxicology* **71**, 278–285.
88. Bonnail, E., Buruaem, L. M., Morais, L. G., Araujo, G. S., Abessa, D. M. S., Sarmiento, A. M. & DelValls, T. Á. (2018). Integrative assessment of sediment quality in lower basin affected by former mining in Brazil. *Environmental Geochemistry and Health* **40**, 1465–1480.
89. Bonnail, E., Macías, F. & Osta, V. (2019*a*). Ecological improvement assessment of a passive remediation technology for acid mine drainage: Water quality biomonitoring using bivalves. *Chemosphere* **219**, 695–703.
90. *Bonnail, E., Riba, I., de Seabra, A. A. & DelValls, T. Á. (2019*b*). Sediment quality assessment in the Guadalquivir River (SW, Spain) using caged Asian clams: A biomarker field approach. *Science of the Total Environment* **650**, 1996–2003.
91. *Bontes, B. M., Verschoor, A. M., Dionisio Pires, L. M., Van Donk, E. & Ibelings, B. W. (2007). Functional response of *Anodonta anatina* feeding on a green alga and four strains of cyanobacteria, differing in shape, size and toxicity. *Hydrobiologia* **584**, 191–204.
92. *Borcherding, J. & Jantz, B. (1997). Valve movement response of the mussel *Dreissena* *polymorpha* - The influence of pH and turbidity on the acute toxicity of pentachlorophenol under laboratory and field conditions. *Ecotoxicology* **6**, 153–165.
93. *Borcherding, J. & Wolf, J. (2001). The influence of suspended particles on the acute toxicity of 2-chloro-4-nitro-aniline, cadmium, and pentachlorophenol on the valve movement response of the zebra mussel (*Dreissena polymorpha*). *Archives of Environmental Contamination and Toxicology* **40**, 497–504.
94. *Borković-Mitić, S., Pavlović, S., Perendija, B., Despotović, S., Gavrić, J., Gačić, Z. & Saičić, Z. (2013). Influence of some metal concentrations on the activity of antioxidant enzymes and concentrations of vitamin e and SH-groups in the digestive gland and gills of the freshwater bivalve *Unio tumidus* from the Serbian part of Sava River. *Ecological* *Indicators* **32**, 212–221.
95. *Bouldin, J. L., Farris, J. L., Moore, M. T., Smith, Jr. S. & Cooper, C. M. (2007). Assessment of diazinon toxicity in sediment and water of constructed wetlands using deployed *Corbicula fluminea* and laboratory testing. *Archives of Environmental Contamination and Toxicology* **53**, 174–182.
96. *Bourgeault, A., Cousin, C., Geertsen, V., Cassier-Chauvat, C., Chauvat, F., Durupthy, O., Chanéac, C. & Spalla, O. (2015). The challenge of studying TiO2 nanoparticle bioaccumulation at environmental concentrations: Crucial use of a stable isotope tracer. *Environmental Science and Technology* **49**, 2451–2459.
97. *Bourgeault, A. & Gourlay-Francé, C. (2013). Monitoring PAH contamination in water: Comparison of biological and physico-chemical tools. *Science of the Total Environment* **454–455**, 328–336.
98. *Bowen, Z. H., Malvestuto, S. P., Davies, W. D. & Crance, J. H. (1994). Evaluation of the mussel fishery in Wheeler Reservoir, Tennessee River. *Journal of Freshwater Ecology* **9**, 313–319.
99. *Brauer, H., Wagner, A., Boman, J. & Viet Binh, D. (2001). Use of total-reflection X-ray fluorescence in search of a biomonitor for environmental pollution in Vietnam. *Spectrochimica Acta - Part B Atomic Spectroscopy* **56**, 2147–2155.
100. *Brown, M. E., Curtin, T. M., Gallagher, C. J. & Halfman, J. D. (2012). Historic nutrient loading and recent species invasions caused shifts in water quality and zooplankton demography in two Finger Lakes (New York, USA). *Journal of Paleolimnology* **48**, 623–639.
101. *Bruesewitz, D. A., Tank, J. L., Bernot, M. J., Richardson, W. B. & Strauss, E. A. (2006). Seasonal effects of the zebra mussel (*Dreissena polymorpha*) on sediment denitrification rates in Pool 8 of the Upper Mississippi River. *Canadian Journal of Fisheries and Aquatic Sciences* **63**, 957–969.
102. *Bucci, J. P., Levine, J. F. & Showers, W. J. (2011). Spatial variability of the stable isotope (δ 15N) composition in two freshwater bivalves (*Corbicula fluminea* and *Elliptio* *complanata*). *Journal of Freshwater Ecology* **26**, 19–24.
103. *Bucci, J. P., Showers, W. J., Genna, B. & Levine, J. F. (2009). Stable oxygen and carbon isotope profiles in an invasive bivalve (*Corbicula fluminea*) in North Carolina watersheds. *Geochimica et Cosmochimica Acta* **73**, 3234–3247.
104. *Budd, C., Potekhina, I. & Lillie, M. (2020). Continuation of fishing subsistence in the Ukrainian Neolithic: diet isotope studies at Yasinovatka, Dnieper Rapids. *Archaeological and Anthropological Sciences* **12**, 64.
105. Buelow, C. A. & Waltham, N. J. (2020). Restoring tropical coastal wetland water quality: ecosystem service provisioning by a native freshwater bivalve. *Aquatic Sciences* **82**, 1–16.
106. *Bullard, A. E. & Hershey, A. E. (2013). Impact of *Corbicula fluminea* (Asian clam) on seston in an urban stream receiving wastewater effluent. *Freshwater Science* **32**, 976–990.
107. *Burket, S. R., White, M., Ramirez, A. J., Stanley, J. K., Banks, K. E., Waller, W. T., Chambliss, C. K. & Brooks, B. W. (2019). *Corbicula fluminea* rapidly accumulate pharmaceuticals from an effluent dependent urban stream. *Chemosphere* **224**, 873–883.
108. Burleigh, R. (1983). Two radiocarbon dates for freshwater shells from Hierakonpolis: Archaeological and geological interpretations. *Journal of Archaeological Science* **10**, 361–367.
109. Buskirk, B. L., Bourgeois, J., Meyer, H. W., Nesbitt, E. A. & DeVore, M. L. (2016). Freshwater molluscan fauna from the Florissant Formation, Colorado: paleohydrologic reconstruction of a latest Eocene lake. *Canadian Journal of Earth Sciences* **53**, 630–643.
110. *Buynevich, I. V., Damušyte, A., Bitinas, A., Olenin, S., Mažeika, J. & Petrošius, R. (2011). Pontic-Baltic pathways for invasive aquatic species: Geoarchaeological implications. *Geological Society of America* **473**, 189–196.
111. *Bykova, O., Laursen, A., Bostan, V., Bautista, J. & McCarthy, L. (2006). Do zebra mussels (*Dreissena polymorpha*) alter lake water chemistry in a way that favours *Microcystis* growth? *Science of the Total Environment* **371**, 362–372.
112. Cadena, E.-A. & Casado-Ferrer, I. (2019). Late Miocene freshwater mussels from the intermontane Chota Basin, northern Ecuadorean Andes. *Journal of South American Earth Sciences* **89**, 39–46.
113. Çakirlar, C. & Şeşen, R. (2013). Reading between the lines: δ18O and δ13C isotopes of *Unio* elongatulus shell increments as proxies for local palaeoenvironments in mid-Holocene northern Syria. *Archaeological and Anthropological Sciences* **5**, 85–94.
114. *Camusso, M., Balestrini, R. & Binelli, A. (2001). Use of zebra mussel (*Dreissena polymorpha*) to assess trace metal contamination in the largest Italian subalpine lakes. *Chemosphere* **44**, 263–270.
115. *Camusso, M., Balestrini, R., Muriano, F. & Mariani, M. (1994). Use of freshwater mussel *Dreissena polymorpha* to assess trace metal pollution in the lower River Po (Italy). *Chemosphere* **29**, 729–745.
116. *Canale, R. P. & Chapra, S. C. (2002). Modeling zebra mussel impacts on water quality of Seneca River, New York. *Journal of Environmental Engineering* **128**, 1158–116.
117. *Carrasco, L., Díez, S., Soto, D. X., Catalan, J. & Bayona, J. M. (2008). Assessment of mercury and methylmercury pollution with zebra mussel (*Dreissena polymorpha*) in the Ebro River (NE Spain) impacted by industrial hazardous dumps. *Science of the Total Environment* **407**, 178–184.
118. Cataldo, D., Boltovskoy, D., Stripeikis, J. & Pose, M. (2001*a*). Condition index and growth rates of field caged *Corbicula fluminea* (Bivalvia) as biomarkers of pollution gradients in the Paraná river delta (Argentina). *Aquatic Ecosystem Health & Management* **4**, 187–201.
119. *Cataldo, D., Colombo, J. C., Boltovskoy, D., Bilos, C. & Landoni, P. (2001*b*). Environmental toxicity assessment in the Paraná river delta (Argentina): simultaneous evaluation of selected pollutants and mortality rates of *Corbicula fluminea* (Bivalvia) early juveniles. *Environmental Pollution* **112**, 379–389.
120. Cha, Y. K., Stow, C. A., Nalepa, T. F. & Reckhow, K. H. (2011). Do invasive mussels restrict offshore phosphorus transport in Lake Huron? *Environmental Science & Technology* **45**, 7226–7231.
121. Chakraborty, A., Parveen, S., Chanda, D. K. & Aditya, G. (2020). An insight into the structure, composition and hardness of a biological material: the shell of freshwater mussels. *RSC Advances* **10**, 29543–29554.
122. Chakraborty, M., Bhattacharya, S., Bhattacharjee, P., Das, R. & Mishra, R. (2010). Prevention of the progression of adjuvant induced arthritis by oral supplementation of Indian fresh water mussel (*Lamellidens marginalis*) aqueous extract in experimental rats. *Journal of Ethnopharmacology* **132**, 316–320.
123. *Chakraborty, S., Ray, M. & Ray, S. (2013). Cell to organ: Physiological, immunotoxic and oxidative stress responses of *Lamellidens marginalis* to inorganic arsenite. *Ecotoxicology and Environmental Safety* **94**, 153–163.
124. Chazanah, N., Muntalif, B. S., Rahmayani, R. A. & Sudjono, P. (2020). Macrozoobentos distribution as a bioindicator of water quality in the upstream of the Citarum River. *Journal of Ecological Engineering* **21**, 10–17.
125. *Chen, J.-Z., He, Y.-P., Meng, S.-L., Hu, G.-D., Qu, J.-H. & Fan, L.-M. (2007). Purification effect of polyculture of fish-mussel in pond, a mode of circular economy. *Journal of Ecology and Rural Environment* **23**, 41–46.
126. *Chen, T.-Y., Lin, B.-C., Shiao, M.-S. & Pan, B. S. (2008). Lipid-lowering and LDL-oxidation inhibitory effects of aqueous extract of freshwater clam (*Corbicula fluminea*) - Using tilapia as an animal model. *Journal of Food Science* **73**, H148–H154.
127. *Chen, W.-Y. & Liao, C.-M. (2010). Dynamic features of ecophysiological response of freshwater clam to arsenic revealed by BLM-based toxicological model. *Ecotoxicology* **19**, 1074–1083.
128. Chen, W.-Y., Liao, C.-M., Jou, L.-J. & Jau, S.-F. (2010). Predicting bioavailability and bioaccumulation of arsenic by freshwater clam *Corbicula fluminea* using valve daily activity. *Environmental Monitoring and Assessment* **169**, 647–659.
129. *Chen, X., Bai, Z. & Li, J. (2019). The Mantle Exosome and MicroRNAs of *Hyriopsis cumingii* Involved in Nacre Color Formation. *Marine Biotechnology* **21**, 634–642.
130. *Chevreuil, M., Blanchard, M., Teil, M.-J., Carru, A.-M., Testard, P. & Chesterikoff A. (1996). Evaluation of the pollution by organochlorinated compounds (polychlorobiphenyls and pesticides) and metals Cd, Cr, Cu and Pb in the water and in the zebra mussel (*Dreissena* *polymorpha* Pallas) of the river seine. *Water, Air, and Soil Pollution* **88**, 371–381.
131. *Chijimatsu, T., Umeki, M., Kataoka, Y., Kobayashi, S., Yamada, K., Oda, H. & Mochizuki, S. (2013). Lipid components prepared from a freshwater Clam (*Corbicula fluminea*) extract ameliorate hypercholesterolaemia in rats fed high-cholesterol diet. *Food Chemistry* **136**, 328–334.
132. Chijimatsu, T., Umeki, M., Okuda, Y., Yamada, K., Oda, H. & Mochizuki, S. (2011). The fat and protein fractions of freshwater clam (*Corbicula fluminea*) extract reduce serum cholesterol and enhance bile acid biosynthesis and sterol excretion in hypercholesterolaemic rats fed a high-cholesterol diet. *British Journal of Nutrition* **105**, 526–534.
133. *Chmist, J. & Szoszkiewicz, K. (2017). Attempt at assessment of *Unio tumidus* bivalve mollusks suitability for monitoring water iron content. *Ochrona Srodowiska* **39**, 39–43.
134. Choi, M. H., Lee, K., Kim, M. Y., Shin, H.-I. & Jeong, D. (2019). *Pisidium coreanum* inhibits multinucleated osteoclast formation and prevents estrogen-deficient osteoporosis. *International Journal of Molecular Sciences* **20**, 6076.
135. *Chowdhury, G. W., Zieritz, A. & Aldridge, D. C. (2016). Ecosystem engineering by mussels supports biodiversity and water clarity in a heavily polluted lake in Dhaka, Bangladesh. *Freshwater Science* **35**, 188–199.
136. *Ciparis, S., Schreiber, M. E. & Voshell, Jr. J. R. (2012). Using watershed characteristics, sediment, and tissue of resident mollusks to identify potential sources of trace elements to streams in a complex agricultural landscape. *Environmental Monitoring and Assessment* **184**, 3109–3126.
137. *Clearwater, S. J., Hickey, C. W. & Thompson, K. J. (2014). The effect of chronic exposure to phosphorus-inactivation agents on freshwater biota. *Hydrobiologia* **728**, 51–65.
138. *Coelho, J. P., Lillebø, A. I., Crespo, D., Leston, S. & Dolbeth, M. (2018). Effect of the alien invasive bivalve *Corbicula fluminea* on the nutrient dynamics under climate change scenarios. *Estuarine, Coastal and Shelf Science* **204**, 273–282.
139. *Collas, F. P. L., Koopman, K. R., van der Velde, G. & Leuven, R. S. E. W. (2020). Quantifying the loss of filtration services following mass mortality of invasive dreissenid mussels. *Ecological Engineering* **149**, 105781.
140. *Collins, J., Andrus, C. F. T., Scott, R. J., Moe-Hoffman, A. & Peacock, E. (2020). Refit and oxygen isotope analysis of freshwater mussel shells from the Tillar Farms Site (3DR30), Southeast Arkansas. *Midcontinental Journal of Archaeology* **45**, 39–63.
141. *Colombo, J. C., Bilos, C., Campanaro, M., Rodriguez Presa, M. J. & Catoggio, J. A. (1995). Bioaccumulation of Polychlorinated Biphenyls and Chlorinated Pesticides by the Asiatic Clam *Corbicula fluminea*; its use as sentinel organism in the Rio de La Plata Estuary, Argentina. *Environmental Science and Technology* **29**, 914–927.
142. *Colombo, J. C., Brochu, C., Bilos, C., Landoni, P. & Moore, S. (1997). Long-Term accumulation of individual PCBs, Dioxins, furans, and trace metals in Asiatic clams from the Rio de la Plata Estuary, Argentina. *Environmental Science and Technology* **31**, 3551–3557.
143. *Company, R., Serafim, A., Lopes, B., Cravo, A., Shepherd, T. J., Pearson, G. & Bebianno, M. J. (2008). Using biochemical and isotope geochemistry to understand the environmental and public health implications of lead pollution in the lower Guadiana River, Iberia: A freshwater bivalve study. *Science of the Total Environment* **405**, 109–119.
144. *Contardo-Jara, V., Galanti, L. N., Amé, M. V., Monferrán, M. V., Wunderlin, D. A. & Wiegand, C. (2009*a*). Biotransformation and antioxidant enzymes of *Limnoperna fortunei* detect site impact in watercourses of Córdoba, Argentina. *Ecotoxicology and Environmental* *Safety* **72**, 1871–1880.
145. *Contardo-Jara, V., Krueger, A., Exner, H.-J. & Wiegand, C. (2009*b*). Biotransformation and antioxidant enzymes of *Dreissena polymorpha* for detection of site impact in watercourses of Berlin. *Journal of Environmental Monitoring* **11**, 1147–1156.
146. Contardo-Jara, V. & Wiegand, C. (2008). Molecular biomarkers of *Dreissena polymorpha* for evaluation of renaturation success of a formerly sewage polluted stream. *Environmental Pollution* **155**, 182–189.
147. *Cooper, S., Hare, L. & Campbell, P. G. C. (2010). Modeling cadmium uptake from water and food by the freshwater bivalve Pyganodon grandis. *Canadian Journal of Fisheries and Aquatic Sciences* **67**, 1874–1888.
148. *Corenblit, D., Julien, F., Steiger, J., Darrozes, J. & Mialet, B. (2013). High shell deposition of the invasive clam *Corbicula fluminea* (Müller, 1774) on alluvial bars: Exploratory investigations and biogeomorphological research perspectives [Dépôts massifs de coquilles du mollusque invasif *Corbicula fluminea* (Müller; 1774) sur les bancs alluviaux: Observations exploratoires et perspectives de recherche en biogéomorphologie]. *Geomorphologie: Relief, Processus, Environnement* **19**, 153–164.
149. *Crane, M., Delaney, P., Mainstone, C. & Clarke, S. (1995). Measurement by *in situ* bioassay of water quality in an agricultural catchment. *Water Research* **29**, 2441–2448.
150. *Cyr, H., Collier, K. J., Clearwater, S. J., Hicks, B. J. & Stewart, S. D. (2017). Feeding and nutrient excretion of the New Zealand freshwater mussel *Echyridella menziesii* (Hyriidae, Unionida): implications for nearshore nutrient budgets in lakes and reservoirs. *Aquatic* *Sciences* **79**, 557–571.
151. *Czarnezki, J. M. (1987). Use of the pocketbook mussel, *Lampsilis ventricosa*, for monitoring heavy metal pollution in an Ozark stream. *Bulletin of Environmental Contamination and Toxicology* **38**, 641–646.
152. Dai, J. P., Chen, J., Bei, Y. F., Han, B. X., Guo, S. B. & Jiang, L. L. (2010). Effects of pearl powder extract and its fractions on fibroblast function relevant to wound repair. *Pharmaceutical* *Biology* **48**, 122–127.
153. *Das, S. & Jana, B. B. (1999). Dose-dependent uptake and *Eichhornia*-induced elimination of cadmium in various organs of the freshwater mussel, *Lamellidens marginalis* (Linn.). *Ecological Engineering* **12**, 207–229.
154. Das, S. & Jana, B. B. (2003). *In situ* cadmium reclamation by freshwater bivalve *Lamellidens marginalis* from an industrial pollutant-fed river canal. *Chemosphere* **52**, 161-173.
155. *Das, S. & Jana, B. B. (2004). Distribution pattern of ambient cadmium in wetland ponds distributed along an industrial complex. *Chemosphere* **55**, 175–185.
156. *De La Cruz, C. P. P., De Vera, N. M., Lapie, L. P., Catalma, M. N. A. & Bunal, R. V. (2017). Bio accumulation and health risks assessment of lead (Pb) in freshwater Asian clams (*Corbicula fluminea*, Muller) from Laguna de Bay, Philippines. *Pollution Research*, **36** 366–372.
157. de Lafontaine, Y., Gagné, F., Blaise, C., Costan, G., Gagnon, P. & Chan, H. M. (2000). Biomarkers in zebra mussels (*Dreissena polymorpha*) for the assessment and monitoring of water quality of the St Lawrence River (Canada). *Aquatic Toxicology* **50**, 51–71.
158. De Stasio, B. T., Schrimpf, M. B., Beranek, A. E. & Daniels, W. C. (2008). Increased Chlorophyll a, phytoplankton abundance, and cyanobacteria occurrence following invasion of Green Bay, Lake Michigan by dreissenid mussels. *Aquatic Invasions* **3**, 21–27.
159. de Zwart, D., Kramer, K. J. M. & Jenner, H. A. (1995). Practical experiences with the biological early warning system “mosselmonitor”. *Environmental Toxicology and Water Quality* **10**, 237–247.
160. Debruyne, S. (2010). Tools and souvenirs: the shells from Kilise Tepe (1994)—(1998). *Anatolian Studies*, 149–160.
161. *Dee, K. H., Abdullah, F., Md Nasir, S. N. A., Appalasamy, S., Mohd Ghazi, R. & Eh Rak, A. (2019). Health risk assessment of heavy metals from smoked *Corbicula fluminea* collected on roadside vendors at Kelantan, Malaysia. *BioMed Research International* **2019**, 9596810.
162. *Demény, A., Kern, Z., Czuppon, G., Németh, A., Schöll-Barna, G., Siklósy, Z., Leél-Őssy, S., Cook, G., Serlegi, G., Bajnóczi, B., Sümegi, P., Király, Á., Kiss, V., Kulcsár G. & Bondár, M. (2019). Middle Bronze Age humidity and temperature variations, and societal changes in East-Central Europe. *Quaternary International* **504**, 80–95.
163. *Demény, A., Schöll-Barna, G., Fórizs, I., Osán, J., Sümegi, P. & Bajnóczi, B. (2012). Stable isotope compositions and trace element concentrations in freshwater bivalve shells (*Unio* sp.) as indicators of environmental changes at Tiszapüspöki, eastern Hungary. *Central European Geology* **55**, 441–460.
164. *Depew, D. C., Koehler, G. & Hiriart-Baer, V. (2018). Phosphorus dynamics and availability in the nearshore of Eastern Lake Erie: insights from oxygen isotope ratios of phosphate. *Frontiers in Marine Science* **5**, 215.
165. Di Fiori, E., Pizarro, H., dos Santos Afonso, M. & Cataldo, D. (2012). Impact of the invasive mussel *Limnoperna fortunei* on glyphosate concentration in water. *Ecotoxicology and Environmental Safety* **81**, 106–113.
166. Diggins, T. P., Baier, R. E., Meyer, A. E. & Forsberg, R. L. (2002). Potential for selective, controlled biofouling by *Dreissena* species to intercept pollutants from industrial effluents. *Biofouling* **18**, 29–36.
167. *Dionisio Pires, L. M., Bontes, B. M., Samchyshyna, L., Jong, J., Van Donk, E. & Ibelings, B. W. (2007). Grazing on microcystin-producing and microcystin-free phytoplankters by different filter-feeders: Implications for lake restoration. *Aquatic Sciences* **69**, 534–543.
168. *Dionisio Pires, L. M., Bontes, B. M., Van Donk, E. & Ibelings, B. W. (2005*a*). Grazing on colonial and filamentous, toxic and non-toxic cyanobacteria by the zebra mussel *Dreissena* *polymorpha*. *Journal of Plankton Research* **27**, 331–339.
169. *Dionisio Pires, L. M., Ibelings, B. W., Brehm, M. & Van Donk, E. (2005*b*). Comparing grazing on lake seston by *Dreissena* and *Daphnia*: Lessons for biomanipulation. *Microbial Ecology* **50**, 242–252.
170. *Dobson, E. P. & Mackie, G. L. (1998). Increased deposition of organic matter, polychlorinated biphenyls, and cadmium by zebra mussels (*Dreissena polymorpha*) in western Lake Erie. *Canadian Journal of Fisheries and Aquatic Sciences* **55**, 1131–1139.
171. Domingues, A., Rosa, I. C., Pinto da Costa, J., Rocha-Santos, T. A. P., Gonçalves, F. J. M., Pereira, R. & Pereira, J. L. (2020). Potential of the bivalve *Corbicula fluminea* for the remediation of olive oil wastewaters. *Journal of Cleaner Production* **252**, 119773.
172. *Dong, X., Lv, L., Zhao, W., Yu, Y. & Liu, Q. (2018). Optimization of integrated multi-trophic aquaculture systems for the giant freshwater prawn *Macrobrachium rosenbergii*. *Aquaculture Environment Interactions* **10**, 547–556.
173. *Downing, S., Contardo-Jara, V., Pflugmacher, S. & Downing, T. G. (2014). The fate of the cyanobacterial toxin β-N-methylamino-l-alanine in freshwater mussels. *Ecotoxicology* *and Environmental Safety* **101**, 51–58.
174. *Dresler, P. V. & Cory, R. L. (1980). The asiatic clam, *Corbicula fluminea* (Müller), in the tidal Potomac River, Maryland. *Estuaries* **3**, 150–151.
175. *Drouillard, K. G., Chan, S., O'Rourke, S., Douglas Haffner, G. & Letcher, R. J. (2007). Elimination of 10 polybrominated diphenyl ether (PBDE) congeners and selected polychlorinated biphenyls (PCBs) from the freshwater mussel, *Elliptio complanata*. *Chemosphere* **69**, 362–370.
176. *Drouillard, K. G., Cook, M., Leadley, T. A., Drca, P., Briggs, T. & Haffner, G. D. (2016). Quantitative biomonitoring in the Detroit River using *Elliptio complanata*: Verification of steady state correction factors and temporal trends of PCBs in water between 1998 and 2015. *Bulletin of Environmental Contamination and Toxicology* **97**, 757–762.
177. *Drouillard, K. G., Jezdic, I., O'Rourke, S. M., Gewurtz, S. B., Raeside, A. A., Leadley, T. A., Drca, P. & Douglas Haffner, G. (2013). Spatial and temporal variability of PCBs in Detroit River water assessed using a long term biomonitoring program. *Chemosphere* **90**, 95–102.
178. *Duxbury, C. V., Grace, K. A., Poponi, A. & Auter, T. (2005). Copper and zinc accumulation by a transplanted bivalve, *Elliptio buckleyi*, in freshwater systems in Central Florida. *Journal of Freshwater Ecology* **20**, 661–669.
179. *Englund, V. P. M. & Heino, M. P. (1996). The freshwater mussel (*Anodonta anatina*) in.monitoring of 2,4,6-trichlorophenol: Behaviour and environmental variation considered. *Chemosphere* **32**, 391–403.
180. Erdoğan, F. & Erdoğan, M. (2015). Use of the Asian Clam (*Corbicula fluminea* Müller, 1774) as a biomechanical filter in ornamental fish culture. *Turkish Journal of Fisheries and Aquatic Sciences* **15**, 861–867.
181. *Evans, M. A., Fahnenstiel, G. & Scavia, D. (2011). Incidental oligotrophication of North American Great Lakes. *Environmental Science and Technology* **45**, 3297–3303.
182. *Eversole, A. G., Stuart, K. R. & Brune, D. E. (2008). Effect of temperature and phytoplankton concentration of Partitioned Aquaculture System water on freshwater mussel filtration. *Aquaculture Research* **39**, 1691–1696.
183. Fabra, M., Gordillo, S. & Piovano, E. L. (2012). Arqueomalacología en las costas de Ansenuza: análisis de una almeja nacarífera (*Anodontites trapesialis*) hallada en contexto funerario del sitio El Diquecito (Laguna Mar Chiquita, Córdoba). *Arqueología* **18**, 257–266.
184. *Facchetti, S. V., La Spina, R., Fumagalli, F., Riccardi, N., Gilliland, D. & Ponti, J. (2020). Detection of metal-doped fluorescent PVC microplastics in freshwater mussels. *Nanomaterials* **10**, 2363.
185. *Falfushynska, H. I., Delahaut, L., Stolyar, O. B., Geffard, A. & Biagianti-Risbourg, S. (2009). Multi-biomarkers approach in different organs of *Anodonta cygnea* from the Dnister Basin (Ukraine). *Archives of Environmental Contamination and Toxicology* **57**, 86–95.
186. *Falfushynska, H. I., Gnatyshyna, L. L., Farkas, A., Vehovszky, Á., Gyori, J. & Stoliar, O. B. (2010). Vulnerability of biomarkers in the indigenous mollusk *Anodonta cygnea* to spontaneous pollution in a transition country. *Chemosphere* **81**, 1342–1351.
187. *Farris, J. L., Grudzien, J. L., Belanger, S. E., Cherry, D. S. & Cairns, Jr. J. (1994). Molluscan cellulolytic activity responses to zinc exposure in laboratory and field stream comparisons. *Hydrobiologia* **287**, 161–178.
188. *Fengqin, C., Hucai, Z., Yue, C., Mingsheng, Y., Jie, N., Hongfang, F., Guoliang, L., Wenxiang, Z., Yanbin, L. & Lunqing, Y. (2008). Sedimentation geochemistry and environmental changes during the Late Pleistocene of Paleolake Qarhan in the Qaidam Basin. *Journal of China University of Geosciences* **19**, 1–8.
189. Fera, S. A., Rennie, M. D. & Dunlop, E. S. (2017). Broad shifts in the resource use of a commercially harvested fish following the invasion of dreissenid mussels. *Ecology* **98**, 1681–1692.
190. *Fernald, S. H., Caraco, N. F. & Cole, J. J. (2007). Changes in cyanobacterial dominance following the invasion of the zebra mussel *Dreissena polymorpha*: Long-term results from the Hudson River estuary. *Estuaries and Coasts* **30**, 163–170.
191. Ferreira, R., Gomes, J., Martins, R. C., Costa, R. & Quinta-Ferreira, R. M. (2018). Winery wastewater treatment by integrating Fenton's process with biofiltration by *Corbicula fluminea*. *Journal of Chemical Technology & Biotechnology* **93**, 333–339.
192. *Ferreira-Rodríguez, N., Iglesias, J. & Pardo, I. (2019). *Corbicula fluminea* affecting supporting ecosystem services through nutrient and biogenic matter incorporation in invaded estuaries. *Fundamental and Applied* *Limnology* **192**, 269–280.
193. Figueiredo, S. A., Loureiro, J. & Boaventura, R. (2005). Natural waste materials containing chitin as adsorbents for textile dyestuffs: Batch and continuous studies. *Water Research* **39**, 4142–4152.
194. Fiske, D. & Shepherd, J. (2007). Continuity and change in Chinese freshwater pearl culture. *Gems & Gemology* **43**, 138–145.
195. *Foster, R. B. & Bates, J. M. (1978). Use of freshwater mussels to monitor point source industrial discharges. *Environmental Science and Technology* **12**, 958–962.
196. *Francoeur, S. N., Winslow, K. A. P., Miller, D. & Peacor, S. D. (2017). Mussel-derived stimulation of benthic filamentous algae: The importance of nutrients and spatial scale. *Journal of* *Great Lakes Research* **43**, 69–79.
197. *Frau, D., Molina, F. R., Devercelli, M. & de Paggi, S. J. (2013). The effect of an invading filter-feeding bivalve on a phytoplankton assemblage from the Paraná system: A mesocosm experiment. *Marine and Freshwater Behaviour and Physiology* **45**, 303–316.
198. *Freitas, M., Azevedo, J., Carvalho, A. P., Campos, A. & Vasconcelos, V. (2014). Effects of storage, processing and proteolytic digestion on microcystin-LR concentration in edible clams. *Food and Chemical Toxicology* **66**, 217–223.
199. Friedland, R., Buer, A.-L., Dahlke, S. & Schernewski, G. (2019). Spatial effects of different zebra mussel farming strategies in an eutrophic Baltic Lagoon. *Frontiers in Environmental Science* **6**, 158.
200. *Frischer, M. E., Nierzwicki-Bauer, S. A., Parsons, R. H., Vathanodorn, K. & Waitkus, K. R. (2000). Interactions between zebra mussels (*Dreissena polymorpha*) and microbial communities. *Canadian Journal of Fisheries and Aquatic Sciences* **57**, 591–599.
201. Fritts, A. K., Fritts, M. W., Haag, W. R., DeBoer, J. A. & Casper, A. F. (2017). Freshwater mussel shells (Unionidae) chronicle changes in a North American river over the past 1000 years. *Science of The Total Environment* **575**, 199–206.
202. *Fry, B. & Allen, Y. C. (2003). Stable isotopes in Zebra mussels as bioindicators of river-watershed linkages. *River Research and Applications* **19**, 683–696.
203. *Gačić, Z., Kolarević, S., Sunjog, K., Kračun-Kolarević, M., Paunović, M., Knežević-Vukčević, J. & Vuković-Gačić, B. (2014). The impact of *in vivo* and *in vitro* exposure to base analogue 5-FU on the level of DNA damage in haemocytes of freshwater mussels *Unio pictorum* and *Unio tumidus*. *Environmental Pollution* **191**, 145–150.
204. *Gaigalas, A., Pazdur, A., Michczynski, A., Pawlyta, J., Kleišmantas, A., Melešyte, M., Rudnickaite E., Kazakauskas, V. & Vainorius, J. (2013). Peculiarities of sedimentation conditions in the oxbow lakes of Dubysa River (Lithuania). *Geochronometria* **40**, 22–32.
205. *Gaillard, B., Lazareth, C. E., Lestrelin, H., Dufour, E., Santos, R. V., Freitas, C. E. C. & Pouilly, M. (2019). Seasonal oxygen isotope variations in freshwater bivalve shells as recorders of Amazonian rivers hydrogeochemistry. *Isotopes in Environmental and Health Studies* **55**, 511–525.
206. *Gan, C., Champagne, P. & Hall, G. (2018). Pilot-scale evaluation of semi-passive treatment technologies for the treatment of septage under temperate climate conditions. *Journal* *of Environmental Management* **216**, 357–371.
207. *Ganjali, S. & Mortazavi, S. (2014). The swan mussel (*Anodonta cygnea*) in Anzali wetland of Iran, a potential biomonitor for Cd and Pb. *Bulletin of Environmental Contamination and Toxicology* **93**, 154–158.
208. *Gao, H., Qian, X., Wu, H., Li, H., Pan, H. & Han, C. (2017). Combined effects of submerged macrophytes and aquatic animals on the restoration of a eutrophic water body—A case study of Gonghu Bay, Lake Taihu. *Ecological Engineering* **102**, 15–23.
209. Garvey, J. (2017). Australian Aboriginal freshwater shell middens from late Quaternary northwest Victoria: prey choice, economic variability and exploitation. *Quaternary International* **427**, 85–102.
210. *Gattás, F., Espinosa, M., Babay, P., Pizarro, H. & Cataldo, D. (2020). Invasive species *versus* pollutants: Potential of *Limnoperna fortunei* to degrade glyphosate-based commercial formulations. *Ecotoxicology and Environmental Safety* **201**, 110794.
211. *Gattás, F., Vinocur, A., Graziano, M., dos Santos Afonso, M., Pizarro, H. & Cataldo, D. (2016). Differential impact of *Limnoperna* fortunei-herbicide interaction between Roundup Max® and glyphosate on freshwater microscopic communities. *Environmental Science* *and Pollution Research* **23**, 18869–18882.
212. Géba, E., Aubert, D., Durand, L., Escotte, S., La Carbona, S., Cazeaux, C., Bonnard, I., Bastien, F., Ladeiro, M. P. & Dubey, J. P. (2020*a*). Use of the bivalve *Dreissena polymorpha* as a biomonitoring tool to reflect the protozoan load in freshwater bodies. *Water Research* **170**, 115297.
213. *Géba, E., Rousseau, A., Le Guernic, A., Escotte-Binet, S., Favennec, L., La Carbona, S., Gargala, G., Dubey, J. P.., Villena, I., Betoulle, S., Aubert, D. & Bigot-Clivot, A. (2020*b*). Survival and infectivity of *Toxoplasma gondii* and *Cryptosporidium parvum* oocysts bioaccumulated by *Dreissena polymorpha*. *Journal of Applied Microbiology* **130**, 504–515.
214. *Genç, T. O., Po, B. H. K., Yilmaz, F., Lau, T.-C., Wu, R. S. S. & Chiu, J. M. Y. (2018). Differences in metal profiles revealed by native mussels and artificial mussels in Sariçay Stream, Turkey: Implications for pollution monitoring. *Marine and Freshwater Research* **69**, 1372–1378.
215. *Gewurtz, S. B., Drouillard, K. G., Lazar, R. & Haffner, G. D. (2002). Quantitative biomonitoring of PAHs using the barnes mussel (*Elliptio complanata*). *Archives of Environmental Contamination and Toxicology* **43**, 497–504.
216. *Gewurtz, S. B., Lazar, R. & Haffner, G. D. (2003). Biomonitoring of bioavailable PAH and PCB water concentrations in the Detroit River using the freshwater mussel, *Elliptio* *complanata*. *Journal of Great Lakes Research* **29**, 242–255.
217. *Ghosh, S., Mal, M. & Mandal, S. (2020*a*). A dynamic model of cadmium bioaccumulation in Lamellidens marginalis, an edible shellfish in India. *Ecological Modelling* 419, 108957.
218. Ghosh, S., Mondal, A., Gangopadhyay, S. & Mandal, S. (2020*b*). Cadmium bioaccumulation in *Lamellidens marginalis* and human health risk assessment: A case study in India. *Human and Ecological Risk Assessment: An International Journal* **26**, 713–725.
219. *Giari, L., Vincenzi, F., Fano, E. A., Graldi, I., Gelli, F. & Castaldelli, G. (2017). Sensitivity to selected contaminants in a biological early warning system using *Anodonta woodiana* (Mollusca). *Water SA* **43**, 200–208.
220. *Golovko, N., Golovko, T. & Gelikh, A. (2015). Investigation of amino acid structure of proteins of freshwater bivalve mussels from the genus *Anodonta* of the northern Ukraine. *Eastern-European Journal of Enterprise Technologies* **5**, 10–16.
221. *Gomes, J. F., Lopes, A., Gonçalves, D., Luxo, C., Gmurek, M., Costa, R., Quinta-Ferreira, R. M., Martins, R. C. & Matos, A. (2018). Biofiltration using *C. fluminea* for *E.coli* removal from water: Comparison with ozonation and photocatalytic oxidation. *Chemosphere* **208**, 674–681.
222. Good, S. C. (2004). Paleoenvironmental and paleoclimatic significance of freshwater bivalves in the Upper Jurassic Morrison Formation, Western Interior, USA. *Sedimentary Geology* **167**, 163–176.
223. *Graczyk, T. K., Conn, D. B., Lucy, F., Minchin, D., Tamang, L., Moura, L. N. S. & DaSilva, A. J. (2004). Human waterborne parasites in zebra mussels (*Dreissena polymorpha*) from the Shannon River drainage area, Ireland. *Parasitology Research* **93**, 385–391.
224. *Graczyk, T. K., Fayer, R., Conn, D. B. & Lewis, E. J. (1999). Evaluation of the recovery of waterborne *Giardia* cysts by freshwater clams and cyst detection in clam tissue. *Parasitology Research* **85**, 30–34.
225. *Graczyk, T. K., Fayer, R., Cranfield, M. R. & Conn, D. B. (1998*a*). Recovery of waterborne *Cryptosporidium parvum* oocysts by freshwater benthic clams (*Corbicula fluminea*). *Applied and Environmental Microbiology* **64**, 427–430.
226. *Graczyk, T. K., Lucy, F. E., Tamang, L., Minchin, D. & Miraflor, A. (2008). Assessment of human waterborne parasites in Irish river basin districts - Use of zebra mussels (*Dreissena* *polymorpha*) as bioindicators. *Aquatic Invasions* **3**, 305–313.
227. Graczyk, T. K., Marcogliese, D. J., de Lafontaine, Y., Da Silva, A. J., Mhangami-Ruwende, B. & Pieniazek, N. J. (2001). *Cryptosporidium parvum* oocysts in zebra mussels (*Dreissena polymorpha*): evidence from the St. Lawrence River. *Parasitology Research* **87**, 231–234.
228. *Graczyk, T. K., Ortega, Y. R. & Conn, D. B. (1998*b*). Recovery of waterborne oocysts of *Cyclospora* *cayetanensis* by Asian freshwater clams (*Corbicula fluminea*). *American Journal of* *Tropical Medicine and Hygiene* **59**, 928–932.
229. *Greene, S., McElarney, Y. R. & Taylor, D. (2015). Water quality effects following establishment of the invasive *Dreissena polymorpha* (Pallas) in a shallow eutrophic lake: implications for pollution mitigation measures. *Hydrobiologia* **743**, 237–253.
230. *Guerlet, E., Ledy, K., Meyer, A. & Giambérini, L. (2007). Towards a validation of a cellular biomarker suite in native and transplanted zebra mussels: A 2-year integrative field study of seasonal and pollution-induced variations. *Aquatic Toxicology* **81**, 377–388.
231. *Guerlet, E., Vasseur, P. & Giambérini, L. (2010). Spatial and temporal variations of biological responses to environmental pollution in the freshwater zebra mussel. *Ecotoxicology* *and Environmental Safety* **73**, 1170–1181.
232. *Guidi, P., Bernardeschi, M., Palumbo, M., Genovese, M., Scarcelli, V., Fiorati, A., Riva, L., Punta, C., Corsi, I. & Frenzilli, G. (2020). Suitability of a cellulose-based nanomaterial for the remediation of heavy metal contaminated freshwaters: a case-study showing the recovery of cadmium induced DNA integrity loss, cell proliferation increase, nuclear morphology and chromosomal alterations on *Dreissena polymorpha.* *Nanomaterials* **10**, 1837.
233. *Guilhermino, L., Vieira, L. R., Ribeiro, D., Tavares, A. S., Cardoso, V., Alves, A. & Almeida, J. M. (2018). Uptake and effects of the antimicrobial florfenicol, microplastics and their mixtures on freshwater exotic invasive bivalve *Corbicula fluminea*. *Science of the Total* *Environment* **622–623**, 1131–1142.
234. *Guimaraes, V. & Sigolo, J. B. (2008). Detection of contaminants in a bioindicator species (*Corbicula fluminea*)–Ribeira de Iguape River, Sao Paulo State. *Quimica Nova* **31**, 1696–1698.
235. *Haldar, A., Das, M., Chatterjee, R., Dey, T. K., Dhar, P. & Chakrabarti, J. (2018). Functional properties of protein hydrolysates from fresh water mussel *Lamellidens marginalis* (Lam.). *Indian Journal of Biochemistry and Biophysics* **55**, 105–113.
236. *Hamli, H., Idris, M. H., Abu Hena, M. K. & Wong, S. K. (2012). Taxonomic study of edible bivalve from selected division of Sarawak, Malaysia. *International Journal of Zoological Research* **8**, 52–58.
237. Hansen, G. J., Ahrenstorff, T. D., Bethke, B. J., Dumke, J. D., Hirsch, J., Kovalenko, K. E., LeDuc, J. F., Maki, R. P., Rantala, H. M. & Wagner, T. (2020). Walleye growth declines following zebra mussel and *Bythotrephes* invasion. *Biological Invasions* **22**, 1481–1495.
238. *Hanuš, L. O., Levitsky, D. O., Shkrob, I. & Dembitsky, V. M. (2009). Plasmalogens, fatty acids and alkyl glyceryl ethers of marine and freshwater clams and mussels. *Food Chemistry* **116**, 491–498.
239. *Hartley, D. M. & Johnston, J. B. (1983). Use of the freshwater clam *Corbicula manilensis* as a monitor for organochlorine pesticides. *Bulletin of Environmental Contamination and Toxicology* **31**, 33–40.
240. Harzhauser, M. & Tempfer, P. M. (2004). Late Pannonian wetland ecology of the Vienna Basin based on molluscs and lower vertebrate assemblages (Late Miocene, MN 9, Austria). *Courier Forschungsinstitut Senckenberg* **246**, 55–68.
241. Hassan, F. A., Hamdan, M. A., Flower, R. J. & Keatings, K. (2012). The oxygen and carbon isotopic records in Holocene freshwater mollusc shells from the Faiyum paleolakes, Egypt: Their paloenvironmental and paleoclimatic implications. *Quaternary International* **266**, 175–187.
242. Hayer, F., Wagner, P. & Pihan, J. C. (1996). Monitoring of extractable organic halogens (EOX) in chlorine bleached pulp and paper mill effluents using four species of transplanted aquatic mollusks. *Chemosphere* **33**, 2321–2334.
243. He, H., Liu, X., Liu, X., Yu, J., Li, K., Guan, B., Jeppesen, E. & Liu, Z. (2014). Effects of cyanobacterial blooms on submerged macrophytes alleviated by the native Chinese bivalve *Hyriopsis cumingii*: A mesocosm experiment study. *Ecological Engineering* **71**, 363–367.
244. *Heifrich, L. A., Zimmerman, M. & Weigmann, D. L. (1995). Control of suspended solids and phytoplankton with fishes and a mussel. *JAWRA Journal of the American Water Resources Association* **31**, 307–316.
245. *Helama, S. & Nielsen, J. K. (2008). Construction of statistically reliable sclerochronology using subfossil shells of river pearl mussel. *Journal of Paleolimnology* **40**, 247–261.
246. *Helama, S., Nielsen, J. K. & Valovirta, I. (2009). Evaluating contemporaneity and post-mortem age of malacological remains using sclerochronology and dendrochronology. *Archaeometry* **51**, 861–877.
247. Herve, S., Paasivirta, J. & Heinonen, P. (2001). Trends of organochlorine compounds in Finnish inland waters. *Environmental Science and Pollution Research* **8**, 19–26.
248. *Herve, S., Prest, H. F., Heinonen, P., Hyötyläinen, T., Koistinen, J. & Paasivirta, J. (1995). Lipid-Filled semipermeable membrane devices and mussels as samplers of organochlorine compounds in lake water. *Environmental Science and Pollution Research* **2**, 24–30.
249. Hewitt, T. L., Bergner, J. L., Woolnough, D. A. & Zanatta, D. T. (2018). Phylogeography of the freshwater mussel species *Lasmigona costata*: testing post–glacial colonization hypotheses. *Hydrobiologia* **810**, 191–206.
250. *Hickey, C. W., Roper, D. S. & Buckland, S. J. (1995). Metal concentrations of resident and transplanted freshwater mussels *Hyridella menziesi* (Unionacea: Hyriidae) and sediments in the Waikato River, New Zealand. *Science of the Total Environment* **175**, 163–177.
251. *High, K. A., Barthet, V. J., McLaren, J. W. & Blais, J.-S. (1997). Characterization of metallothionein-like proteins from zebra mussels (*Dreissena polymorpha*). *Environmental Toxicology and Chemistry* **16**, 1111–1118.
252. *Hoellein, T. J., Zarnoch, C. B., Bruesewitz, D. A. & DeMartini, J. (2017). Contributions of freshwater mussels (Unionidae) to nutrient cycling in an urban river: filtration, recycling, storage, and removal. *Biogeochemistry* **135**, 307–324.
253. *Hogan, L. S., Marschall, E., Folt, C. & Stein, R. A. (2007). How non-native species in Lake Erie influence trophic transfer of mercury and lead to top predators. *Journal of Great Lakes* *Research* **33**, 46–61.
254. Holland, R. E. (1993). Changes in planktonic diatoms and water transparency in Hatchery Bay, Bass Island area, western Lake Erie since the establishment of the zebra mussel. *Journal of Great Lakes Research* **19**, 617–624.
255. Horikoshi, M. (2020). The bounty of Lake Biwa and traditional culinary culture. In *Lake Biwa: interaction between nature and people*. (eds H. Kawanabe, M. Nishino and M. Maehata), pp. 467–472. Springer Nature, Cham, Switzerland.
256. Hossain, A., Bhattacharyya, S. R. & Aditya, G. (2015). Biosorption of cadmium by waste shell dust of fresh water mussel *Lamellidens marginalis*: implications for metal bioremediation. *ACS Sustainable Chemistry & Engineering* **3**, 1−8.
257. *Howell, E. T. (2018). Influences on water quality and abundance of *Cladophora*, a shore-fouling green algae, over urban shoreline in Lake Ontario. *Water (Switzerland)* **10**, 1569.
258. *Hoyle, J. A., Bowlby, J. N. & Morrison, B. J. (2008). Lake whitefish and walleye population responses to dreissenid mussel invasion in eastern Lake Ontario. *Aquatic Ecosystem* *Health and Management* **11**, 403–411.
259. Hsieh, C.-C., Lin, M.-S., Hua, K.-F., Chen, W.-J. & Lin, C.-C. (2017). Neuroprotection by freshwater clam extract against the neurotoxin MPTP in C57BL/6 mice. *Neuroscience Letters* **642**, 51–58.
260. *Hsu, C.-L., Hsu, C.-C. & Yen, G.-C. (2010). Hepatoprotection by freshwater clam extract against CCl4-induced hepatic damage in rats. *American Journal of Chinese Medicine* **38**, 881–894.
261. Hu, S., Wang, Y. & Han, H. (2011). Utilization of waste freshwater mussel shell as an economic catalyst for biodiesel production. *Biomass and Bioenergy* **35**, 3627–3635.
262. *Hua, D. & Neves, R. J. (2007). Captive survival and pearl culture potential of the pink heelsplitter *Potamilus alatus*. *North American Journal of Aquaculture* **69**, 147–158.
263. *Huang, X., Luo, D., Zhao, D., Li, N., Xiao, T., Liu, J., Wei, L., Liu, Y., Liu, L. & Liu, G. (2019). Distribution, source and risk assessment of heavy metal(oid)s in water, sediments, and *Corbicula fluminea* of Xijiang River, China. *International Journal of Environmental* *Research and Public Health* **16**, 1823.
264. *Hull, M. S., Chaurand, P., Rose, J., Auffan, M., Bottero, J.-Y., Jones, J. C., Schultz, I. R. & Vikesland, P. J. (2011). Filter-feeding bivalves store and biodeposit colloidally stable gold nanoparticles. *Environmental Science and Technology* **45**, 6592–6599.
265. *Huyvaert, K. P., Carlson J. S., Bentler K. T., Cobble K. R., Nolte D. L. & Franklin A. B. (2012). Freshwater clams as bioconcentrators of avian influenza virus in water. *Vector-Borne and Zoonotic Diseases* **12**, 904–906.
266. Ilarri, M. I., Antunes, C., Guilhermino, L. & Sousa, R. (2011). Massive mortality of the Asian clam *Corbicula fluminea* in a highly invaded area. *Biological Invasions* **13**, 277–280.
267. *Inza, B., Ribeyre, F. & Boudou, A. (1998). Dynamics of cadmium and mercury compounds (inorganic mercury or methylmercury): Uptake and depuration in *Corbicula fluminea*. Effects of temperature and pH. *Aquatic Toxicology* **43**, 273–285.
268. *Iriani, D., Hasan, B. & Sumarto (2020). Physicochemical characteristics of freshwater mussel (*Pilsbryoconcha* sp.) shell from Sungai Paku village Riau Province Indonesia. *IOP Conference Series: Earth and Environmental Science* **430**, 012003.
269. Ismail, F. A., Aris, A. Z. & Latif, P. A. (2014). Dynamic behaviour of Cd2+ adsorption in equilibrium batch studies by CaCO3−rich *Corbicula fluminea* shell. *Environmental Science and Pollution Research* **21**, 344–354.
270. Ismail, N. S., Dodd, H., Sassoubre, L. M., Horne, A. J., Boehm, A. B. & Luthy, R. G. (2015). Improvement of urban lake water quality by removal of *Escherichia coli* through the action of the bivalve *Anodonta californiensis*. *Environmental Science & Technology* **49**, 1664–1672.
271. *Ismail, N. S., Müller, C. E., Morgan, R. R. & Luthy, R. G. (2014). Uptake of contaminants of emerging concern by the bivalves *Anodonta californiensis* and *Corbicula fluminea*. *Environmental Science and Technology* **48**, 9211–9219.
272. Ismail, N. S., Tommerdahl, J. P., Boehm, A. B. & Luthy, R. G. (2016). *Escherichia coli* reduction by bivalves in an impaired river impacted by agricultural land use. *Environmental Science & Technology* **50**, 11025–11033.
273. *Isparnadi, E., Hidayat, M., Aulanni’am, A. & Permatasari, N. (2015). Characterization and formulation of the bivalve *Anodonta*’s chitosan-platelet rich plasma-mesenchymal stem cells as a composite scaffold. *International Journal of ChemTech Research* **8**, 718–724.
274. *Ivanković, D., Pavičić, J., Beatović, V., Klobučar, R. S. & Klobučar, G. I. V. (2010). Inducibility of metallothionein biosynthesis in the whole soft tissue of zebra mussels *Dreissena* *polymorpha* exposed to cadmium, copper, and pentachlorophenol. *Environmental* *Toxicology* **25**, 198–211.
275. *Ivanova, E. V., Murdmaa, I. O., Chepalyga, A. L., Cronin, T. M., Pasechnik, I. V., Levchenko, O. V., Howe, S. S., Manushkina, A. V. & Platonova, E. A. (2007). Holocene sea-level oscillations and environmental changes on the Eastern Black Sea shelf. *Palaeogeography, Palaeoclimatology, Palaeoecology* **246**, 228–259.
276. Izumi, T., Yagita, K., Izumiyama, S., Endo, T. & Itoh, Y. (2012). Depletion of *Cryptosporidium parvum* oocysts from contaminated sewage by using freshwater benthic pearl clams (*Hyriopsis schlegeli*). *Applied and Environmental Microbiology* **78**, 7420–7428.
277. Jackson, D. & Jackson, D. (2008). Antecedentes arqueológicos del genero *Diplodon* (SPIX, 1827) (Bivalvia, Hyriidae) en Chile. *Gayana (Concepción)* **72**, 188–195.
278. *Jacobsen, B. N., Kjersgaard, D., Winther-Nielsen, M. & Gustavson, K. (2004). Combined chemical analyses and biomonitoring at Avedoere wastewater treatment plant in (2002). *Water* *Science and Technology* **50**, 37–43.
279. *James, W. F., Barko, J. W., Davis, M., Eakin, H. L., Rogala, J. T. & Miller, A. C. (2000). Filtration and excretion by zebra mussels: Implications for water quality impacts in Lake Pepin, upper Mississippi River. *Journal of Freshwater Ecology* **15**, 429–437.
280. Jana, B. B. & Das, S. (1997). Potential of freshwater mussel (*Lamellidens marginalis*) for cadmium clearance in a model system. *Ecological Engineering* **8**, 179–193.
281. *Javanshir, A., Shapoori, M. & Moëzzi, F. (2011). Impact of water hardness on cadmium absorption by four freshwater mollusks *Physa fontinalis*, *Anodonta cygnea*, *Corbicula fluminea* and *Dreissena polymorpha* from South Caspian Sea region. *Journal of Food,* *Agriculture and Environment* **9**, 763–767.
282. *Jeffree, R. A., Markich, S. J., Lefebvre, F., Thellier, M. & Ripoll, C. (1995). Shell microlaminations of the freshwater bivalve *Hyridella depressa* as an archival monitor of manganese water concentration: Experimental investigation by depth profiling using secondary ion mass spectrometry (SIMS). *Experientia* **51**, 838–848.
283. *Jeong, J. E., Kang, S. W., Hwang, H. J., Park, S. Y., Patnaik, B. B., Kim, C., Kim, S., Nam, M. M., Lee, J. B., Wang, T. H., Park, E. B., Yi, S. S., Han, Y. S., Lee, J. S., Park, H. S. & Lee, Y. S. (2015). Expressed sequence tag analysis and annotation of genetic information from the freshwater clam, *Pisidium* (*Neopisidium*) *coreanum* endemic to Korea. *Genes & Genomics* **37**, 1041–1049.
284. *Ji, L., Song, C., Cao, X., Zhou, Y. & Deng, D. (2015). Spatial variation in nutrient excretion by macrozoobenthos in a Chinese large shallow lake (Lake Taihu). *Journal of Freshwater* *Ecology* **30**, 169–180.
285. *Jia, Y., Wang, L., Qu, Z. & Yang, Z. (2018). Distribution, contamination and accumulation of heavy metals in water, sediments, and freshwater shellfish from Liuyang River, Southern China. *Environmental Science and Pollution Research* **25**, 7012–7020.
286. Jiao, D., Qu, R. T., Weng, Z. Y., Liu, Z. Q. & Zhang, Z. F. (2019). On the fracture mechanisms of nacre: Effects of structural orientation. *Journal of Biomechanics* **96**, 109336.
287. *Jin, C., Zhao, J.-Y., Liu, X.-J. & Li, J.-L. (2019). Expressions of shell matrix protein genes in the pearl sac and its correlation with pearl weight in the first 6 months of pearl formation in *Hyriopsis cumingii*. *Marine Biotechnology* **21**, 240–249.
288. *Jin, W., Bai, Z., Fu, L., Zhang, G. & Li, J. (2012). Genetic analysis of early growth traits of the triangle shell mussel, *Hyriopsis cumingii*, as an insight for potential genetic improvement to pearl quality and yield. *Aquaculture International* **20**, 927–933.
289. Johns, C. (2001). Spatial distribution of total cadmium, copper, and zinc in the zebra mussel (*Dreissena polymorpha*) along the upper St. Lawrence River. *Journal of Great Lakes Research* **27**, 354–366.
290. *Johns, C. (2011). Quagga mussels (*Dreissena bugensis*) as biomonitors of metal contamination: A case study in the upper St. Lawrence River. *Journal of Great Lakes Research* **37**, 140–146.
291. *Johns, C. (2012). Trends of total cadmium, copper, and zinc in the zebra mussel (*Dreissena* *polymorpha*) along the upper reach of the St. Lawrence River: (1994) - (2005). *Environmental Monitoring and Assessment* **184**, 5371–5385.
292. *Johnson, C. C., Njau, J. K., Van Damme, D., Schick, K. & Toth, N. (2016). Paleoecologic significance of malacofauna, Olduvai Gorge, Tanzania. *Palaios* **31**, 319–326.
293. Jones, B. A. (2019). Infant health impacts of freshwater algal blooms: Evidence from an invasive species natural experiment. *Journal of Environmental Economics and Management* **96**, 36–59.
294. Jou, L.-J., Chen, B.-C., Chen, W.-Y. & Liao, C.-M. (2016). Sensory determinants of valve rhythm dynamics provide *in situ* biodetection of copper in aquatic environments. *Environmental Science and Pollution Research* **23**, 5374–5389.
295. *Jou, L.-J., Chen, W.-Y. & Liao, C.-M. (2009). Online detection of waterborne bioavailable copper by valve daily rhythms in freshwater clam *Corbicula fluminea*. *Environmental Monitoring and Assessment* **155**, 257–272.
296. Kaandorp, R. J. G., Wesselingh, F. P. & Vonhof, H. B. (2006). Ecological implications from geochemical records of Miocene Western Amazonian bivalves. *Journal of South American Earth Sciences* **21**, 54–74.
297. Kang, Y., Xie, H., Zhang, J., Zhao, C., Wang, W., Guo, Y. & Guo, Z. (2018). Intensified nutrients removal in constructed wetlands by integrated *Tubifex tubifex* and mussels: Performance and mechanisms. *Ecotoxicology and Environmental Safety* **162**, 446–453.
298. *Karatayev, A. Y., Burlakova, L. E., Mehler, K., Barbiero, R. P., Hinchey, E. K., Collingsworth, P. D., Kovalenko, K. E. & Warren, G. (2018*a*). Life after *Dreissena*: The decline of exotic suspension feeder may have significant impacts on lake ecosystems. *Journal of Great* *Lakes Research* **44**, 650–659.
299. *Karatayev, A. Y., Burlakova, L. E., Mehler, K., Bocaniov, S. A., Collingsworth, P. D., Warren, G., Kraus, R. T. & Hinchey, E. K. (2018*b*). Biomonitoring using invasive species in a large lake: *Dreissena* distribution maps hypoxic zones. *Journal of Great Lakes Research* **44**, 639–649.
300. *Karmanova, A. N. & Zimin, A. A. (2020). Experimental model for study bacteriophage bioaccumulation by a bivalve *Unio pictorum* (L.1758). *Journal of Physics: Conference* *Series* **1701**, 012012.
301. *Karoui-Yaakoub, N., Mtimet, M.S., Bejaoui, S., Amri, L., Khalloufi, N., Ben Aissa, L. & Martinez-Navarro, B. (2016). Middle-to-Late Pleistocene malacofauna from the archeopaleontological site of Oued Sarrat (Tajerouine area, NW Tunisia). *Arabian Journal of Geosciences* **9**, 345.
302. *Karube, Z., Sakai, Y., Takeyama, T., Okuda, N., Kohzu, A., Yoshimizu, C., Nagata, T. & Tayasu, I. (2010). Carbon and nitrogen stable isotope ratios of macroinvertebrates in the littoral zone of Lake Biwa as indicators of anthropogenic activities in the watershed. *Ecological Research* **25**, 847–855.
303. *Kauss, P. B. & Hamdy, Y. S. (1985). Biological monitoring of organochlorine contaminants in the St. Clair and Detroit Rivers using introduced clams, *Elliptio complanatus*. *Journal of Great Lakes Research* **11**, 247–263.
304. Ke, L., Zhou, J., Lu, W., Gao, G. & Rao, P. (2011). The power of soups: Super-hero or team-work? *Trends in Food Science & Technology* **22**, 492–497.
305. *Kelemen, Z., Gillikin, D. P., Graniero, L. E., Havel, H., Darchambeau, F., Borges, A. V., Yambélé, A., Bassirou, A. & Bouillon, S. (2017). Calibration of hydroclimate proxies in freshwater bivalve shells from Central and West Africa. *Geochimica et Cosmochimica Acta* **208**, 41–62.
306. *Kerambrun, E., Ladeiro, M. P., Bigot-Clivot, A., Dedourge-Geffard, O., Dupuis, E., Villena, I., Aubert, D. & Geffard, A. (2016). Zebra mussel as a new tool to show evidence of freshwater contamination by waterborne *Toxoplasma gondii*. *Journal of Applied* *Microbiology* **120**, 498–508.
307. Khazri, A., Sellami, B., Dellali, M., Corcellas, C., Eljarrat, E., Barceló, D., Beyrem, H. & Mahmoudi, E. (2016). Diastereomeric and enantiomeric selective accumulation of cypermethrin in the freshwater mussel *Unio gibbus* and its effects on biochemical parameters. *Pesticide Biochemistry and Physiology* **129**, 83–88.
308. *Khudolei, V. V. & Sirenko, O. A. (1977). Tumor development in the Bivalve mollusk *Unio* *pictorum* induced by N-nitroso compounds. *Bulletin of Experimental Biology and* *Medicine* **83**, 684–686.
309. *Kim, B.-H., Lee, J.-H. & Hwang, S.-J. (2011). Inter- and intra-specific differences in filtering activities between two unionids, *Anodonta woodiana* and *Unio douglasiae*, in ambient eutrophic lake waters. *Ecological Engineering* **37**, 1957–1967.
310. *Kim, D. K., Zhang, W. T., Rao, Y. R., Watson, S., Mugalingam, S., Labencki, T., Dittrich, M., Morley, A. & Arhonditsis, G. B. (2013). Improving the representation of internal nutrient recycling with phosphorus mass balance models: A case study in the Bay of Quinte, Ontario, Canada. *Ecological Modelling* **256**, 53–68.
311. *Kim, M. S., Kwon, J. T., Lee, Y., Ha, S. Y., Hong, S., Yoon, S. H. & Shin, K. H. (2018). Bio-control of *Microcystis aeruginosa* bloom using various aquatic organisms by dual stable isotope (13C and15N) tracers. *Applied Ecology and Environmental Research* **16**, 931–953.
312. *Kindermann, K., Bubenzer, O., Nussbaum, S., Riemer, H., Darius, F., Pollath, N. & Smettan, U. (2006). Palaeoenvironment and holocene land use of Djara, western desert of Egypt. *Quaternary Science Reviews* **25**, 1619–1637.
313. *Kinney, R. M., Manos, Jr. C. G., Mills, E. L., Mellina, E. & Lisk, D. J. (1994). Zebra mussels (*Dreissena polymorpha*) as a biomonitoring tool for Sr90 contamination. *Chemosphere* **28**, 729–735.
314. *Kirillova, I. V., Levchenko, V. A., Ippolitov, A. P., Pokrovsky, B. G., Shishlina, N. I. & Yanina, T. A. (2018). The origin of objects of invertebrate descent from the Khvalynsk Eneolithic cemeteries (Northern Caspian region). *Quaternary International* **465**, 142–151.
315. *Kiryushin, Y. F., Kiryushin, K. Y., Schmidt, A. V. & Abdulganeyev, M. T. (2012). Ornaments made from animal teeth in human burials at Tuzovskiye bugry-1 and their relevance to ethnic processes in the Altai, 3rd millennium BC. *Archaeology, Ethnology and* *Anthropology of Eurasia* **40**, 59–66.
316. Kiryushin, Y. F., Kiryushin, K. Y., Schmidt, A. V., Kuzmenkin, D. V. & Abdulganeyev, M. T. (2011). Mollusk shells from burials of Tuzovskiye Bugry-1 as indicators of ethno-cultural processes in southern Siberia and western Central Asia in the 3rd millennium BC. *Archaeology, Ethnology and Anthropology of Eurasia* **39**, 37–45.
317. *Klamt, A. & Schernewski, G. (2013). Climate Change - A New Opportunity for Mussel Farming in the Southern Baltic? In *Climate Change Adaptation in Practice: From strategy development to implementation* (eds P. Schmidt-Thome and J. Klein), pp. 171–184. John Wiley & Sons, Ltd, Chichester, UK.
318. *Klerks, P. L. & Fraleigh, P. C. (1997). Uptake of nickel and zinc by the zebra mussel *Dreissena* *polymorpha*. *Archives of Environmental Contamination and Toxicology* **32**, 191–197.
319. Klimova, Y., Chuiko, G., Gapeeva, M. & Pesnya, D. (2017). The use of biomarkers of oxidative stress in zebra mussel *Dreissena polymorpha* (Pallas, 1771) for chronic anthropogenic pollution assessment of the Rybinsk Reservoir. *Contemporary Problems of Ecology* **10**, 178–183.
320. *Klishko, O. K., Kovychev, E. V., Vinarski, M. V., Bogan, A. E. & Yurgenson, G. A. (2020). The Pleistocene-Holocene aquatic molluscs as indicators of the past ecosystem changes in Transbaikalia (Eastern Siberia, Russia). *PLoS ONE* **15**, e0235588.
321. *Knoll, L. B., Sarnelle, O., Hamilton, S. K., Kissman, C. E. H., Wilson, A. E., Rose, J. B. & Morgan, M. R. (2008). Invasive zebra mussels (*Dreissena polymorpha*) increase cyanobacterial toxin concentrations in low-nutrient lakes. *Canadian Journal of Fisheries and Aquatic* *Sciences* **65**, 448–455.
322. *Knox, A. S. & Paller, M. H. (2020) Effect of bioturbation on contaminated sediment deposited over remediated sediment. *Science of the Total Environment* **713**, 136537.
323. *Koistinen, J., Herve, S., Ruokojärvi, P., Koponen, J. & Vartiainen, T. (2010). Persistent organic pollutants in two Finnish watercourses: Levels, congener profiles and source estimation by mussel incubation. *Chemosphere* **80**, 625–633.
324. *Kolarević, S., Knežević-Vukčević, J., Paunović, M., Kračun, M., Vasiljević, B., Tomović, J., Vuković-Gačić, B. & Gačić, Z. (2013). Monitoring of DNA damage in haemocytes of freshwater mussel *Sinanodonta woodiana* sampled from the Velika Morava River in Serbia with the comet assay. *Chemosphere* **93**, 243–251.
325. *Kolarević, S., Knezević-Vukćević, J., Paunović, M., Tomović, J., Gaćić, Z. & Vuković-Gaćić, B. (2011). The anthropogenic impact on water quality of the River Danube in Serbia: Microbiological analysis and genotoxicity monitoring. *Archives of Biological Sciences* **63**, 1209–1217.
326. *Konieczny, P., Tomaszewska-Gras, J., Andrzejewski, W., Mikołajczak, B., Urbańska, M., Mazurkiewicz, J. & Stangierski, J. (2016). DSC and electrophoretic studies on protein denaturation of *Anodonta woodiana* (Lea, 1834). *Journal of Thermal Analysis and* *Calorimetry* **126**, 69–75.
327. Korolev, A., Kochkina, A., Stashenkov, D., Khokhlov, A. & Roslyakova, N. (2018). The unique burial of the Ekaterinovsky Cape Early Eneolithic cemetery in the Middle Volga region. *Stratum Plus*, 285–302.
328. *Kováts, N., Abdel-Hameid, N.-A., Kovács, K. & Padisák, J. (2012). Evaluation of single and interactive toxicities of lead and iron using filtration rate of Zebra mussels (*Dreissena* *polymorpha*). *WIT Transactions on Ecology and the Environment* **162**, 577–585.
329. *Kraak, M. H. S., Kuipers, F., Schoon, H., de Groot, C. J. & Admiraal, W. (1994). The filtration rate of the zebra mussel *Dreissena polymorpha* used for water quality assessment in Dutch rivers. *Hydrobiologia* **294**, 13–16.
330. *Kraak, M. H. S., Scholten, M. C. Th., Peeters, W. H. M. & de Kock, W. Chr. (1991). Biomonitoring of heavy metals in the Western European Rivers Rhine and Meuse using the freshwater mussel *Dreissena polymorpha*. *Environmental Pollution* **74**, 101–114.
331. *Kumar, S., Pandey, R. K., Das, S. & Das, V. K. (2013). Temperature dependent mortality and behavioral changes in a freshwater mussel *Lamellidens marginalis* to dimethoate Exposure. *Journal of Environmental Biology* **34**, 165–170.
332. Kuzucuoğlu, C., Karabiyikoğlu, M., Fontugne, M., Pastre, J.-F. & Ercan, T. (1997). Environmental changes in Holocene lacustrine sequences from Karapinar in the Konya Plain (Turkey). *Third Millennium BC Climate Change and Old World Collapse*, pp. 451–463. Springer Berlin Heidelberg, Berlin, Heidelberg.
333. *Kwan, K. H. M., Chan, H. M. & de Lafontaine, Y. (2003). Metal contamination in zebra mussels (*Dreissena polymorpha*) along the St. Lawrence river. *Environmental Monitoring and Assessment* **88**, 193–219.
334. *La Guardia, M. J., Hale, R. C., Harvey, E., Mainor, T. M. & Ciparis, S. (2012). *In situ* accumulation of HBCD, PBDEs, and several alternative flame-retardants in the bivalve (*Corbicula* *fluminea*) and Gastropod (*Elimia proxima*). *Environmental Science and Technology* **46**, 5798–5805.
335. *Labrot, F., Ribera, D., Saint Denis, M. & Narbonne, J. F. (1996). *In vitro* and *in vivo* studies of potential biomarkers of lead and uranium contamination: Lipid peroxidation, acetylcholinesterase, catalase and glutathione peroxidase activities in three non-mammalian species. *Biomarkers* **1**, 21–28.
336. Labuschagne, M., Wepener, V., Nachev, M., Zimmermann, S., Sures, B. & Smit, N. J. (2020). The application of artificial mussels in conjunction with transplanted bivalves to assess elemental exposure in a platinum mining area. *Water* **12**, 32.
337. *Lacey, J. H., Leng, M. J., Peckover, E. N., Dean, J. R., Wilke, T., Francke, A., Zhang, X., Masi, A. & Wagner, B. (2018). Investigating the environmental interpretation of oxygen and carbon isotope data from whole and fragmented bivalve shells. *Quaternary Science Reviews* **194**, 55–61.
338. *Lake, J. L., McKinney, R. A., Osterman, F. A., Pruell, R. J., Kiddon, J., Ryba, S. A. & Libby, A. D. (2001). Stable nitrogen isotopes as indicators of anthropogenic activities in small freshwater systems. *Canadian Journal of Fisheries and Aquatic Sciences* **58**, 870–878.
339. *Laurent, T., Okuda, Y., Chijimatsu, T., Umeki, M., Kobayashi, S., Kataoka, Y., Tatsuguchi, I., Mochizuki, S. & Oda, H. (2013). Freshwater clam extract ameliorates triglyceride and cholesterol metabolism through the expression of genes involved in hepatic lipogenesis and cholesterol degradation in rats. *Evidence-based Complementary and Alternative* *Medicine* **2013**, 830684.
340. *LaValle, P. D., Brooks, A. & Lakhan, V. C. (1999). Zebra mussel wastes and concentrations of heavy metals on shipwrecks in western Lake Erie. *Journal of Great Lakes Research* **25**, 330–338.
341. *Le Goff, J., Gallois, J., Pelhuet, L., Devier, M. H., Budzinski, H., Pottier, D., André, V. & Cachot, J. (2006). DNA adduct measurements in zebra mussels, *Dreissena polymorpha*, Pallas. Potential use for genotoxicant biomonitoring of fresh water ecosystems. *Aquatic* *Toxicology* **79**, 55–64.
342. *Lebrun, J. D., Dufour, M., Uher, E., Faburé, J., Mons, R., Charlatchka, R., Gourlay-Francé, C., Fechner, L. C. & Ferrari, B. J. D. (2017). To what extend the dam dredging can influence the background level of metals in the Rhine River: Using chemical and biological long-term monitoring to answer. *Knowledge and Management of Aquatic Ecosystems* **2017**, 54.
343. Lécrivain, N., Duparc, A., Clément, B., Naffrechoux, E. & Frossard, V. (2020). Tracking sources and transfer of contamination according to pollutants variety at the sediment-biota interface using a clam as bioindicator in peri-alpine lakes. *Chemosphere* **238**, 124569.
344. *Lee, C.-C., Jhuang, Y.-F., Liu, L.-L., Hsieh, C.-Y., Chen, C. S. & Tien, C.-J. (2009). The major source and impact of phenyltin contamination on freshwater aquaculture clam *Corbicula fluminea* and wild golden apple snail *Pomacea canaliculata*. *Environmental Chemistry* **6**, 341–349.
345. *Lee, J. R. (2009). Patterns of preglacial sedimentation and glaciotectonic deformation within early Middle Pleistocene sediments at Sidestrand, north Norfolk, UK. *Proceedings of* *the Geologists' Association* **120**, 34–48.
346. *Lee, J. S. & Lee, B. G. (2005). Effects of salinity, temperature and food type on the uptake and elimination rates of Cd, Cr, and Zn in the Asiatic Clam *Corbicula fluminea*. *Ocean* *Science Journal* **40**, 79–89.
347. Leechman, D. (1949). Suggested use of clam shells. *American Antiquity* **15**, 56–56.
348. *Leonardt, S. (2014). Local production of shell beads in the forest of Northwest Patagonia. An approach from experimental archeology. *Relaciones* **39**, 463–482.
349. *Lepoutre, A., Grilot, T., Jean, S., Geffard, A. & Lance, E. (2020*a*). Free or protein-bound microcystin accumulation by freshwater bivalves as a tool to evaluate water contamination by *Microcystin*-producing cyanobacteria?. *Applied Sciences-Basel* **10**, 3426.
350. Lepoutre, A., Hervieux, J., Faassen, E. J., Zweers, A. J., Lurling, M., Geffard, A. & Lance, E. (2020*b*). Usability of the bivalves *Dreissena polymorpha* and *Anodonta anatina* for a biosurvey of the neurotoxin BMAA in freshwater ecosystems. *Environmental Pollution* **259**, 113885.
351. Lewis, J. P., Leng, M. J., Dean, J. R., Marciniak, A., Bar-Yosef Mayer, D. E. & Wu, X. (2017). Early Holocene palaeoseasonality inferred from the stable isotope composition of *Unio* shells from Çatalhöyük, Turkey. *Environmental Archaeology* **22**, 79–95.
352. *Li, D., Wang, J., Pi, J., Yu, J. & Zhang, T. (2019). Biota-sediment metal accumulation and human health risk assessment of freshwater bivalve *Corbicula fluminea* in Dongting Lake, China. *Environmental Science and Pollution Research* **26**, 14951–14961.
353. Li, F., Wu, N., Lu, H., Zhang, J., Wang, W., Ma, M., Zhang, X. & Yang, X. (2013). Mid-Neolithic exploitation of mollusks in the Guanzhong basin of northwestern China: preliminary results. *Plos One* **8**, e58999.
354. Li, H., Pan, L., Chen, T., Zhang, H., Zhang, L., Ye, Y. & Xia, M. (2013). Preparation of bio-filler from freshwater mussel shell and its surface property characterization. *Journal of Central South University (Science and Technology)* **4**, 1209–1214.
355. *Li, Z., Feng, C., Wu, Y. & Guo, X. (2020). Impacts of nanoplastics on bivalve: Fluorescence tracing of organ accumulation, oxidative stress and damage. *Journal of Hazardous* *Materials* **392**, 122418.
356. *Liang, S. M., Ji, H. M. & Li, X. W. (2020). Thickness-dependent mechanical properties of nacre in *Cristaria* *plicata* shell: Critical role of interfaces. *Journal of Materials Science and* *Technology* **44**, 1–8.
357. *Liao, C.-M., Jau, S.-F., Lin, C.-M., Jou, L.-J., Liu, C.-W., Liao, V.H.-C. & Chang, F.-J. (2009). Valve movement response of the freshwater clam *Corbicula fluminea* following exposure to waterborne arsenic. *Ecotoxicology* **18**, 567–576.
358. *Liao, C.-M., Jou, L.-J. & Chen, B.-C. (2005). Risk-based approach to appraise valve closure in the clam *Corbicula fluminea* in response to waterborne metals. *Environmental Pollution* **135**, 41–52.
359. *Liao, C.-M., Lin, C.-M., Jou, L.-J. & Chiang, K.-C. (2007). Linking valve closure behavior and sodium transport mechanism in freshwater clam *Corbicula fluminea* in response to copper. *Environmental Pollution* **147**, 656–667.
360. *Liao, H., Mutvei, H., Sjöström, M., Hammarström, L. & Li, J. (2000). Tissue responses to natural aragonite (*Margaritifera* shell) implants *in vivo*. *Biomaterials* **21**, 457–468.
361. Limburg, K. E., Luzadis, V. A., Ramsey, M., Schulz, K. L. & Mayer, C. M. (2010). The good, the bad, and the algae: Perceiving ecosystem services and disservices generated by zebra and quagga mussels. *Journal of Great Lakes Research* **36**, 86–92.
362. *Lin, J.-J., Liu, Y.-C., Chang, C.-J., Pan, M.-H., Lee, M.-F. & Pan, B. S. (2018). Hepatoprotective mechanism of freshwater clam extract alleviates non-alcoholic fatty liver disease: Elucidated: *In vitro* and *in vivo* models. *Food and Function* **9**, 6315–6325.
363. Lin, P. & Guo, L. (2016). Do invasive quagga mussels alter CO2 dynamics in the Laurentian Great Lakes? *Scientific Reports* **6**, 39078.
364. *Lin, Y.-H., Tsai, J.-S. & Chen, G.-W. (2017). Purification and identification of hypocholesterolemic peptides from freshwater clam hydrolysate with *in vitro* gastrointestinal digestion. *Journal of Food Biochemistry* **41**, e12385.
365. *Lin, Y.-H., Tsai, J.-S., Hung, L.-B. & Pan, B. S. (2011). Plasma lipid regulatory effect of compounded freshwater clam hydrolysate and *Gracilaria* insoluble dietary fibre. *Food* *Chemistry* **125**, 397–401.
366. *Liu, C., Zhou, H., Su, Y.-C., Li, Y. & Li, J. (2009). Chemical compositions and functional properties of protein isolated from by-product of triangle shell pearl mussel *Hyriopsis* *cumingii*. *Journal of Aquatic Food Product Technology* **18**, 193–208.
367. *Liu, F., Huang, J.-C., Zhou, C., Gao, W., Xia, S., He, S. & Zhou, W. (2019). Development of an algal treatment system for selenium removal: Effects of environmental factors and post-treatment processing of Se-laden algae. *Journal of Hazardous Materials* **365**, 546–554.
368. Liu, F., Li, T., Jia, Z. & Wang, L. (2020). Combination of stiffness, strength, and toughness in 3D printed interlocking nacre-like composites. *Extreme Mechanics Letters* **35**, 100621.
369. *Liu, S., Wei, W., Bai, Z., Wang, X., Li, X., Wang, C., Liu, X., Liu, Y. & Xu, C. (2018). Rapid identification of pearl powder from *Hyriopsis cumingii* by Tri-step infrared spectroscopy combined with computer vision technology. *Spectrochimica Acta - Part A: Molecular and Biomolecular Spectroscopy* **189**, 265–274.
370. *Loganathan, B. G., Kawano, M., Sajwan, K. S. & Owen, D. A. (2001). Extractable organohalogens (EOX) in sediment and mussel tissues from the Kentucky Lake and Kentucky Dam Tailwater, USA. *Toxicological and Environmental Chemistry* **79**, 233–242.
371. *Lopes, A., Lopes-Lima, M., Ferreira, J., Araujo, S., Hinzmann, M., Oliveira, J., Rocha, A., Domingues, B., Bobos, I. & Machado, J. (2014). Biomineralization studies on cellulose membrane exposed to biological fluids of *Anodonta cygnea*. *Journal of Membrane* *Biology* **247**, 501–514.
372. *Louis, F., Rocher, B., Barjhoux, I., Bultelle, F., Dedourge-Geffard, O., Gaillet, V., Bonnard, I., Delahaut, L., Pain-Devin, S., Geffard, A., Paris-Palacios, S. & David, E. (2020). Seasonal monitoring of cellular energy metabolism in a sentinel species, *Dreissena polymorpha* (bivalve): Effect of global change?. *Science of the Total Environment* **725**, 138450.
373. *Lucy, F. E., Connolly, M., Graczyk, T. K., Tamang, L., Sullivan, M. R. & Mastitsky, S. E. (2010). Zebra mussels (*Dreissena polymorpha*) are effective sentinels of water quality irrespective of their size. *Aquatic Invasions* **5**, 49–57.
374. Lucy, F. E., Graczyk, T. K., Tamang, L., Miraflor, A. & Minchin, D. (2008). Biomonitoring of surface and coastal water for *Cryptosporidium*, *Giardia*, and human-virulent microsporidia using molluscan shellfish. *Parasitology Research* **103**, 1369–1375.
375. Lukashev, D. (2008). Assessment of polymetalic pollution of the Dnieper River by the method of calculation of the background content of heavy metals in *Dreissena bugensis*. *Hydrobiological Journal* **44**, 60–75.
376. Lukman, L., Setyobudiandi, I., Muchsin, I. & Hariyadi, S. (2019). Population structure, growth and production of *Corbicula moltkiana* and their relation to cage aquaculture activity in Lake Maninjau, West Sumatra, Indonesia. In *IOP Conference Series: Earth and Environmental Science*, vol. 308, 012077. IOP Publishing.
377. *Lummer, E.-M., Auerswald, K. & Geist, J. (2016). Fine sediment as environmental stressor affecting freshwater mussel behavior and ecosystem services. *Science of the Total Environment* **571**, 1340–1348.
378. *Magar, V. S., Davis, J., Dekker, T., Erickson, M., Matey, D., Patmont, C., Swindoll, M., Brenner, R. & Zeller, C. (2004). Characterization of fate and transport processes: Comparing contaminant recovery with biological endpoint trends. Remediation of Contaminated Sediments. *Proceedings of the Second International Conference on Remediation of Contaminated Sediments*.
379. *Magioncalda, R., Dupuis, C., Blamart, D., Fairon-Demaret, M., Perreau, M., Renard, M., Riveline, J., Roche, M. & Keppens, E. (2001). The palaeocene/eocene isotopic excursion of organic carbon (δ13Corg) in the continental palaeoenvironment at Varangeville (Haute-Normandie, Paris basin) [L'excursion isotopique du carbone organique (δ16Corg) dans les paléoenvironnements continentaux de l'intervalle paleocéne/Eocéne de Varangeville (Haute-Normandie)]. *Bulletin de la Societe Geologique de France* **172**, 349–358.
380. Magni, S., Parolini, M., Soave, C., Marazzi, F., Mezzanotte, V. & Binelli, A. (2015). Removal of metallic elements from real wastewater using zebra mussel bio-filtration process. *Journal of Environmental Chemical Engineering* **3**, 915–921.
381. *Mandal, R. N., Kumar, K., Mohanty, U. L. & Meher, P. K. (2007). Estimation of gut contents of freshwater mussel, *Lamellidens marginalis* L.. *Aquaculture Research* **38**, 1364–1369.
382. Mansfield, R., Williams, A., Hendry, K. & White, K. (2014). Drivers of change in a redeveloped urban lake: long term trends in a simplified system. *Fundamental and Applied Limnology* **185**, 91–105.
383. *Marasinghe Wadige, C. P. M., Taylor, A. M., Maher, W. A. & Krikowa, F. (2014*a*). Bioavailability and toxicity of zinc from contaminated freshwater sediments: Linking exposure-dose-response relationships of the freshwater bivalve *Hyridella australis* to zinc-spiked sediments. *Aquatic Toxicology* **156**, 179–190.
384. *Marasinghe Wadige, C. P. M., Taylor, A. M., Maher, W. A., Ubrihien, R. P. & Krikowa, F. (2014*b*). Effects of lead-spiked sediments on freshwater bivalve, *Hyridella australis*: Linking organism metal exposure-dose-response. *Aquatic Toxicology* **149**, 83–93.
385. *Mărgărit, M. (2016). Testing the endurance of prehistoric adornments: Raw materials from the aquatic environment. *Journal of Archaeological Science* **70**, 66–81.
386. Mărgărit, M. (2020). Personal adornments in the Romanian Eneolithic: Local versus exotic raw materials. *Quaternary International* **539**, 49–61.
387. Mărgărit, M., Mirea, P. & Radu, V. (2018). Exploitation of aquatic resources for adornment and tool processing at Măgura ‘Buduiasca’(‘Boldul lui Moş Ivănuş’) Neolithic settlement (southern Romania). *Quaternary International* **472**, 49–59.
388. *Mariano, B., Cristian, O., Marcela, P. & Porta, A. (2006). Evaluation of a biomarker of Cd(II) exposure on *Limnoperna fortunei*. *Environmental Pollution* **144**, 280–288.
389. *Marlina Radu, S., Kqueen, C. Y., Napis, S., Zakaria, Z., Mutalib, S. A. & Nishibuchi, M. (2007). Detection of TDH and TRH genes in *Vibrio parahaemolyticus* isolated from *Corbicula* *moltkiana* Prime in West Sumatera, Indonesia. *Southeast Asian Journal of Tropical* *Medicine and Public Health* **38**, 349–355.
390. *Martel, P., Kovacs, T., Voss, R. & Megraw, S. (2003). Evaluation of caged freshwater mussels as an alternative method for environmental effects monitoring (EEM) studies. *Environmental Pollution* **124**, 471–483.
391. Martin, P., Hancock, G. J., Johnston, A. & Murray, A. S. (1998). Natural-series radionuclides in traditional north Australian Aboriginal foods. *Journal of Environmental Radioactivity* **40**, 37–58.
392. *Masnado, R. G., Geis, S. W. & Sonzogni, W. C. (1995). Comparative acute toxicity of a synthetic mine effluent to *Ceriodaphnia dubia*, larval fathead minnow and the freshwater mussel *Anodonta imbecilis*. *Environmental Toxicology and Chemistry* **14**, 1913–1920.
393. *Masson, S., Couillard, Y., Campbell, P. G. C., Olsen, C., Pinel-Alloul, B. & Perceval, O. (2010). Responses of two sentinel species (*Hexagenia limbata* - Mayfly; *Pyganodon grandis* - Bivalve) along spatial cadmium gradients in lakes and rivers in northwestern Québec. *Journal of Environmental Monitoring* **12**, 143–158.
394. *Mathai, P. P., Magnone, P., Dunn, H. M. & Sadowsky, M. J. (2020). Water and sediment act as reservoirs for microbial taxa associated with invasive dreissenid mussels. *Science of* *the Total Environment* **703**, 134915.
395. Matter, A., Neubert, E., Preusser, F., Rosenberg, T. & Al-Wagdani, K. (2015). Palaeo-environmental implications derived from lake and sabkha deposits of the southern Rub' al-Khali, Saudi Arabia and Oman. *Quaternary International* **382**, 120–131.
396. *Matthias, U. & Rompp, S. (1994). Evaluation of the *Dreissena*-monitor at the River Rhine–a new biological monitoring-system based on the zebra mussel *Dreissena polymorpha*. *Acta* *Hydrochimica et Hydrobiologica* **22**, 161–165.
397. *Matuszak, P., Grodzicki, G., Jankowski, T. & Matlakiewicz, P. (2015). Biomonitoring of inland and inshore waters with use of *Dreissena polymorpha* mussels. *Polish* *Hyperbaric Research* **52**, 49–53.
398. *McKenzie, J. F. & Ozbay, G. (2009). Viability of a freshwater mussel (*Elliptio complanata*) as a biomechanical filter for aquaculture ponds i: Clearance rate of chlorophyll-α. *Journal* *of Applied Aquaculture* **21**, 205–214.
399. *McKnickle, G. G., Rennie, M. D. & Sprules, W. G. (2006). Changes in benthic invertebrate communities of South Bay, Lake Huron following invasion by zebra mussels (*Dreissena polymorpha*), and potential effects on lake whitefish (*Coregonus* *clupeaformis*) diet and growth. *Journal of Great Lakes Research* **32**, 180–193
400. *McLeester, M. & Schurr, M. (2020). Paleoclimate of the Little Ice Age to the Present in the Kankakee Valley of Illinois and Indiana, USA Based on O-18/O-16 Isotope Ratios of Freshwater Shells. *Environmental Archaeology* **26**, 555–566.
401. *Mellina, E., Rasmussen, J. B. & Mills, E. L. (1995). Impact of zebra mussel (*Dreissena* *polymorpha*) on phosphorus cycling and chlorophyll in lakes. *Canadian Journal of* *Fisheries and Aquatic Sciences* **52**, 2553–2573.
402. *Mersch, J. & Beauvais, M.-N. (1997). The micronucleus assay in the zebra mussel, *Dreissena* *polymorpha*, to *in situ* monitor genotoxicity in freshwater environments. *Mutation* *Research - Genetic Toxicology and Environmental Mutagenesis* **393**, 141–149.
403. *Mersch, J. & Johansson, L. (1993). Transplanted aquatic mosses and freshwater mussels to investigate the trace metal contamination in the rivers meurthe and plaine, France. *Environmental Technology (United Kingdom)* **14**, 1027–1036.
404. *Mersch, J., Morhain, E. & Mouvet, C. (1993). Laboratory accumulation and depuration of copper and cadmium in the freshwater mussel *Dreissena polymorpha* and the aquatic moss *Rhynchostegium riparioides*. *Chemosphere* **27**, 1475–1485.
405. *Mersch, J. & Pihan, J.-C. (1993). Simultaneous assessment of environmental impact on condition and trace metal availability in zebra mussels *Dreissena polymorpha* transplanted into the Wiltz River, Luxembourg. Comparison with the aquatic moss. *Archives of* *Environmental Contamination and Toxicology* **25**, 353–364.
406. *Mersch, J., Wagner, P. & Pihan, J.-C. (1996). Copper in indigenous and transplanted zebra mussels in relation to changing water concentrations and body weight. *Environmental* *Toxicology and Chemistry* **15**, 886–893.
407. *Metcalfe, J. L. & Hayton, A. (1989). Comparison of leeches and mussels as biomonitors for chlorophenol pollution. *Journal of Great Lakes Research* **15**, 654–668.
408. *Metcalfe-Smith, J. L., Green, R. H. & Grapentine, L. C. (1996). Influence of biological factors on concentrations of metals in the tissues of freshwater mussels (*Elliptio complanata* and *Lampsilis radiata radiata*) from the St. Lawrence River. *Canadian Journal of Fisheries* *and Aquatic Sciences* **53**, 205–219.
409. *Metcalfe-Smith, J. L. (1994). Influence of species and sex on metal residues in fresh-water mussels (Family Unionidae) form the St Lawrence River, with implications for biomonitoring programs. *Environmental Toxicology and Chemistry* **13**, 1433–1443.
410. Mezzanotte, V., Marazzi, F., Bissa, M., Pacchioni, S., Binelli, A., Parolini, M., Magni, S., Ruggeri, F. M., Morghen, C. D. G. & Zanotto, C. (2016). Removal of enteric viruses and *Escherichia coli* from municipal treated effluent by zebra mussels. *Science of the Total Environment* **539**, 395–400.
411. Michel, C., Bourgeault, A., Gourlay-Francé, C., Palais, F., Geffard, A. & Vincent-Hubert, F. (2013). Seasonal and PAH impact on DNA strand-break levels in gills of transplanted zebra mussels. *Ecotoxicology and Environmental Safety* **92**, 18–26.
412. Michel, P., Dobson-Waitere, A., Hohaia, H., McEwan, A. & Shanahan, D. F. (2019). The reconnection between mana whenua and urban freshwaters to restore the mouri/life force of the Kaiwharawhara. *New Zealand Journal of Ecology* **43**, 1–10.
413. *Millane, M., Kelly-Quinn, M. & Champ, T. (2008). Impact of the zebra mussel invasion on the ecological integrity of Lough Sheelin, Ireland: Distribution, population characteristics and water quality changes in the lake. *Aquatic Invasions* **3**, 271–281.
414. *Miller, E. J., Tomasic, J. J. & Barnhart, M. C. (2014). A comparison of freshwater mussels (Unionidae) from a late-archaic archeological excavation with recently sampled Verdigris River, Kansas, populations. *American Midland Naturalist* **171**, 16–26.
415. *Mills, E. L., Roseman, E. F., Rutzke, M., Gutenmann, W. H. & Lisk, D. J. (1993). Contaminant and nutrient element levels in soft tissues of zebra and quagga mussels from waters of southern Lake Ontario. *Chemosphere* **27**, 1465–1473.
416. *Miserazzi, A., Sow, M., Gelber, C., Charifi, M., Ciret, P., Dalens, J. M., Weber, C., Le Floch, S., Lacroix, C., Blanc, P. & Massabuau, J. C. (2020). Asiatic clam *Corbicula fluminea* exhibits distinguishable behavioural responses to crude oil under semi-natural multiple stress conditions. *Aquatic Toxicology* **219**, 105381.
417. *Mohamed, A. S., Bin Dajem, S., Al-Kahtani, M., Ali, S. B., Alshehri, M., Shati, A., Morsy, K. & Fahmy, S. R. (2020). Freshwater clam as a potential bioindicator for silver/saponin nanocomposites toxicity. *Bulletin of Environmental Contamination and Toxicology* **105**, 827–834.
418. Montgomery, H. & Barnes, K. (2012). Paleolimnology of uppermost Cretaceous lacustrine deposits in western Texas. *Palaios* **27**, 386–394.
419. *Morey, D. F. & Crothers, G. M. (1998). Clearing up clouded waters: palaeoenvironmental analysis of freshwater mussel assemblages from the Green River shell middens, western Kentucky. *Journal of Archaeological Science* **25**, 907–926.
420. *Moring, J. B. & Rose, D. R. (1997). Occurrence and concentrations of polycyclic aromatic hydrocarbons in semipermeable membrane devices and clams in three urban streams of the Dallas-Fort Worth Metropolitan Area, Texas. *Chemosphere* **34**, 551–566.
421. *Morrison, H., Yankovich, T., Lazar, R. & Haffner, G. D. (1995). Elimination rate constants of 36 PCBs in zebra mussels (*Dreissena polymorpha*) and exposure dynamics in the Lake St Clair Lake Erie corridor. *Canadian Journal of Fisheries and Aquatic Sciences* **52**, 2574–2582.
422. *Mosteo, R., Goñi, P., Miguel, N., Abadías, J., Valero, P. & Ormad, M. P. (2016). Bioaccumulation of pathogenic bacteria and amoeba by zebra mussels and their presence in watercourses. *Environmental Science and Pollution Research* **23**, 1833–1840.
423. Mouabad, A., Fdil, M. A., Maarouf, A. & Pihan, J. (2001). Pumping behaviour and filtration rate of the freshwater mussel *Potomida littoralis* as a tool for rapid detection of water contamination. *Aquatic Ecology* **35**, 51–60.
424. *Mueller, C. R., Eversole, A. G., Turker, H. & Brune, D. E. (2004). Effect of silver cap Hypophthalmichthys molitrix and freshwater mussel *Elliptio complanata* filtration on the phytoplankton community of partitioned aquaculture system units. *Journal of the* *World Aquaculture Society* **35**, 372–382.
425. *Mueting, S. A. & Gerstenberger, S. L. (2010). Mercury concentrations in quagga mussels, *Dreissena bugensis*, from Lakes Mead, Mohave and Havasu. *Bulletin of Environmental* *Contamination and Toxicology* **84**, 497–501.
426. *Mueting, S. A. & Gerstenberger, S. L. (2011). The 100th meridian initiative at the lake mead national recreation area, NV, USA: Differences between boater behaviors before and after a quagga mussel, *Dreissena rostiformis bugensis*, invasion. *Aquatic Invasions* **6**, 223–229.
427. *Nack, C. C., Limburg, K. E. & Schmidt, R. E. (2015). Diet composition and feeding behavior of larval American Shad, *Alosa sapidissima* (Wilson), after the introduction of the invasive Zebra Mussel, *Dreissena polymorpha* (Pallas), in the Hudson River Estuary, NY. *Northeastern Naturalist* **22**, 437–450.
428. *Naimo, T. J., Atchison, G. J. & Holland‐Bartels, L. E. (1992*a*). Sublethal effects of cadmium on physiological responses in the pocketbook mussel, *Lampsilis ventricosa*. *Environmental Toxicology and Chemistry* **11**, 1013–1021.
429. *Naimo, T. J., Waller, D. L. & Holland-Bartels, L. E. (1992*b*). Heavy metals in the threeridge mussel *Amblema plicata plicata* (Say, 1817) in the upper Mississippi River. *Journal of* *Freshwater Ecology* **7**, 209–217.
430. *Navarro, A., Faria, M., Barata, C. & Pina, B. (2011). Transcriptional response of stress genes to metal exposure in zebra mussel larvae and adults. *Environmental Pollution* **159**, 100–107.
431. *Negri, A. P. & Jones, G. J. (1995). Bioaccumulation of paralytic shellfish poisoning (PSP) toxins from the cyanobacterium *Anabaena circinalis* by the freshwater mussel *Alathyria* *condola*. *Toxicon* **33**, 667–678.
432. *Netpae, T. & Phalaraksh, C. (2009). Water quality and heavy metal monitoring in water, sediments, and tissues of *Corbicula* sp from Bung Boraphet Reservoir, Thailand. *Chiang Mai Journal of Science* **36**, 395–402.
433. *Neufeld, D. S. G. (2010). Mercury accumulation in caged *Corbicula*: Rate of uptake and seasonal variation. *Environmental Monitoring and Assessment* **168**, 385–396.
434. *Neves, R. J. (1999). Biological feasibility of freshwater mussel and pearl culture in gulf coast states. *Gulf of Mexico Science* **17**, 103–108.
435. Ngor, P. B., Sor, R., Prak, L. H., So, N., Hogan, Z. S. & Lek, S. (2018). Mollusc fisheries and length–weight relationship in Tonle Sap flood pulse system, Cambodia. *Annales de Limnologie-International Journal of Limnology* **54**, 34.
436. *Nicklin, L. & Balas, M. T. (2007). Correlation between unionid mussel density and EPA habitat-assessment parameters. *Northeastern Naturalist* **14**, 225–234.
437. Nicodemus, A. (2011). The Bronze Age and Dacian fauna from new excavations at Pecica Şanţul Mare. *Analele Banatului, Serie Nouă* **19**, 79–84.
438. *Nielsen, J. K., Helama, S. & Nielsen, J. K. (2008). Taphonomy of freshwater molluscs in carbonate-poor deposits: A case study of the river pearl mussel in northeastern Finnish Lapland. *Norsk Geologisk Tidsskrift* **88**, 103–116.
439. Noda, Y., Zhao, Y., Jin, X. & Li, Y. (2007). Molluscan remains from the Liangzhu archaeological sites of Zhejiang Province, China. *Memoir of the Fukui Prefectural Dinosaur Museum* **6**, 45–55.
440. *Nogaro, G. & Steinman, A. D. (2014). Influence of ecosystem engineers on ecosystem processes is mediated by lake sediment properties. *Oikos* **123**, 500–512.
441. O'Neil, D. D. & Gillikin, D. P. (2014). Do freshwater mussel shells record road-salt pollution? *Scientific Reports* **4**, 7168.
442. *Oliveira, L. F. D., Cabral, M. T., Vieira, C. E. D., Antoniazzi, M. H., Risso, W. E. & Martinez, C. B. D. R. (2016). Metals bioaccumulation and biomarkers responses in the Neotropical freshwater clam *Anodontites trapesialis*: Implications for monitoring coal mining areas. *Science of the Total Environment* **571**, 983–991.
443. *Oliveira, P., Barboza, L. G. A., Branco, V., Figueiredo, N., Carvalho, C. & Guilhermino, L. (2018). Effects of microplastics and mercury in the freshwater bivalve *Corbicula fluminea* (Müller, 1774): Filtration rate, biochemical biomarkers and mercury bioconcentration. *Ecotoxicology and Environmental Safety* **164**, 155–163.
444. *Osman, A. M., Van Den Heuvel, H. & Van Noort, P. C. M. (2007). Differential responses of biomarkers in tissues of a freshwater mussel, *Dreissena polymorpha*, to the exposure of sediment extracts with different levels of contamination. *Journal of Applied Toxicology* **27**, 51–59.
445. *Othman, F., Islam, M. S., Sharifah, E. N., Shahrom-Harrison, F. & Hassan, A. (2015). Biological control of streptococcal infection in Nile tilapia *Oreochromis niloticus* (Linnaeus, 1758) using filter-feeding bivalve mussel *Pilsbryoconcha exilis* (Lea, 1838). *Journal of* *Applied Ichthyology* **31**, 724–728.
446. *Outa, J. O., Kowenje, C. O., Avenant-Oldewage, A. & Jirsa, F. (2020). Trace elements in crustaceans, mollusks and fish in the Kenyan part of Lake Victoria: bioaccumulation, bioindication and health risk analysis. *Archives of Environmental Contamination* *and Toxicology* **78**, 589–603.
447. Ozersky, T., Evans, D. O. & Ginn, B. K. (2015). Invasive mussels modify the cycling, storage and distribution of nutrients and carbon in a large lake. *Freshwater Biology* **60**, 827–843.
448. Ozersky, T., Malkin, S. Y., Barton, D. R. & Hecky, R. E. (2009). Dreissenid phosphorus excretion can sustain *C. glomerata* growth along a portion of Lake Ontario shoreline. *Journal of Great Lakes Research* **35**, 321–328.
449. *Pain-Devin, S., Cossu-Leguille, C., Geffard, A., Giambérini, L., Jouenne, T., Minguez, L., Naudin, B., Parant, M., Rodius, F., Rousselle, P., Tarnowska, K., Daguin-Thiébaut, C., Viard, F. & Devin, S. (2014). Towards a better understanding of biomarker response in field survey: A case study in eight populations of zebra mussels. *Aquatic Toxicology* **155**, 52–61.
450. *Paller, M. H., Jagoe, C. H., Bennett, H., Brant, H. A. & Bowers, J. A. (2004). Influence of methylmercury from tributary streams on mercury levels in Savannah River Asiatic clams. *Science of the Total Environment* **325**, 209–219.
451. *Palos Ladeiro, M., Aubert, D., Villena, I., Geffard, A. & Bigot, A. (2014). Bioaccumulation of human waterborne protozoa by zebra mussel (*Dreissena polymorpha*): Interest for water biomonitoring. *Water Research* **48**, 148–155.
452. *Palos Ladeiro, M., Bigot-Clivot, A., Aubert, D., Villena, I. & Geffard, A. (2015). Assessment of *Toxoplasma gondii* levels in zebra mussel (*Dreissena polymorpha*) by real-time PCR: an organotropism study. *Environmental Science and Pollution Research* **22**, 13693–13701.
453. *Pandey, A. & Singh, A. (2015). Effect of different pearl nuclei implantation and rearing methods on survival, growth and pearl formation in freshwater mussel, *Lamellidens marginalis* in Punjab. *Ecology, Environment and Conservation* **21**, AS331–AS335.
454. Parada, E., Peredo, S., Cardenas, S., Valdebenito, I. & Peredo, M. (2008). *Diplodon chilensis* Gray, 1828 (Bivalvia: Hyriidae) a potential residual waters depurator on inland water salmonid, fish-farms: A laboratory scale study. *Gayana (Concepción)* **72**, 68–78.
455. *Parant, M. & Pain, S. (2001). Potential use of multixenobiotic defense mechanism (mxdm) in *Dreissena polymorpha* as a biomarker for the monitoring of freshwater pollution. *Water* *Research* **35**, 3743–3748.
456. Park, H., Yokoyama, A. & Okino, T. (2001). Fate of microcystin in Lake Suwa. *Japanese Journal of Limnology* **62**, 229–248.
457. *Patrick, C. H., Waters, M. N. & Golladay, S. W. (2017). The distribution and ecological role of *Corbicula fluminea* (Müller, 1774) in a large and shallow reservoir. *BioInvasions* *Records* **6**, 39–48.
458. *Peacock, E. & Jenkins, C. (2010). The distribution and research value of archaeological mussel shell: An overview from Mississippi. *Midcontinental Journal of Archaeology* **35**, 91–116.
459. *Peacock, E. & Seltzer, J. L. (2008). A comparison of multiple proxy data sets for paleoenvironmental conditions as derived from freshwater bivalve (Unionid) shell. *Journal of Archaeological Science* **35**, 2557–2565.
460. *Pellacani, C., Buschini, A., Furlini, M., Poli, P. & Rossi, C. (2006). A battery of *in vivo* and *in* *vitro* tests useful for genotoxic pollutant detection in surface waters. *Aquatic* *Toxicology* **77**, 1–10.
461. *Peltier, G. L., Meyer, J. L., Jagoe, C. H. & Hopkins, W. A. (2008). Using trace element concentrations in *Corbicula fluminea* to identify potential sources of contamination in an urban river. *Environmental Pollution* **154**, 283–290.
462. Peng, Y. C., Subeq, Y. M., Tien, C. C. & Lee, R. P. (2017). Freshwater clam extract supplementation improves wound healing by decreasing the tumor necrosis factor α level in blood. *Journal of the Science of Food and Agriculture* **97**, 1193–1199.
463. *Penkman, K. E. H., Preece, R. C., Keen, D. H., Maddy, D., Schreve, D. C. & Collins, M. J. (2007). Testing the aminostratigraphy of fluvial archives: the evidence from intra-crystalline proteins within freshwater shells. *Quaternary Science Reviews* **26**, 2958–2969.
464. *Perceval, O., Couillard, Y., Pinel-Alloul, B., Bonneris, E. & Campbell, P. G. C. (2006). Long-term trends in accumulated metals (Cd, Cu and Zn) and metallothionein in bivalves from lakes within a smelter-impacted region. *Science of the Total Environment* **369**, 403–418.
465. *Pereira, W. E., Domagalski, J. L., Hostettler, F. D., Brown, L. R. & Rapp, J. B. (1996). Occurrence and accumulation of pesticides and organic contaminants in river sediment, water and clam tissues from the San Joaquin River and tributaries, California. *Environmental* *Toxicology and Chemistry* **15**, 172–180.
466. *Pereto, C., Coynel, A., Lerat-Hardy, A., Gourves, P.-Y., Schäfer, J. & Baudrimont, M. (2020). *Corbicula fluminea*: A sentinel species for urban Rare Earth Element origin. *Science of* *the Total Environment* **732**, 138552.
467. Pérez, A. E., Batres, D. A., Rocchetta, I., Eppis, M. R., Bianchi, M. L. & Luquet, C. M. (2020). Paleoenvironmental reconstruction using stable isotopes and trace elements from archaeological freshwater bivalve shell fragments in Northwest Patagonia, Argentina. *Quaternary International* **547**, 22–32.
468. *Peterson, M. J., Southworth, G. R. & Ham, K. D. (1994). Effect of sublethal chlorinated discharges on PCB accumulation in transplanted Asiatic clams (*Corbicula fluminea*). *Water, Air,* *& Soil Pollution* **73**, 169–178.
469. *Pham, T. L. (2020). Accumulation, depuration and risk assessment of cadmium (Cd) and lead (Pb) in clam (*Corbicula fluminea*) (O. F. Muller, 1774) under laboratory conditions. *Iranian Journal of Fisheries Sciences* **19**, 1062–1072.
470. Pham, T.-L., Shimizu, K., Kanazawa, A., Gao, Y., Dao, T.-S. & Utsumi, M. (2016). Microcystin accumulation and biochemical responses in the edible clam *Corbicula leana* P. exposed to cyanobacterial crude extract. *Journal of Environmental Sciences* **44**, 120–130.
471. Pierce, H. G. & Constenius, K. (2001). Late Eocene-Oligocene nonmarine mollusks of the Northern Kishenehn Basin, Montana and British Columbia. *Annals of Carnegie Museum* **70**, 1–112.
472. Pipolo, M., Martins, R. C., Quinta-Ferreira, R. M. & Costa, R. (2017). Integrating the Fenton's process with biofiltration by *Corbicula fluminea* to reduce chemical oxygen demand of winery effluents. *Journal of Environmental Quality* **46**, 436–442.
473. *Pizarro, H., Di Fiori, E., Sinistro, R., Ramírez, M., Rodríguez, P., Vinocur, A. & Cataldo, D. (2016). Impact of multiple anthropogenic stressors on freshwater: how do glyphosate and the invasive mussel *Limnoperna fortunei* affect microbial communities and water quality?. *Ecotoxicology* **25**, 56–68.
474. *Potet, M., Giambérini, L., Pain-Devin, S., Louis, F., Bertrand, C. & Devin, S. (2018). Differential tolerance to nickel between *Dreissena polymorpha* and *Dreissena rostriformis bugensis* populations. *Scientific Reports* **8**, 700.
475. *Pourang, N., Richardson, C. A. & Mortazavi, M. S. (2010). Heavy metal concentrations in the soft tissues of swan mussel (*Anodonta cygnea*) and surficial sediments from Anzali wetland, Iran. *Environmental monitoring and assessment* **163**, 195–213.
476. *Pradhan, S., Saurabh, S., Padhi, N., Kumar, T., Kumar, R., Mohanty, U. L. & Sundaray, J. K. (2020). Length-weight, width-weight and height-weight relationships of cultured freshwater pearl mussel, *Lamellidens marginalis* (Lamarck, 1819). *Indian Journal of Fisheries* **67**, 157–160.
477. *Pugsley, C. W., Hebert, P. D. N. & McQuarrie, P. M. (1988). Distribution of contaminants in clams and sediments from the Huron-Erie corridor. II–Lead and Cadmium. *Journal of Great Lakes Research* **14**, 356–368.
478. *Putri, S. R., Anjani, G., Wijayanti, H. S. & Nuryanto (2018). Freshwater Clams (*Pilsbryoconcha exilis*) as an Potential Local Mineral Sources in Weaning Food to Overcome Stunting in Grobogan, Central Java, Indonesia. *IOP Conference Series: Earth and Environmental Science* **116**, 012077.
479. Qiao, D., He, X., Wei, C., Xia, L. & Bao, L. (2016). Effects of *Hyriopsis cumingii* polysaccharides on mice immunologic receptor, transcription factor, and cytokine. *Journal of Food Science* **81**, H1288–H1294.
480. Raikow, D. F., Sarnelle, O., Wilson, A. E. & Hamilton, S. K. (2004). Dominance of the noxious cyanobacterium *Microcystis aeruginosa* in low‐nutrient lakes is associated with exotic zebra mussels. *Limnology and Oceanography* **49**, 482–487.
481. Rak, A. E., Azizan, A. T., Yaacob, M. R., Hamzah, Z., Omar, S. A. S., Zakaria, M. N., Ismail, M., Ibrahim, W. K. W., Rani, W. S. F. M. & Zaki, M. Z. (2020*a*). Traditional processing method of smoked *Corbicula fluminea* (Etak): case of Etak vendor in Kelantan, Malaysia. *IOP Conference Series: Earth and Environmental Science* **596**, 012057.
482. *Rak, A. E., Nasir, S. N. A. M., Nor, M. M., Han, D. K., Appalasamy, S., Abdullah, F. & Ghazi, R. M. (2020*b*). Proximate analysis and fatty acid of *Corbicula fluminea* (*C. fluminea*) tissue in Kelantan, Malaysia. *Environmental Science and Pollution Research* **27**, 24772–24785.
483. *Ramesha, M. M. & Sophia, S. (2015). Morphometry, length-weight relationships and condition index of *Parreysia favidens* (Benson, 1862) (Bivalvia: Unionidae) from River Seeta in the Western Ghats, India. *Indian Journal of Fisheries* **62**, 18–24.
484. *Randklev, C. R., Wolverton, S. & Kennedy, J. H. (2009). A biometric technique for assessing prehistoric freshwater mussel population dynamics (family: Unionidae) in north Texas. *Journal of Archaeological Science* **36**, 205–213.
485. *Ravera, O., Beone, G. M., Cenci, R. & Lodigiani, P. (2003). Metal concentrations in *Unio pictorum* *mancus* (Mollusca, Lamellibranchia) from of 12 Northern Italian lakes in relation to their trophic level. *Journal of Limnology* **62**, 121–138.
486. *Reeders, H. H. & Bij de Vaate, A. (1990). Zebra mussels (*Dreissena polymorpha*): a new perspective for water quality management. *Hydrobiologia* **200–201**, 437–450.
487. *Reeders, H. H. & Bij de Vaate, A. (1992). Bioprocessing of polluted suspended matter from the water column by the zebra mussel (*Dreissena polymorpha* Pallas). *Hydrobiologia* **239**, 53–63.
488. *Reeders, H. H., De Vaate, A. B. & Slim, F. J. (1989). The fitration rate of *Dreissena* *polymorpha* (Bivalvia) in three Dutch lakes with reference to biological water quality management. *Freshwater Biology* **22**, 133–141.
489. Regoli, L., Chan, H. M., de Lafontaine, Y. & Mikaelian, I. (2001). Organotins in zebra mussels (*Dreissena polymorpha*) and sediments of the Quebec City Harbour area of the St. Lawrence River. *Aquatic Toxicology* **53**, 115–126.
490. Reis, P. A., Guilhermino, L., Antunes, C. & Sousa, R. G. (2014). Assessment of ecological quality of the Minho estuary (Northwest Iberian Peninsula) based on metal concentrations in sediments and in *Corbicula fluminea*. *Limnetica* **33**, 161–174.
491. *Ren, J. Y., Sha, W. Q., Shang, S. M. & Yuan, E. D. (2020). Hepatoprotective peptides purified from *Corbicula* *fluminea* and its effect against ethanol-induced LO2 cells injury. *International* *Journal of Food Science and Technology* **56**, 352–361.
492. *Renaud, C. B., Kaiser, K. L. E, Comba, M. E. & Metcalfe-Smith, J. L. (1995). Comparison between lamprey ammocoetes and bivalve mollusks as biomonitors of organochlorine contaminants. *Canadian Journal of Fisheries and Aquatic Sciences* **52**, 276–282.
493. *Ricciardi, F., Binelli, A. & Provini, A. (2006). Use of two biomarkers (CYP450 and acetylcholinesterase) in zebra mussel for the biomonitoring of Lake Maggiore (northern Italy). *Ecotoxicology and Environmental Safety* **63**, 406–412.
494. *Rice, C. P. & White, D. S. (1987). PCB availability assessment of river dredging using caged clams and fish. *Environmental Toxicology and Chemistry* **6**, 259–274.
495. Richman, L. A., Hobson, G., Williams, D. J. & Reiner, E. (2011). The Niagara River mussel biomonitoring program (*Elliptio complanata*): 1983–(2009). *Journal of Great Lakes Research* **37**, 213–225.
496. *Richman, L. A. & Somers, K. (2005). Can we use zebra and quagga mussels for biomonitoring contaminants in the Niagara River?. *Water, Air, and Soil Pollution* **167**, 155–178.
497. Richman, L. A. & Somers, K. (2010). Monitoring metal and persistent organic contaminant trends through time using quagga mussels (*Dreissena bugensis*) collected from the Niagara River. *Journal of Great Lakes Research* **36**, 28–36.
498. *Richter, A. F. (1986). Biomanipulation and its feasibility for water quality management in shallow eutrophic water bodies in The Netherlands. *Hydrobiological Bulletin* **20**, 165–172.
499. *Ricken, W., Steuber, T., Freitag, H., Hirschfeld, M. & Niedenzu, B. (2003). Recent and historical discharge of a large European river system - Oxygen isotopic composition of river water and skeletal aragonite of Unionidae in the Rhine. *Palaeogeography,* *Palaeoclimatology, Palaeoecology* **193**, 73–86.
500. *Ridout-Sharpe, J. (2015). Changing lifestyles in the northern Levant: Late Epipalaeolithic and early Neolithic shells from Tell Abu Hureyra. *Quaternary International* **390**, 102–116.
501. *Rigonato, J., Mantovani, M. S. & Jordão, B. Q. (2005). Comet assay comparison of different *Corbicula fluminea* (Mollusca) tissues for the detection of genotoxicity. *Genetics and* *Molecular Biology* **28**, 464–468.
502. *Rigonato, J., Mantovani, M. S. & Jordão, B. Q. (2010). Detection of genotoxicity of water from an urbanized stream, in *Corbicula fluminea* (Mollusca) (*in vivo*) and CHO-K1 cells (*in* *vitro*) using comet assay. *Archives of Environmental Contamination and Toxicology* **59**, 31–38.
503. Riva, C., Binelli, A., Parolini, M. & Provini, A. (2010). The case of pollution of Lake Maggiore: a 12-year study with the bioindicator mussel *Dreissena polymorpha*. *Water, Air, & Soil Pollution* **210**, 75–86.
504. *Roche, H., Vollaire, Y., Martin, E., Rouer, C., Coulet, E., Grillas, P. & Banas, D. (2009). Rice fields regulate organochlorine pesticides and PCBs in lagoons of the Nature Reserve of Camargue. *Chemosphere* **75**, 526–533.
505. *Roditi, H. A. & Fisher, N. S. (1999). Rates and routes of trace element uptake in zebra mussels. *Limnology and Oceanography* **44**, 1730–1749.
506. *Roditi, H. A., Fisher, N. S. & Sañudo-Wilhelmy, S. A. (2000). Field testing a metal bioaccumulation model for zebra mussels. *Environmental Science and Technology* **34**, 2817–2825.
507. *Roditi, H. A., Strayer, D. L. & Findlay, S. E. G. (1997). Characteristics of zebra mussel (*Dreissena* *polymorpha*) biodeposits in a tidal freshwater estuary. *Archiv fur Hydrobiologie* **140**, 207–219.
508. *Roe, S. L. & MacIsaac, H. J. (1998). Temporal variation of organochlorine contaminants in the zebra mussel *Dreissena polymorpha* in Lake Erie. *Aquatic Toxicology* **41**, 125–140.
509. Romanus, K., Van Neer, W., Marinova, E., Verbeke, K., Luypaerts, A., Accardo, S., Hermans, I., Jacobs, P., De Vos, D. & Waelkens, M. (2008). Brassicaceae seed oil identified as illuminant in Nilotic shells from a first millennium AD Coptic church in Bawit, Egypt. *Analytical and Bioanalytical Chemistry* **390**, 783–793.
510. *Rousseau, A., Escotte-Binet, S., La Carbona, S., Dumètre, A., Chagneau, S., Favennec, L., Kubina, S., Dubey, J. P., Majou, D., Bigot-Clivot, A., Villena, I. & Aubert, D. (2019). *Toxoplasma gondii* oocyst infectivity assessed using a sporocystbased cell culture assay combined with quantitative PCR for environmental applications. *Applied and* *Environmental Microbiology* **85**, e01189–19.
511. Rosa, I. C., Costa, R., Gonçalves, F. & Pereira, J. L. (2014). Bioremediation of metal-rich effluents: could the invasive bivalve *Corbicula fluminea* work as a biofilter? *Journal of Environmental Quality* **43**, 1536–1545.
512. Roy, E. D., Martin, J. F., Irwin, E. G., Conroy, J. D. & Culver, D. A. (2010). Transient social–ecological stability: the effects of invasive species and ecosystem restoration on nutrient management compromise in Lake Erie. *Ecology and Society* **15**, 20.
513. *Russell, R. W. & Gobas, F. A. P. C. (1989). Calibration of the freshwater mussel, *Elliptio* *complanata*, for quantitative biomonitoring of hexachlorobenzene and octachlorostyrene in aquatic systems. *Bulletin of Environmental Contamination and Toxicology* **43**, 576–582.
514. *Ryan, B., Bollhöfer, A. & Martin, P. (2008). Radionuclides and metals in freshwater mussels of the upper South Alligator River, Australia. *Journal of Environmental Radioactivity* **99**, 509–526.
515. *Saitoh, M., Kimura, H., Kozawa, K., Nishio, O. & Shoji, A. (2007). Detection and phylogenetic analysis of norovirus in *Corbicula fluminea* in a freshwater river in Japan. *Microbiology* *and Immunology* **51**, 815–822.
516. Sakalauskaite, J., Andersen, S. H., Biagi, P., Borrello, M. A., Cocquerez, T., Colonese, A. C., Dal Bello, F., Girod, A., Heumüller, M. & Koon, H. (2019). 'Palaeoshellomics’ reveals the use of freshwater mother-of-pearl in prehistory. *Elife* **8**, e45644.
517. *Salánki, J., V.-Balogh, K. & Berta, E. (1982). Heavy metals in animals of Lake Balaton. *Water* *Research* **16**, 1147–1152.
518. *Salgueiro-González, N., Turnes-Carou, I., Besada, V., Muniategui-Lorenzo, S., López-Mahía, P. & Prada-Rodríguez, D. (2015). Occurrence, distribution and bioaccumulation of endocrine disrupting compounds in water, sediment and biota samples from a European river basin. *Science of the Total Environment* **529**, 121–130.
519. Sanko, A., Gaigalas, A. & Yelovicheva, Y. (2011). Paleoclimatic and stratigraphic significance of *Belgrandia marginata* (Michaud) in Late Quaternary malacofauna of Belarus and Lithuania. *Quaternary International* **241**, 68–78.
520. *Santos, H. M., Diniz, M. S., Costa, P. M., Peres, I., Costa, M. H., Alves, S. & Capelo, J. L. (2007). Toxicological effects and bioaccumulation in the freshwater clam (*Corbicula fluminea*) following exposure to trivalent arsenic. *Environmental Toxicology* **22**, 502–509.
521. *Sapone, A., Canistro, D., Vivarelli, F. & Paolini, M. (2016). Perturbation of xenobiotic metabolism in *Dreissena polymorpha* model exposed *in situ* to surface water (Lake Trasimene) purified with various disinfectants. *Chemosphere* **144**, 548–554.
522. *Sarikhani, I. & Javanshir, A. (2010). Evaluation of bivalve (*Anodonta cygnea*) in filtration of nitrogen and phosphorus compounds. *Journal of Environmental Studies* **36**, 119–126.
523. *Sarnelle, O., White, J. D., Horst, G. P. & Hamilton, S. K. (2012). Phosphorus addition reverses the positive effect of zebra mussels (*Dreissena polymorpha*) on the toxic cyanobacterium, *Microcystis aeruginosa*. *Water Research* **46**, 3471–3478.
524. *Schäfer, S., Hamer, B., Treursić, B., Möhlenkamp, C., Spira, D., Korlević, M., Reifferscheid, G. & Claus, E. (2012). Comparison of bioaccumulation and biomarker responses in *Dreissena* *polymorpha* and *D. bugensis* after exposure to resuspended sediments. *Archives of* *Environmental Contamination and Toxicology* **62**, 614–627.
525. *Schaller, J. & Planer-Friedrich, B. (2017). The filter feeder *Dreissena polymorpha* affects nutrient, silicon, and metal(loid) mobilization from freshwater sediments. *Chemosphere* **174**, 531–537.
526. *Schernewski, G., Friedland, R., Buer, A.-L., Dahlke, S., Drews, B., Höft, S., Klumpe, T., Schadach, M., Schumacher, J. & Zaiko, A. (2019). Ecological-social-economic assessment of zebra-mussel cultivation scenarios for the Oder (Szczecin) Lagoon. *Journal of Coastal* *Conservation* **23**, 913–929.
527. Schernewski, G., Stybel, N. & Neumann, T. (2012). Zebra mussel farming in the Szczecin (Oder) Lagoon: water-quality objectives and cost-effectiveness. *Ecology and Society* **17**, 4.
528. *Schmitt, N., Marin, F., Thomas, J., Plasseraud, L. & Demoy-Schneider, M. (2018). Pearl grafting: Tracking the biological origin of nuclei by straightforward immunological methods. *Aquaculture Research* **49**, 692–700.
529. *Schöll-Barna, G. (2011). An isotope mass balance model for the correlation of freshwater bivalve shell (*Unio pictorum*) carbonate δ18O to climatic conditions and water δ18O in Lake Balaton (Hungary). *Journal of Limnology* **70,** 272–282.
530. Schöll-Barna, G., Demény, A., Serlegi, G., Fábián, S., Sümegi, P., Fórizs, I. & Bajnóczi, B. (2012). Climatic variability in the Late Copper Age: stable isotope fluctuation of prehistoric *Unio pictorum* (Unionidae) shells from Lake Balaton (Hungary). *Journal of Paleolimnology* **47**, 87–100.
531. Schöne, B. R., Meret, A. E., Baier, S. M., Fiebig, J., Esper, J., McDonnell, J. & Pfister, L. (2020). Freshwater pearl mussels from northern Sweden serve as long-term, high-resolution stream water isotope recorders. *Hydrology and Earth System Sciences* **24**, 673–696.
532. *Sebe, K., Selmeczi, I., Szuromi-Korez, A., Hably, L., Kovacs, A. & Benko, Z. (2019). Miocene syn-rift lacustrine sediments in the Mecsek Mts. (SW Hungary). *Swiss Journal of Geosciences* **112**, 83–100.
533. *Sebesvari, Z., Friederike Ettwig, K. & Emons, H. (2005). Biomonitoring of tin and arsenic in different compartments of a limnic ecosystem with emphasis on *Corbicula fluminea* and *Dikerogammarus villosus*. *Journal of Environmental Monitoring* **7**, 203–207.
534. *Seker, E., Sarieyyupoglu, M. & Cetinkaya, B. (2003). Identification of *Salmonella* isolated from freshwater mussels (*Unio elongatulus eucirrus* Bourguignat, 1860) by polymerase chain reaction. *Turkish Journal of Veterinary & Animal Sciences* **27**, 201–206.
535. Selegean, J. P. W., Kusserow, R., Patel, R., Heidtke, T. M. & Ram, J. L. (2001). Using zebra mussels to monitor *Escherichia coli* in environmental waters. *Journal of Environmental Quality* **30**, 171–179.
536. *Shchelinsky, V. E., Gurova, M., Tesakov, A. S., Titov, V. V., Frolov, P. D. & Simakova, A. N. (2016). The Early Pleistocene site of Kermek in western Ciscaucasia (southern Russia): Stratigraphy, biotic record and lithic industry (preliminary results). *Quaternary* *International* **393**, 51–69.
537. *Shen, R., Gu, X., Chen, H., Mao, Z., Zeng, Q. & Jeppesen, E. (2020). Combining bivalve (*Corbicula* *fluminea*) and filter-feeding fish (*Aristichthys nobilis*) enhances the bioremediation effect of algae: An outdoor mesocosm study. *Science of the Total Environment* **727**, 138692.
538. *Shoults-Wilson, W. A., Elsayed, N., Leckrone, K. & Unrine, J. (2015). Zebra mussels (*Dreissena* *polymorpha*) as a biomonitor of trace elements along the southern shoreline of Lake Michigan. *Environmental Toxicology and Chemistry* **34**, 412–419.
539. *Shoults-Wilson, W. A., Peterson, J. T., Unrine, J. M., Rickard, J. & Black, M. C. (2009). The Asian clam *Corbicula fluminea* as a biomonitor of trace element contamination: Accounting for different sources of variation using an hierarchical linear model. *Environmental* *Toxicology and Chemistry* **28**, 2224–2232.
540. Sicuro, B., Castelar, B., Mugetti, D., Pastorino, P., Chiarandon, A., Menconi, V., Galloni, M. & Prearo, M. (2020). Bioremediation with freshwater bivalves: A sustainable approach to reducing the environmental impact of inland trout farms. *Journal of Environmental Management* **276**, 111327.
541. *Silantiev, V., Urazaeva, M. N. & Valeriy, G. (2018). The nonmarine bivalve *Permianaia* gen. nov., the last member of Naiaditidae from the terminal Permian the east European platform. *Paleontological Journal* **52**, 777–790.
542. Silva, C., Anselmo, A., Macário, I. P., de Figueiredo, D., Gonçalves, F. J. & Pereira, J. L. (2020). The bad against the villain: Suitability of *Corbicula fluminea* as a bioremediation agent towards cyanobacterial blooms. *Ecological Engineering* **152**, 105881.
543. *Silva, V., Abrantes, N., Costa, R., Keizer, J. J., Gonçalves, F. & Pereira, J. L. (2016). Effects of ash-loaded post-fire runoff on the freshwater clam *Corbicula fluminea*. *Ecological* *Engineering* **90**, 180–189.
544. *Silva, F. A. E. & Giani, A. (2018). Population dynamic of bloom-forming *Microcystis* *aeruginosa* in the presence of the invasive bivalve *Limnoperna* *fortunei*. *Harmful Algae* **73**, 148–156.
545. *Silverman, H., Lynn, J. W., Achberger, E. C. & Dietz, T. H. (1996). Gill structure in zebra mussels: Bacterial-sized particle filtration. *American Zoologist* **36**, 373–384.
546. *Słodkowicz-Kowalska, A., Majewska, A. C., Rzymski, P., Skrzypczak, T. & Werner, A. (2015). Human waterborne protozoan parasites in freshwater bivalves (*Anodonta anatina* and *Unio tumidus*) as potential indicators of fecal pollution in urban reservoir. *Limnologica* **51**, 32–36.
547. *Smith, R. M. H, Sidor, C. A., Angielczyk, K. D., Nesbitt, S. J. & Tabor, N. J. (2017). Taphonomy and paleoenvironments of Middle Triassic bone accumulations in the Lifua member of the Manda Beds, Songea Group (Ruhuhu basin), Tanzania. *Journal of Vertebrate* *Paleontology* **37**, 65–79.
548. *Smolders, R., Bervoets, L. & Blust, R. (2002). Transplanted zebra mussels (*Dreissena* *polymorpha*) as active biomonitors in an effluent-dominated river. *Environmental* *Toxicology and Chemistry* **21**, 1889–1896.
549. *Smolders, R., Bervoets, L. & Blust, R. (2004). *In situ* and laboratory bioassays to evaluate the impact of effluent discharges on receiving aquatic ecosystems. *Environmental* *Pollution* **132**, 231–243.
550. *Sohail, M., Khan, M. N., Chaudhry, A. S. & Qureshi, N. A. (2016). Bioaccumulation of heavy metals and analysis of mineral element alongside proximate composition in foot, gills and mantle of freshwater mussels (*Anodonta anatina*). *Rendiconti Lincei* **27**, 687–696.
551. *Sohail, M., Khan, M. N., Qureshi, N. A. & Chaudhry, A. S. (2017). Monitoring DNA damage in gills of freshwater mussels (*Anodonta anatina*) exposed to heavy metals. *Pakistan Journal* *of Zoology* **49**, 305–311.
552. *Song, H.-L., Li, X.-N., Wang, X.-J. & Lu, X.-W. (2011). Enhancing nitrogen removal performance of vegetated floating-bed by adding *Hyriopsis cumingii* Lea and an artificial medium. *Fresenius Environmental Bulletin* **20**, 2435–2441.
553. *Song, H., Li, X., Li, W. & Lu, X. (2014). Role of biologic components in a novel floating-bed combining Ipomoea aquatic, *Corbicula fluminea* and biofilm carrier media. *Frontiers* *of Environmental Science and Engineering* **8**, 215–225.
554. *Song, E. J., Chan, M. W. Y, Shin, J. W. & Chen, C. C. (2017). Hard clam extracts induce atypical apoptosis in human gastric cancer cells. *Experimental and Therapeutic Medicine* **14**, 1409–1418.
555. *Song, Y., Sun, B., Gao, Y. & Yi, H. (2019). The environment and subsistence in the lower reaches of the Yellow River around 10,000 BP - faunal evidence from the bianbiandong cave site in Shandong Province, China. *Quaternary International* **521**, 35–43.
556. Sonowal, J. & Kardong, D. (2020). Nutritional evaluation of freshwater bivalve, *Lamellidens* spp. from the upper Brahmaputra basin, Assam with special reference to dietary essential amino acids, omega fatty acids and minerals. *Journal of Environmental Biology* **41**, 931–941.
557. Sorontou, Y. & Agussalim. (2016). Effectiveness lime shells of *Anodonta anatina* for larvae of *Anopheles* and *Aedes aegypti* mosquitoes in Jayapura District, Indonesia. *Research Journal of Pharmaceutical, Biological and Chemical Sciences* **7**, 3180–3186.
558. *Soto, D. X., Roig, R., Gacia, E. & Catalan, J. (2011). Differential accumulation of mercury and other trace metals in the food web components of a reservoir impacted by a chlor-alkali plant (Flix, Ebro River, Spain): Implications for biomonitoring. *Environmental* *Pollution* **159**, 1481–1489.
559. *Stäb, J. A., Frenay, M., Freriks, I. L., Cofino, W. P. & Th. Brinkman, U. A. (1995). Survey of nine organotin compounds in the Netherlands using the zebra mussel (*Dreissena* *polymorpha*) as biomonitor. *Environmental Toxicology and Chemistry* **14**, 2023–2032.
560. *Stalter, D., Magdeburg, A. & Oehlmann, J. (2010). Comparative toxicity assessment of ozone and activated carbon treated sewage effluents using an *in vivo* test battery. *Water Research* **44**, 2610–2620.
561. *Štambuk, A., Pavlica, M., Vignjević, G., Bolarić, B. & Klobučar, G. I. V. (2009). Assessment of genotoxicity in polluted freshwaters using caged painter's mussel, *Unio pictorum*. *Ecotoxicology* **18**, 430–439.
562. *Stangierski, J., Andrzejewski, W., Tomaszewska-Gras, J., Grzes, B., Konieczny, P. & Urbanska, M. (2018). Effect of washing on the quality of surimi-like preparation obtained from soft tissue of freshwater mussel *Sinanodonta woodiana* (Lea, 1834). *Journal of* *Aquatic Food Product Technology* **27**, 961–974.
563. *Sternecker, K., Geist, J., Beggel, S., Dietz-Laursonn, K., De La Fuente, M., Frank, H.-G., Furia, J. P., Milz, S. & Schmitz, C. (2018). Exposure of zebra mussels to extracorporeal shock waves demonstrates formation of new mineralized tissue inside and outside the focus zone. *Biology Open* **7**, bio033258.
564. *Storey, A. W. & Edward, D. H. D. (1989). The freshwater mussel, westralunio carteri iredale, as a biological monitor of organochlorine pesticides. *Marine and Freshwater Research* **40**, 587–593.
565. *Stoyanova, S., Mollov, I., Velcheva, I., Georgieva, E. & Yancheva, V. (2020). Cadmium and polyaromatic hydrocarbons exposure changes the condition indices in *Dreissena* *polymorpha* (Pallas, 1771): A case study. *Acta Zoologica Bulgarica* **15**, 141–146.
566. Strack, E. (2015). European Freshwater Pearls: Part 1-Russia. *The Journal of Gemmology* **34**, 580–592.
567. Strayer, D. L. (1999). Effects of alien species of freshwater mollusks in North America. *Journal of the North American Benthological Society* **18**, 74–98.
568. *Stuart, K. R., Eversole, A. G. & Brune, D. E. (2001). Filtration of green algae and cyanobacteria by freshwater mussels in the partitioned aquaculture system. *Journal of the World Aquaculture Society* **32**, 105–111.
569. Su, L., Cai, H., Kolandhasamy, P., Wu, C., Rochman, C. M. & Shi, H. (2018). Using the Asian clam as an indicator of microplastic pollution in freshwater ecosystems. *Environmental Pollution* **234**, 347–355.
570. *Su, L., Xue, Y., Li, L., Yang, D., Kolandhasamy, P., Li, D. & Shi, H. (2016). Microplastics in Taihu Lake, China. *Environmental Pollution* **216**, 711–719.
571. *Sun, Y., Hayakawa, S., Ogawa, M., Naknukool, S., Guan, Y. & Matsumoto, Y. (2011). Evaluation of angiotensin I-converting enzyme (ACE) inhibitory activities of hydrolysates generated from byproducts of freshwater clam. *Food Science and Biotechnology* **20**, 303–310.
572. *Sures, B., Taraschewski, H. & Rydlo, M. (1997). Intestinal fish parasites as heavy metal bioindicators: A comparison between *Acanthocephalus lucii* (Palaeacanthocephala) and the Zebra Mussel, *Dreissena polymorpha*. *Bulletin of Environmental* *Contamination and Toxicology* **59**, 14–21.
573. *Sures, B., Zimmermann, S., Messerschmidt, J. & Von Bohlen, A. (2002). Relevance and analysis of traffic related platinum group metals (Pt, Pd, Rh) in the aquatic biosphere, with emphasis on palladium. *Ecotoxicology* **11**, 385–392.
574. *Szymanek, M. (2013). Palaeoecology of the Holsteinian Lake in vicinity of Wilczyn (eastern Poland) based on molluscan studies. *Geological Quarterly* **57**, 637–648.
575. Takabe, Y., Tsuno, H., Nishimura, F., Tanii, N., Maruno, H., Tsurukawa, M., Suzuki, M. & Matsumura, C. (2012). Bioaccumulation and primary risk assessment of persistent organic pollutants with various bivalves. *Water Science and Technology* **66**, 2620–2629.
576. *Tan, K., Xu, C. & Long, C. X. (2020). Association of microbiota in the stomach of *Sinanodonta* *woodiana* and its cultured soil. *3 BIOTECH* **10**, 319.
577. *Tang, J. Y., Dai, Y. X., Wang, Y., Qin, J. G., Su, S. S. & Li, Y. M. (2015). Optimization of fish to mussel stocking ratio: Development of a state-of-art pearl production mode through fish-mussel integration. *Aquacultural Engineering* **66**, 11–16.
578. *Taylor, A. M., Edge, K. J., Ubrihien, R. P. & Maher, W. A. (2017). The freshwater bivalve *Corbicula* *australis* as a sentinel species for metal toxicity assessment: An *in situ* case study integrating chemical and biomarker analyses. *Environmental Toxicology and* *Chemistry* **36**, 709–719.
579. *Tevesz, M. J. S., Matisoff, G., Frank, S. A. & McCall, P. L. (1989). Interspecific differences in manganese levels in freshwater bivalves. *Water, Air, and Soil Pollution* **47**, 65–70.
580. Tevesz, M. J. S., Smith, J. E., Coakley, J. P. & Risk, M. J. (1997). Stable carbon and oxygen isotope records from Lake Erie sediment cores: mollusc aragonite 4600 BP–200 BP. *Journal of Great Lakes Research* **23**, 307–316.
581. Theler, J. L. & Hill, M. G. (2019). Late Holocene shellfish exploitation in the Upper Mississippi River valley. *Quaternary International* **530**, 146–156.
582. Thitiphuree, T., Kitana, J., Varanusupakul, P. & Kitana, N. (2013). Atrazine contamination and potential health effects on freshwater mussel *Uniandra contradens* living in agricultural catchment at Nan Province, Thailand. *EnvironmentAsia* **6**, 13–18.
583. *Tran, D., Boudou, A. & Massabuau, J.-C. (2001). How water oxygenation level influences cadmium accumulation pattern in the asiatic clam *Corbicula fluminea*: A laboratory and field study. *Environmental Toxicology and Chemistry* **20**, 2073–2080.
584. Tran, D., Fournier, E., Durrieu, G. & Massabuau, J. C. (2007). Inorganic mercury detection by valve closure response in the freshwater clam *Corbicula fluminea*: integration of time and water metal concentration changes. *Environmental Toxicology and Chemistry* **26**, 1545–1551.
585. Tsai, J.-S., Lin, T., Chen, J. & Pan, B. (2006). The inhibitory effects of freshwater clam (*Corbicula fluminea*, Muller) muscle protein hydrolysates on angiotensin I converting enzyme. *Process Biochemistry* **41**, 2276–2281.
586. *Utida, G., Oliveira, E. C., Tucker, M., Petri, S. & Boggiani, P. C. (2017). Palaeoenvironmental interpretations based on molluscs from mid-Holocene lacustrine limestones, Mato Grosso do Sul, Brazil. *Quaternary International* **437**, 186–198.
587. *Valkova, E., Atanasov, V. & Veleva, P. (2020). Content of Fe and Mn in waters and zebra mussel (*Dreissena polymorpha*) from Ovcharitsa Dam, Stara Zagora region, Bulgaria. *Bulgarian Journal of Agricultural Science* **26**, 870–876.
588. *Valladão, G. M. R., De Pádua, S. B., Levy-Pereira, N., Farias, T. H. V. & Pilarski, F. (2017). Pathological assessment of exotic channel catfish infected by South American *Anodontites trapesialis* from Brazilian fish farm. *Aquaculture Research* **48**, 3975–3979.
589. Van Bocxlaer, B. (2020). Paleoecological insights from fossil freshwater mollusks of the Kanapoi Formation (Omo-Turkana Basin, Kenya). *Journal of Human Evolution* **140**, 102341.
590. *Van Plantinga, A. A. & Grossman, E. L. (2018). Stable and clumped isotope sclerochronologies of mussels from the Brazos River, Texas (USA): Environmental and ecologic proxy. *Chemical Geology* **502**, 55–65.
591. *Vangheluwe, M. L. U., Verdonck, F. A. M., Besser, J. M., Brumbaugh, W. G., Ingersoll, C. G., Schlekat, C. E. & Garman, E. R. (2013). Improving sediment-quality guidelines for nickel: Development and application of predictive bioavailability models to assess chronic toxicity of nickel in freshwater sediments. *Environmental Toxicology and Chemistry* **32**, 2507–2519.
592. *Varol, M. & Sünbül, M. R. (2017). Organochlorine pesticide, antibiotic and heavy metal residues in mussel, crayfish and fish species from a reservoir on the Euphrates River, Turkey. *Environmental Pollution* **230**, 311–319.
593. *Varol, M. & Sünbül, M. R. (2018). Biomonitoring of trace metals in the Keban Dam Reservoir (Turkey) using mussels (*Unio elongatulus eucirrus*) and crayfish (*Astacus leptodactylus*). *Biological Trace Element Research* **185**, 216–224.
594. *Vaughn, C. C., Atkinson, C. L. & Julian, J. P. (2015). Drought-induced changes in flow regimes lead to long-term losses in mussel-provided ecosystem services. *Ecology and Evolution* **5**, 1291–1305.
595. *Venugopal, A., Sudheer Kumar, C., Siva Kumar, N. & Swamy, M. J. (2017). Kinetic and biophysical characterization of a lysosomal α-L-fucosidase from the fresh water mussel, *Lamellidens corrianus*. *International Journal of Biological Macromolecules* **104**, 432–441.
596. *Versteegh, E. A. A., Troelstra, S. R., Vonhof, H. B. & Kroon, D. (2009). Oxygen isotope composition of bivalve seasonal growth increments and ambient water in the rivers Rhine and Meuse. *Palaios* **24**, 497–504.
597. *Versteegh, E. A. A., Vonhof, H. B., Troelstra, S. R. & Kroon, D. (2011). Can shells of freshwater mussels (Unionidae) be used to estimate low summer discharge of rivers and associated droughts?. *International Journal of Earth Sciences* **100**, 1423–1432.
598. *Vesk, P. A. & Byrne, M. (1999). Metal levels in tissue granules of the freshwater bivalve *Hyridella depressa* (Unionida) for biomonitoring: the importance of cryopreparation. *Science of the Total Environment* **225**, 219–229.
599. *Vidal, M.-L., Bassères, A. & Narbonne, J.-F. (2001). Interest of a multibiomarker approach in the assessment of freshwater ecosystem quality: Laboratory and field studies. *Water Science and Technology* **44**, 305–312.
600. *Villar, C., Stripeikis, J., D'Huicque, L., Tudino, M., Troccoli, O. & Bonetto, C. (1999). Cd, Cu and Zn concentrations in sediments and the invasive bivalves *Limnoperna fortunei* and *Corbicula fluminea* at the Rio de la Plata basin, Argentina. *Hydrobiologia* **416**, 41–49.
601. *Villela, I. V., de Oliveira, I. M., da Silva, J. & Henriques, J. A. P. (2006). DNA damage and repair in haemolymph cells of golden mussel (*Limnoperna fortunei*) exposed to environmental contaminants. *Mutation Research - Genetic Toxicology and Environmental Mutagenesis* **605**, 78–86.
602. *Vincent-Hubert, F., Arini, A. & Gourlay-Francé, C. (2011). Early genotoxic effects in gill cells and haemocytes of *Dreissena polymorpha* exposed to cadmium, B[a]P and a combination of B[a]P and Cd. *Mutation Research - Genetic Toxicology and Environmental Mutagenesis* **723**, 26–35.
603. *Voets, J., Talloen, W., de Tender, T., van Dongen, S., Covaci, A., Blust, R. & Bervoets, L. (2006). Microcontaminant accumulation, physiological condition and bilateral asymmetry in zebra mussels (*Dreissena polymorpha*) from clean and contaminated surface waters. *Aquatic Toxicology* **79**, 213–225.
604. *Vranković, J. (2015). Environmental impact on the antioxidant responses in *Corbicula fluminea* (Bivalvia: Veneroida: Corbiculidae) from the Danube River. *Italian Journal of Zoology* **82**, 378–386.
605. *Vuković-Gačić, B., Kolarević, S., Sunjog, K., Tomović, J., Knežević-Vukčević, J., Paunović, M. & Gačić, Z. (2014). Comparative study of the genotoxic response of freshwater mussels *Unio tumidus* and *Unio pictorum* to environmental stress. *Hydrobiologia* **735**, 221–231.
606. Waajen, G. W. A. M., Van Bruggen, N. C. B., Pires, L. M. D., Lengkeek, W. & Lürling, M. (2016). Biomanipulation with quagga mussels (*Dreissena rostriformis bugensis*) to control harmful algal blooms in eutrophic urban ponds. *Ecological Engineering* **90**, 141–150.
607. *Wagner, A. & Boman, J. (2004). Biomonitoring of trace elements in Vietnamese freshwater mussels. *Spectrochimica Acta - Part B Atomic Spectroscopy* **59**, 1125–1132.
608. *Wallace, J. S. & Blersch, D. M. (2015). Dynamic modeling predicts continued bioaccumulation of polybrominated diphenyl ethers (PBDEs) in smallmouth bass (*Micropterus dolomiu*) post phase-out due to invasive prey and shifts in predation. *Environmental Pollution* **206**, 289–297.
609. *Wang, G., Wang, X., Wu, L. & Li, X. (2012). Transformation and removal of organic matter and nitrogen by integrated ecological floating bed. *Jiangsu Daxue Xuebao (Ziran Kexue Ban)/Journal of Jiangsu University (Natural Science Edition)* **33**, 591–595.
610. *Wang, H., Qin, D., Sun, Y., Wang, P., Wang, Y. & Rui, Y. (2019). Study on the *Unio douglasiae* shell as an environmental indicator of heavy matals in the upstream of Songhua River. *Fresenius Environmental Bulletin* **28**, 271–279.
611. Wang, L., He, F., Sun, J., Hu, Y., Huang, T., Zhang, Y. & Wu, Z. (2017). Effects of three biological control approaches and their combination on the restoration of eutrophicated waterbodies. *Limnology* **18**, 301–313.
612. *Wang, L., Liu, P., Sun, J., Zhang, Y., Zhou, Q., Wu, Z. & He, F. (2018*a*). Comparison and combination of selective grazing on natural seston by benthic bivalves (*Hyriopsis cumingii*) and pelagic fish (*Hypophthalmichthys molitrix*). *Environmental Science and Pollution* *Research* **25**, 33423–33431.
613. *Wang, L., Ma, L., Sun, J., Zhang, Y., Zhou, Q., Wu, Z. & He, F. (2018*b*). Effects of different aquaculture methods for introduced bivalves (*Hyriopsis cumingii*) on seston removal and phosphorus balance at the water–sediment interface. *Journal of Freshwater Ecology* **33**, 251–265.
614. *Wang, P., Wang, R., Wang, C., Qian, J. & Hou, J. (2016). Exposure-dose-response relationships of the freshwater bivalve *Corbicula fluminea* to inorganic mercury in sediments. *Journal* *of Computational and Theoretical Nanoscience* **13**, 5714–5723.
615. Wang, X. X., Xie, L., Luo, C. & Wang, R. Z. (2006). Natural nacre coatings on titanium implant grown by fresh water bivalve shell. *Key Engineering Materials* **309**, 743–746.
616. *Wang, Y., Wang, W. L., Qin, J. G., Wang, X. D. & Zhu, S. B. (2009). Effects of integrated combination and quicklime supplementation on growth and pearl yield of freshwater pearl mussel, *Hyriopsis cumingii* (Lea, 1852). *Aquaculture Research* **40**, 1634–1641.
617. *Wang, Y. & Zhang, J. (2018). The influence of calcination temperature on the physicochemical characteristics of *Pteria martensii*. *Journal of Thermal Analysis and Calorimetry* **131**, 49–55.
618. *Wang, Y.-Y., Qiu, W.-Y., Sun, L., Ding, Z.-C. & Yan, J.-K. (2018). Preparation, characterization, and antioxidant capacities of selenium nanoparticles stabilized using polysaccharide–protein complexes from *Corbicula fluminea*. *Food Bioscience* **26**, 177–184.
619. *Warren, C. N. & Costa, J. D. (1964). Dating Lake Mohave artifacts and beaches. *American* *Antiquity* **30**, 206–209.
620. *Watanabe, M. F., Park, H.-D., Kondo, F., Harada, K.-I., Hayashi, H. & Okino, T. (1997). Identification and estimation of microcystins in freshwater mussels. *Natural Toxins* **5**, 31–35.
621. Watson, S. B. & Ridal, J. (2004). Periphyton: a primary source of widespread and severe taste and odour. *Water Science and Technology* **49**, 33–39.
622. *Waykar, B. & Deshmukh, G. (2012). Evaluation of bivalves as bioindicators of metal pollution in freshwater. *Bulletin of Environmental Contamination and Toxicology* **88**, 48–53.
623. *Waykar, B. & Shinde, S. M. (2011). Assessment of the metal bioaccumulation in three species of freshwater bivalves. *Bulletin of Environmental Contamination and Toxicology* **87**, 267–271.
624. *Webb, K., Craft, C. & Elswick, E. (2008). The evaluation of the freshwater western pearl mussel, *Margaritifera falcata* (Gould, 1850), as a bioindicator through the analysis of metal partitioning and bioaccumulation. *Northwest Science* **82**, 163–173.
625. *Wehrmeister, U., Jacob, D. E., Soldati, A. L., Hager, T. & Hofmeister, W. (2007). Vaterite in freshwater cultured pearls from China and Japan. *Journal of Gemmology* **30**, 399–412.
626. *Wei, Y., D’Errico, F., Vanhaeren, M., Li, F. & Gao, X. (2016). An early instance of upper Palaeolithic personal ornamentation from China: The freshwater shell bead from Shuidonggou 2. *PLoS ONE* **11**, e0155847
627. Weston, E., Szabó, K. & Stern, N. (2017). Pleistocene shell tools from Lake Mungo lunette, Australia: Identification and interpretation drawing on experimental archaeology. *Quaternary International* **427**, 229–242.
628. *Wiesner, L., Günther, B. & Fenske, C. (2001). Temporal and spatial variability in the heavy-metal content of *Dreissena polymorpha* (Pallas) (Mollusca: Bivalvia) from the Kleines Haff (northeastern Germany). *Hydrobiologia* **443**, 137–145.
629. *Wilson, W. A., Fritts, A. K., Fritts, M. W., Unrine, J. M. & Casper, A. F. (2018). Freshwater mussel (Unionidae) shells document the decline of trace element pollution in the regional watersheds of Chicago (Illinois, USA). *Hydrobiologia* **816**, 179–196.
630. *Winters, A. D., Marsh, T. L. & Faisal, M. (2011). Heterogeneity of bacterial communities within the zebra mussel (*Dreissena polymorpha*) in the Laurentian Great Lakes Basin. *Journal* *of Great Lakes Research* **37**, 318–324.
631. *Wojtal-Frankiewicz, A. & Frankiewicz, P. (2011). The impact of pelagic (*Daphnia longispina*) and benthic (*Dreissena polymorpha*) filter feeders on chlorophyll and nutrient concentration. *Limnologica* **41**, 191–200.
632. *Wong, K. W., Yap, C. K., Nulit, R., Hamzah, M. S., Chen, S. K., Cheng, W. H., Karami, A. & Al-Shami, S. A. (2017). Effects of anthropogenic activities on the heavy metal levels in the clams and sediments in a tropical river. *Environmental Science and Pollution Research* **24**, 116–134.
633. Wood, S. A., Briggs, L. R., Sprosen, J., Ruck, J. G., Wear, R. G., Holland, P. T. & Bloxham, M. (2006). Changes in concentrations of microcystins in rainbow trout, freshwater mussels, and cyanobacteria in Lakes Rotoiti and Rotoehu. *Environmental Toxicology* **21**, 205–222.
634. *Woźnicki, P., Lewandowska, R., Brzuzan, P., Ziomek, E. & Bardega, R. (2004). The level of DNA damage and the frequency of micronuclei in haemolymph of freshwater mussels *Anodonta woodiana* exposed to benzo[a]pyrene. *Acta Toxicologica* **12**, 41–45.
635. *Wu, C. F., Chen, C. H., Wu, C. Y., Lin, C. S., Su, Y. C., Wu, C. F., Tsai, H. P., Fan, P. S., Yeh, C. H., Yang, W. C. & Chang, G. R. (2020). Quinolone and organophosphorus insecticide residues in bivalves and their associated risks in Taiwan. *Molecules* **25**, 3636.
636. *Wu, X., Wu, H., Gu, X., Zhang, R., Ye, J. & Sheng, Q. (2019). Biomagnification characteristics and health risk assessment of the neurotoxin BMAA in freshwater aquaculture products of Taihu Lake Basin, China. *Chemosphere* **229**, 332–340.
637. *Wu, Y., Zhou, Y., Qiu, Y., Chen, D., Zhu, Z., Zhao, J. & Bergman, Ǻ. (2017). Occurrence and risk assessment of trace metals and metalloids in sediments and benthic invertebrates from Dianshan Lake, China. *Environmental Science and Pollution Research* **24**, 14847–14856.
638. *Wurster, C. M. & Patterson W.P. (2001). Seasonal variation in stable oxygen and carbon isotope values recovered from modern lacustrine freshwater molluscs: Paleoclimatological implications for sub-weekly temperature records. *Journal of Paleolimnology* **26**, 205–218.
639. *Xia, T. & Liu, X. (2011). Copper and zinc interaction on water clearance and tissue metal distribution in the freshwater mussel, *Cristaria plicata*, under laboratory conditions. *Frontiers of Environmental Science and Engineering in China* **5**, 236–242.
640. *Xu, H., Lv, S., Jiang, S., Lu, J. & Lin, L. (2020). Radical scavenging activities of peptide from Asian clam (*Corbicula fluminea*) and its protective effects on oxidative damage induced by hydrogen peroxide in HepG2 cells. *Journal of Food Biochemistry* **44**, e13146.
641. *Xu, M., Wang, Z., Duan, X. & Pan, B. (2014). Effects of pollution on macroinvertebrates and water quality bio-assessment. *Hydrobiologia* **729**, 247–259.
642. *Yan, H., Dettman, D. L., Chen, J. & Shen, N. J. (2020). delta C-1(3) in *Corbicula fluminea* shells: Implication for dissolved inorganic carbon reconstruction. *Geochemical Journal* **54**, 71–79.
643. *Yan, H., Lee, X., Zhou, H., Cheng, H., Peng, Y. & Zhou, Z. (2009). Stable isotope composition of the modern freshwater bivalve *Corbicula fluminea*. *Geochemical Journal* **43**, 379–387.
644. *Yan, H., Li, Z., Lee, X., Zhou, H., Cheng, H. & Chen, J. (2012). Metabolic effects on stable carbon isotopic composition of freshwater bivalve shell *Corbicula fluminea*. *Chinese Journal* *of Geochemistry* **31**, 103–108.
645. *Yan, J.-K., Wang, Y.-Y., Qiu, W.-Y., Wang, Z.-B. & Ma, H. (2018). Ultrasound synergized with three-phase partitioning for extraction and separation of *Corbicula fluminea* polysaccharides and possible relevant mechanisms. *Ultrasonics Sonochemistry* **40**, 128–134.
646. *Yan, J.-K., Wang, Y.-Y., Qiu, W.-Y., Wu, L.-X., Ding, Z.-C. & Cai, W.-D. (2017). Purification, structural characterization and bioactivity evaluation of a novel proteoglycan produced by *Corbicula fluminea*. *Carbohydrate Polymers* **176**, 11–18.
647. *Yan, L.-l., Zhang, G.-f., Liu, Q.-g. & Li, J.-l. (2009). Optimization of culturing the freshwater pearl mussels, *Hyriopsis cumingii* with filter feeding Chinese carps (bighead carp and silver carp) by orthogonal array design. *Aquaculture* **292**, 60–66.
648. *Yancheva, V., Velcheva, I., Iliev, I., Vasileva, T., Bivolarski, V., Georgieva, E. & Stoyanova, S. (2020). Histochemical and biochemical alterations in zebra mussel *Dreissena* *polymorpha* (Pallas, 1771) after cadmium and polyaromatic hydrocarbons exposure. *Acta Zoologica Bulgarica* **15**, 155–164.
649. *Yang, J., Harino, H., Liu, H. & Miyazaki, N. (2008). Monitoring the organotin contamination in the Taihu Lake of China by bivalve mussel *Anodonta woodiana*. *Bulletin of* *Environmental Contamination and Toxicology* **81**, 164–168.
650. *Yang, J., Karrow, P. F. & Mackie, G. L. (2001). Paleoecological analysis of molluscan assemblages in two marl deposits in the Waterloo region, southwestern Ontario, Canada. *Journal of* *Paleolimnology* **25**, 313–328.
651. Yang, S., Peng, Z., Wang, L., Wang, T. & Yang, C. (2019). Calcinated shell powder from *Corbicula fluminea* as a natural antimicrobial agent for soybean curd (Tofu) preservation. *Food Science and Technology Research* **25**, 545–553.
652. *Yao, H.-T., Lee, P.-F., Lii, C.-K., Liu, Y.-T. & Chen, S.-H. (2018). Freshwater clam extract reduces liver injury by lowering cholesterol accumulation, improving dysregulated cholesterol synthesis and alleviating inflammation in high-fat, high-cholesterol and cholic acid diet-induced steatohepatitis in mice. *Food and Function* **9**, 4876–4887.
653. Yeh, K. T., Wu, W. T., Subeq, Y. M., Niu, C. C., Liao, K. W., Chen, I. H. & Lee, R. P. (2017). Effects of freshwater clam extract on fracture induced inflammation at early stage. *Experimental and Therapeutic Medicine* **14**, 5039–5044.
654. *Yokoyama, A. & Park, H.-D. (2002). Mechanism and prediction for contamination of freshwater bivalves (Unionidae) with the cyanobacterial toxin microcystin in hypereutrophic Lake Suwa, Japan. *Environmental Toxicology* **17**, 424–433.
655. *Yoloğlu, E. (2019). Investigation of metallothionein level, reduced GSH level, MDA level, and metal content in two different tissues of freshwater mussels from Atatürk Dam Lake coast, Turkey. *Chemistry and Ecology* **35**, 644–659.
656. *Yoloğlu, E., Uçkun, M. & Uçkun, A. A. (2018). Metal accumulation and biochemical variations in the freshwater mussels (*Unio mancus*) collected from Atatürk Dam Lake, Turkey. *Biochemical Systematics and Ecology* **79**, 60–68.
657. *Yoshimura, T., Izumida, H., Nakashima, R., Ishimura, T., Shikazono, N., Kawahata, H. & Suzuki, A. (2015). Stable carbon isotope values in dissolved inorganic carbon of ambient waters and shell carbonate of the freshwater pearl mussel (*Hyriopsis* sp.). *Journal of* *Paleolimnology* **54**, 37–51.
658. *Yu, Z., Gao, G., Wang, H., Ke, L., Zhou, J., Rao, P., Chen, T., Peng, Z., Zou, J. & Luo, S. (2020). Identification of protein-polysaccharide nanoparticles carrying hepatoprotective bioactives in freshwater clam (*Corbicula fluminea* Muller) soup. *International Journal* *of Biological Macromolecules* **151**, 781–786.
659. Yu, Z., Walker, K. N., Evenson, E. B. & Hajdas, I. (2008). Lateglacial and early Holocene climate oscillations in the Matanuska Valley, south-central Alaska. *Quaternary Science Reviews* **27**, 148–161.
660. *Yusseppone, M. S., Bianchi, V. A., Castro, J. M., Noya Abad, T., Minaberry, Y. S., Sabatini, S. E., Luquet, C. M., Rios de Molina, M. C. & Rocchetta, I. (2020). *In situ* experiment to evaluate biochemical responses in the freshwater mussel *Diplodon chilensis* under anthropogenic eutrophication conditions. *Ecotoxicology and Environmental Safety* **193**, 110341.
661. *Yux, B., Zhao, Z., Tang, R., Xiong, B., Wu, Z. L., Sus, Q. & Yao, W. Z. (2020). Assessment of the environmental purification of triangle sail mussel (*Hyriopsis cumingii*) in recirculating aquaculture systems. *Applied Ecology and Environmental Research* **18**, 3439–3454.
662. Zarykhta, V. V., Zhang, Z., Kholodkevich, S. V., Kuznetsova, T. V., Sharov, A. N., Zhang, Y., Sun, K., Lv, M. & Feng, Y. (2019). Comprehensive assessments of ecological states of Songhua River using chemical analysis and bivalves as bioindicators. *Environmental Science and Pollution Research* **26**, 33341–33350.
663. Zeng, Y., Li, Z., Wang, Q., Xu, C., Li, Y. & Tang, J. (2019). Metal accumulation in Asiatic clam from the Lower Min River (China) and implications for human health. *Frontiers of Earth Science* **13**, 361–370.
664. *Zhang, G. F., Luo, Y., Zhang, W. F., Fang, A. P., Ye, R. H., Ren, G. & Yang, S. B. (2019). The effect of two selected strains of mussels as donor or host on the color of cultivated pearls. *Journal* *of Shellfish Research* **38**, 363–369.
665. *Zhang, H., Culver, D. A. & Boegman, L. (2011). Dreissenids in Lake Erie: An algal filter or a fertilizer?. *Aquatic Invasions* **6**, 175–194.
666. *Zhang, H., Lei, G., Chang, F., Pu, Y., Fan, H., Lei, Y., Yang, M., Zhang, W. & Yang, L. (2008). Chronology of the shell bar section and a discussion on the ages of the Late Pleistocene lacustrine deposits in the paleolake Qarhan, Qaidam basin. *Frontiers of Earth Science* *in China* **2**, 225–235.
667. *Zhang, H., Xia, W.-S., Xu, Y.-S., Jiang, Q.-X., Wang, C.-X. & Wang, W.-J. (2013). Effects of spray-drying operational parameters on the quality of freshwater mussel powder. *Food and* *Bioproducts Processing* **91**, 242–248.
668. *Zhang, N., Wei, C. & Yang, L. (2013). Occurrence of arsenic in two large shallow freshwater lakes in China and a comparison to other lakes around the world. *Microchemical Journal* **110**, 169–177.
669. Zhang, R., Cui, B. & Huang, S. (2015). Degradation of forchlorfenuron by nitrification and denitrification reactions in the gut and shell biofilm of *Limnoperna fortunei*. *Ecotoxicology* **24**, 381–390.
670. *Zhang, X., Liu, Z., Jeppesen, E. & Taylor, W. D. (2014). Effects of deposit-feeding tubificid worms and filter-feeding bivalves on benthic-pelagic coupling: Implications for the restoration of eutrophic shallow lakes. *Water Research* **50**, 135–146.
671. *Zhang, Y., Hu, X. & Yu, T. (2012). Distribution and risk assessment of metals in sediments from Taihu Lake, China using multivariate statistics and multiple tools. *Bulletin of* *Environmental Contamination and Toxicology* **89**, 1009–1015.
672. *Zhao, L., Walliser, E. O., Mertz-Kraus, R. & Schöne, B. R. (2017). Unionid shells (Hyriopsis cumingii) record manganese cycling at the sediment-water interface in a shallow eutrophic lake in China (Lake Taihu). *Palaeogeography, Palaeoclimatology,* *Palaeoecology* **484**, 97–108.
673. *Zheng, X., Tang, J., Ren, G. & Wang, Y. (2017*a*). The effect of four microbial products on production performance and water quality in integrated culture of freshwater pearl mussel and fishes. *Aquaculture Research* **48**, 4897–4909.
674. *Zheng, X., Tang, J., Zhang, C., Qin, J. & Wang, Y. (2017*b*). Bacterial composition, abundance and diversity in fish polyculture and mussel–fish integrated cultured ponds in China. *Aquaculture Research* **48**, 3950–3961.
675. *Zheng, X., Zhang, D., Qin, J. & Wang, Y. (2018). The effect of C/N ratio on bacterial community and water quality in a mussel-fish integrated system. *Aquaculture Research* **49**, 1699–1708.
676. *Zhong, H., Kraemer, L. & Evans, D. (2013). Influence of body size on Cu bioaccumulation in zebra mussels *Dreissena polymorpha* exposed to different sources of particle-associated Cu. *Journal of Hazardous Materials* **261**, 746–752.
677. *Zhou, C., Huang, J.-C., Liu, F., He, S. & Zhou, W. (2018). Removal of selenium containing algae by the bivalve *Sinanodonta woodiana* and the potential risk to human health. *Environmental Pollution* **242**, 73–81.
678. *Zhou, Y., He, Q. & Zhou, D. (2017). Optimization extraction of protein from mussel by high-intensity pulsed electric fields. *Journal of Food Processing and Preservation* **41**, e12962.
679. Zhu, L., Wang, H., Xu, J., Lin, J. & Wang, X. (2011). Effects of nacre-coated titanium surfaces on cell proliferation and osteocalcin expression in MG-63 osteoblast-like cells. *African Journal of Biotechnology* **10**, 15387–15393.
680. *Zhu, Z.-Y., Liu, N., Liu, Y., Si, C.-L., Liu, R.-Q., Chen, J., Liu, C.-J., Liu, A.-J. & Zhang, Y.-M. (2012). Chemical analysis of a polysaccharide from *Cristaria plicata* (Leach). *International Journal of Food Sciences and Nutrition* **63**, 506–511.
681. Zieritz, A. & Aldridge, D. C. (2009). Identification of ecophenotypic trends within three European freshwater mussel species (Bivalvia: Unionoida) using traditional and modern morphometric techniques. *Biological Journal of the Linnean Society* **98**, 814–825.
682. Zieritz, A., Azam-Ali, S., Marriott, A. L., Nasir, N. A. B. M., Ng, Q. N., Razak, N. A. A. B. A. & Watts, M. (2018*a*). Biochemical composition of freshwater mussels in Malaysia: A neglected nutrient source for rural communities. *Journal of Food Composition and Analysis* **72**, 104–114.
683. *Zimmermann, S., Messerschmidt, J., Von Bohlen, A. & Sures, B. (2005). Uptake and bioaccumulation of platinum group metals (Pd, Pt, Rh) from automobile catalytic converter materials by the zebra mussel (*Dreissena polymorpha*). *Environmental* *Research* **98**, 203–209.
684. *Zimmermann, S. & Sures, B. (2018). Lessons learned from studies with the freshwater mussel *Dreissena polymorpha* exposed to platinum, palladium and rhodium. *Science of the* *Total Environment* **615**, 1396–1405.
